# High-resolution targeted 3C interrogation of *cis*-regulatory element organization at genome-wide scale

**DOI:** 10.1038/s41467-020-20809-6

**Published:** 2021-01-22

**Authors:** Damien J. Downes, Robert A. Beagrie, Matthew E. Gosden, Jelena Telenius, Stephanie J. Carpenter, Lea Nussbaum, Sara De Ornellas, Martin Sergeant, Chris Q. Eijsbouts, Ron Schwessinger, Jon Kerry, Nigel Roberts, Arun Shivalingam, Afaf El-Sagheer, A. Marieke Oudelaar, Tom Brown, Veronica J. Buckle, James O. J. Davies, Jim R. Hughes

**Affiliations:** 1 MRC Molecular Haematology Unit, MRC Weatherall Institute of Molecular Medicine, University of Oxford, Oxford, UK; 2 MRC Weatherall Institute of Molecular Medicine, University of Oxford, Oxford, UK; 3 MRC WIMM Centre for Computational Biology, MRC Weatherall Institute of Molecular Medicine, University of Oxford, Oxford, UK; 4 Chemistry Research Laboratory, Department of Chemistry, University of Oxford, Oxford, UK; 5 Big Data Institute, Li Ka Shing Centre for Health Information and Discovery, University of Oxford, Oxford, UK; 6 Wellcome Centre for Human Genetics, Nuffield Department of Medicine, University of Oxford, Oxford, UK

**Keywords:** Biological techniques, Nuclear organization, Gene regulation, Chromatin structure

## Abstract

Chromosome conformation capture (3C) provides an adaptable tool for studying diverse biological questions. Current 3C methods generally provide either low-resolution interaction profiles across the entire genome, or high-resolution interaction profiles at limited numbers of loci. Due to technical limitations, generation of reproducible high-resolution interaction profiles has not been achieved at genome-wide scale. Here, to overcome this barrier, we systematically test each step of 3C and report two improvements over current methods. We show that up to 30% of reporter events generated using the popular in situ 3C method arise from ligations between two individual nuclei, but this noise can be almost entirely eliminated by isolating intact nuclei after ligation. Using Nuclear-Titrated Capture-C, we generate reproducible high-resolution genome-wide 3C interaction profiles by targeting 8055 gene promoters in erythroid cells. By pairing high-resolution 3C interaction calls with nascent gene expression we interrogate the role of promoter hubs and super-enhancers in gene regulation.

## Abstract (web summary)

Current 3C methods generate low-resolution interaction profiles across the genome or high-resolution profiles at a limited number of loci. Here the authors present Nuclear-Titrated Capture-C which produces high-resolution genome-wide interaction profiles.

## Introduction

Chromosome conformation capture (3C) has emerged as the leading tool for studying the DNA folding associated with gene regulation and genome organization^1,2^. 3C methods measure the proximity of DNA elements through restriction enzyme digestion and ligation; sequencing of the resultant chimeric fragments produces a population-based interaction frequency as the output. The resolution achieved by 3C comes from the choice of restriction enzyme, the depth of sequencing, and whether or not targeted enrichment is performed. Currently, 3C methods can be broadly categorized into two classes depending on their resolution: low and high.

Low-resolution 3C methods, such as Hi-C^3^ and its derivatives, use a 6-bp cutting enzyme to generate genome-wide interaction maps, with the standard experiment generating 10–50 kb resolution^2^. Higher-quality profiles can be achieved through combinations of massively increased sequencing, use of a 4-bp cutter, targeted enrichment (e.g., Capture Hi-C^4^ [CHi-C], often called Promoter Capture Hi-C), and increased cell numbers. The prohibitive costs mean that such datasets rarely include sufficient number of replicates for robust statistical analysis and are not applicable to rare primary cell types due to the requirement for high cell numbers. Conversely, sub-kilobase resolution can be achieved by methods which enrich for target loci in 4-base cutter libraries; e.g., Capture-C^5^, 4C-seq^6,7^, and their derivatives. The current best high-resolution 3C method for sensitivity is NG Capture-C, with 10,000–100,000+ unique interacting reporter reads per viewpoint^2,8^. NG Capture-C achieves its high resolution and sensitivity using biotinylated oligonucleotide pull-down of target loci from 3C material. The use of sequential enrichment, or double capture, results in 30–50% on-target sequencing, an 160-fold increase over the initial Capture-C method^5,8^.

High-resolution 3C comes at the expense of the number of viewpoints that can be practically included in a single experiment. This is due to the roughly 16-fold increase in complexity when generating a 3C library with a 4-bp cutter compared to a restriction enzyme with a 6-bp motif. The need to robustly sample these much more complex libraries has so far limited NG Capture-C to hundreds of viewpoints, performed in triplicate for statistical analysis. Because of these challenges, genome-scale characterization of promoter-enhancer interactions and their effects on transcription have so far been limited low-resolution methods, such as CHi-C, with one or two replicates. However, a large increase in the specificity of enrichment and the minimization of off-target and technical noise would practically translate into the feasibility of much larger viewpoint designs using high-resolution methods.

Here, using systematic optimization, we show that critical protocol modifications remove the throughput limitations of Capture-C by significantly reducing the levels of technical noise, and increasing the efficiency of on-target sequencing, while retaining the method’s capacity to multiplex samples and analyze small cell numbers. We combine these modifications and report the use of Nuclear-Titrated (NuTi) Capture-C to characterize the role of promoter hubs and super-enhancers in gene regulation by targeting 8055 promoters in erythroid cells.

## Results

### Nuclear isolation post ligation reduces the frequency of spurious interactions

**Fig. 1:**
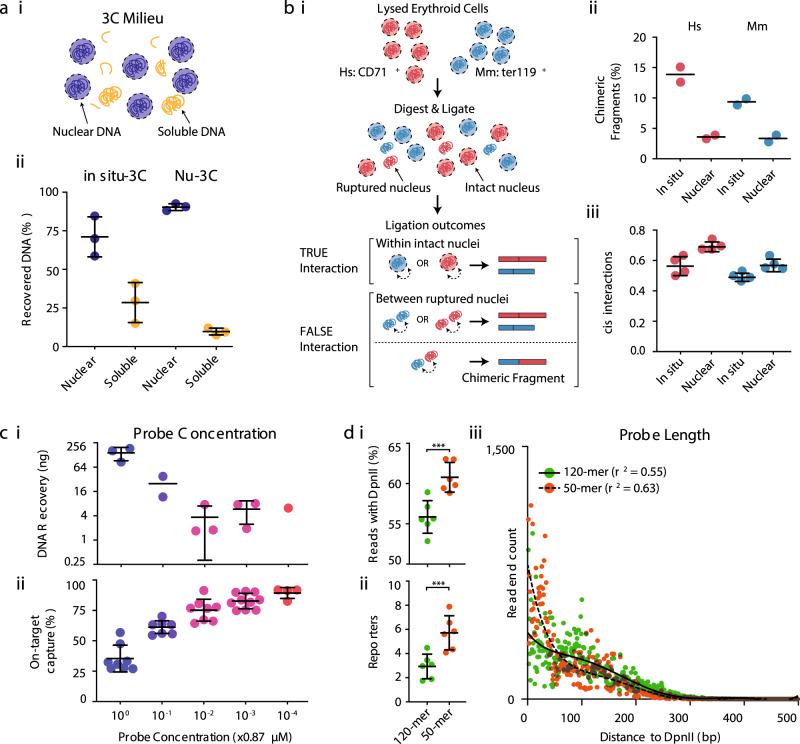
Nuclear-Titrated Capture-C minimizes noise while maximizing on-target enrichment. **a** (**i**) During digestion and ligation nuclei can shear leading to free soluble chromatin. Intact nuclei can be separated from freed material by centrifugation. (**ii**) Percent of total DNA recovered in the two fractions using standard in situ-3C and a modified Nuclear 3C (Nu-3C) approach. *n* = 3 independent experiments. Bars show mean and one standard deviation. **b** (**i**) Lysed erythroid cells from human and mouse were mixed in a 1:1 ratio prior to generation of 3C libraries. Ligation occurring between ruptured nuclei can be detected as inter-species chimeric DNA fragments after filtering for sequences that map to both genomes. (**ii**) Level of inter-species chimeras and (**iii**) number of reported *cis* interactions when using standard in situ-3C or modified Nuclear 3C (Nu-3C) at the *Hba-1/2* and *Slc25a37* promoters (*n* = 2 viewpoints from two independent libraries). Bars show mean and one standard deviation. **c** Total yield of DNA recovered following single capture (*n* ≥ 1 independent capture experiments where each dot is an independent capture) (**i**) and total number of mapped reads containing on-target capture sequence following double capture (*n* ≥ 4 libraries from multiple independent captures, where dots indicate libraries) (**ii**) when 11 probe pairs were used at final concentrations ranging from 0.87 µM to 87 pM. For DNA recovery each dot is a multiplex capture with between 3 and 6 libraries. Bars show mean and one standard deviation. **d** Percent of reads with a *Dpn*II site (**i**), number of PCR duplicate filtered reporters per 100 mapped reads containing a reporter (**ii**) following capture of six 3C libraries with 120-mer and 50-mer oligonucleotides. *n* = 6 independent experiments. ***p = 0.0001 using a two-sided Mann–Whitney *U*-test. Bars show mean and one standard deviation. (**iii**) Counts of read-ends generated by sonication breakpoints as the distance to the nearest end of the *Slc25a37* viewpoint. Each dot is average depth normalized count at each position for 100,000 mapped reads (*n* = 12). Lines of best fit were generated as a sixth order polynomial with *r*^2^ shown in the legend. Source data are available in the Source Data file.

The quality of 3C libraries can be affected by technical noise^9,10^. Previous work has shown that a portion of nuclei remain intact during 3C digestion and ligation, and intact nuclei contain more informative 3C DNA than disrupted nuclei^10,11^. Most 3C methods use the in situ^12^ protocol that assumes a majority of ligation events occur within intact nuclei, however, the frequency of ligation between two nuclei in in situ 3C libraries is unknown. By separating the in situ 3C milieu into intact nuclei and soluble DNA we found ~25% of in situ 3C libraries come from disrupted nuclei (Fig. 1a and Supp. Fig. 1). The portion of in situ 3C libraries from disrupted nuclei had higher levels of *trans* ligation and an increased proximal signal (<4 kb) at the expense of informative intermediate- and long-range interactions (Supp. Fig. 2). The higher rate of *trans* ligation likely arises from ligation of DNA from two separate nuclei.

True *trans* interactions may occur specifically within a cell^13^, or more generally at the boundaries of chromosome territories. To directly measure the frequency of ligation between nuclei during in situ 3C we generated libraries from a 1:1 admixture of human and mouse erythroid cells (Fig. 1b). Using this method, 50% of ligations between nuclei will connect DNA from mouse and human cells, generating detectable chimeric inter-species fragments. We found inter-species ligations represented 10–15% of reporter containing fragments (Fig. 1b.ii); therefore 20–30% of in situ 3C reporters arose from ligation of DNA from two separate nuclei. These reporters are artefacts, which lack biological relevance. This is consistent with ~25% of in situ 3C DNA being found in the un-pelleted supernatant and may account for a majority of detected *trans* interactions.

This high rate of spurious ligation between nuclei suggests that 3C data quality could be improved by enrichment for intact nuclei. To achieve this we modified the in situ 3C protocol to reduce the likelihood of rupturing fixed nuclei, and critically introduced a centrifugation step to isolate intact nuclei after ligation; as opposed to before chromatin digestion and ligation (see “Methods”). Using this Nuclear 3C (Nu-3C) method we found a significant reduction in the amount of free DNA compared to in situ 3C, from ~25% to ~10% (Fig. 1a.ii). We also saw a reduction in inter-nuclear ligation events, from ~25% to ~8%, with a concurrent significant increase in informative *cis* interactions (Fig. 1b.ii–iii). Therefore, Nuclear 3C libraries represent a higher-quality starting product for quantifying biologically relevant interactions than in situ 3C libraries.

### Probe titration increases targeting efficiency

NG Capture-C was designed to capture target viewpoints with tens or hundreds of 120-bp biotinylated DNA oligonucleotides located at either one or both end(s) of a restriction endonuclease fragment; high enrichment is then achieved through double capture^8^. This method uses a commercial exome sequencing kit optimized to include several thousand oligonucleotides. We tested serial dilutions of probe concentration on capture efficiency while targeting 11 loci. Lower probe concentrations resulted in reduced yields of DNA following single capture (Fig. 1c.i). When a probe concentration of 0.87 nM was used, 31.61% on-target sequencing (Stdev = 2.00, *n* = 4) was achieved, similar to that of double capture without dilution^8^. When lower concentration probes were used in combination with double capture (Titrated Capture-C), 85–98% on-target sequencing was achieved (Fig. 1c.ii); indicating the two optimizations are additive. When this combined method with probes at 0.87 nM was applied to *Slc25a37* alone a 97.70% on-target sequencing was seen, equating to a 6.26-million-fold enrichment. Increased on-target sequencing reduces the required depth of sequencing required to identify informative reads. We in silico tested the number of raw reads required to generate high-quality profiles and found 250,000 reads are sufficient to exceed 30,000 unique interactions (Supp. Fig. 3) at sites where probes are used for each fragment end. This depth of signal is 2.1 times better than the original NG Capture-C method^8^, and 11.6 times better than for an equivalent depth of sequencing for UMI-4C^14^.

A reduced read requirement represents a significant saving in the overall cost of Capture-C-based experiments, which previously was a criticism of the method^12^. Another significant cost for NG Capture-C has been the 120-bp biotinylated oligonucleotides—though current pricing is significantly reduced. We performed capture with 50-bp oligonucleotides targeted to the well-characterized mouse globin and mitoferrin encoding genes. Shorter oligonucleotides generated reads with proportionally more *Dpn*II restriction sites resulting in significantly more informative reads per captured fragment (Fig. 1d). This increase in informative capture events had no major changes to the local profiles of *Hba-1/2* and *Slc25a37* (Supp. Figs. 4 and 5). However, at *Hbb-b1/2*, a total of four additional peaks of interaction were identified in both erythroid and ES cells, leading to reduced correlation between oligonucleotide lengths (Supp. Fig. 6). Analysis of the sequences underlying these peaks showed a higher proportion of sequence identity for the 50-bp oligonucleotides (Supp. Fig. 6b, d). Given the increased similarity and that these peaks were fragment specific, they are likely artefacts arising from additional capture of the highly sequence-related globin genes. Therefore, while short probes provide more informative capture, they can also generate interaction artefacts through reduced specificity in highly duplicated loci, which should be taken into account during the design phase.

### Enrichment generates significant bias between co-targeted sites

**Fig. 2:**
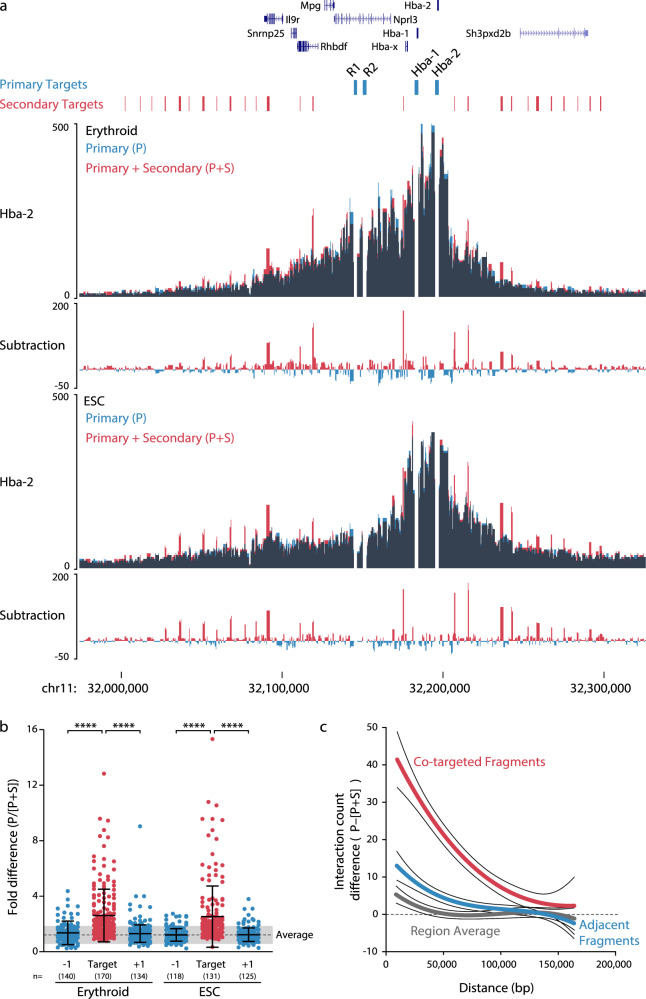
Co-targeting results in variable-magnitude, target-specific bias. **a** 3C libraries from mouse erythroid (*n* = 3 independent experiments) and embryonic stem cells (ESC; *n* = 3 independent experiments) were captured with either a pool of probes containing eight primary (P) viewpoints, or a pool of probes containing both the primary viewpoints and 54 additional, or secondary (S), viewpoints. Captured fragments were analyzed only for the primary viewpoints. Data is shown as an overlay for the Hba-2 capture viewpoint, with dark areas showing where signal overlaps. **b** Comparison of the relative difference in interaction counts at co-targeted fragments and the adjacent fragments (±1). Each dot (*n* shown) represents the average skew after capture in three independent 3C libraries from each of seven primary viewpoints. The difference in *n* between Erythroid and ESC arises from a poor mapability of the beta-globin locus in this ESC cell line. Average with one standard deviation (gray shading) is shown for all fragments within 160 kb of the primary targets. **c** Distance dependent difference in signal caused by co-targeting compared with adjacent fragments and the region average. ****p < 0.0001 using a two-sided Mann–Whitney *U*-test. Bars show mean and one standard deviation. Source data are available in the Source Data file.

Ligation frequency is the core readout of 3C techniques; many approaches use targeted enrichment through either oligonucleotide pull-down (NG Capture-C^8^, CHi-C^4^), immunoprecipitation (HiChIP^15^, ChIA-PET^16^, ChIA-Drop^17^) or RNA enrichment (HiChIRP^18^) to generate this readout. The introduction of bias to observed ligation frequency when using 3C experiments that enrich at multiple sites (i.e., co-targeting) is widely acknowledged^5,12^. Although enrichment bias will affect the accuracy of interaction calls, its magnitude has never been specifically measured. We first generated a mathematical model for enrichment-based bias (Supp. Note), which shows enrichment bias will be variable across the genome, ranging from 1-to-20 fold, and affected by both the true interaction frequency of co-targeted fragments and their relative enrichment efficiencies. We tested this model using two captures at the well-characterized mouse globin loci^19,20^. In the first capture, four promoters and three enhancers were targeted; in the second capture, an additional 54 evenly spaced targets were included^21^. The addition of the nearby oligonucleotides generated significant bias; specifically at co-targeted fragments, with a magnitude and distribution consistent with modeling (Fig. 2 and Supp. Fig. 7a), indicating the model is a good approximation for co-targeting bias. Similar bias is also seen in published CHi-C data and interaction calls^22^ (Supp. Fig. 7b). As bias magnitude is highly variable, rather than attempting complex correction, it may be simpler to avoid artefacts by simply removing co-targeted fragments from downstream analyses, ~3% of reporter counts in the above experiment (Supp Fig. 8a). For high-resolution 3C the exclusion of co-targeted fragments is unlikely to be a significant source of novel bias. Significant interactions tend to be called across multiple adjacent fragments^8^, and unlike low-resolution enzymes (e.g., *Hind*III 6-base cutter), the majority of regulatory elements have multiple *Dpn*II sites (Supp. Fig. 8b), thus, interactions between co-targeted elements are still detectable. Using an exclusion approach when targeting all 94,450 annotated mouse transcription start sites would only require 0.93% of *Dpn*II fragments to be excluded (59,575/6,415,222), compared with 5.61% if using low-resolution *Hind*III (46,227/823,377). Therefore, high-resolution designs with thousands of viewpoints are possible, provided the correct data analysis is used to avoid bias.

### High-resolution interaction maps for 8055 promoters

**Fig. 3:**
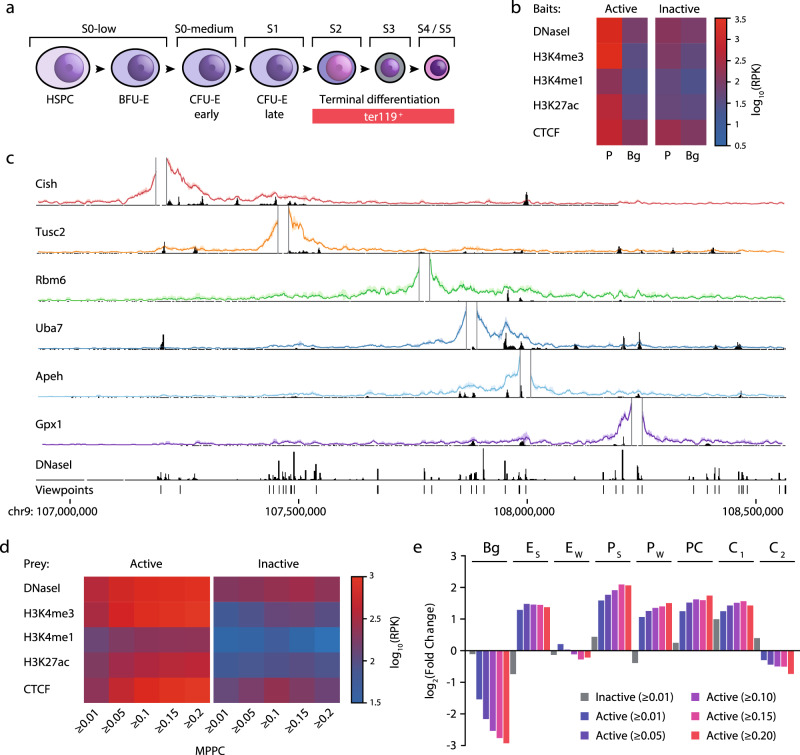
Genome-wide high-resolution 3C in mouse erythroid cells. **a** Stages (S) of commited murine erythropoiesis with FACS-sorted populations including haematopoietic stem and progenitor cells (HSPC), erythroid blast forming units (BFU-E), and colony-forming units (CFU-E). **b** Average sequence coverage signature of promoter (P) containing fragments (±1 kb) classified as active (*n* = 7014) or inactive (*n* = 181) compared to an equivalent number of background regions (Bg). Chromatin marks from mouse erythroid cells show open chromatin (DNaseI), promoters (H3K4me3), active transcription (H3K27ac), enhancers (H3K4me1), and boundaries (CTCF). Background (Bg) signal was calculated by generating random peaks of the same number and size using BEDtools shuffle. RPK reads per kilobase. **c** Windowed mean 3C interactions (*n* = 3 independent 3C libraries) over 1.5 Mb (mm9: chr9:106926158-108566246) for six NuTi Capture-C viewpoints with peaky Marginal Posterior Probability of Contact (MPPC) scores (black peaks) and open chromatin (DNaseI). **d** Average chromatin signal for interacting fragments (prey) of increasing MPPC identified by capturing either active or inactive promoters. **e** Enrichment of GenoSTAN annotations for interacting fragments with increasing MPPC. Bg: Background, *E*_S_: Enhancer (Strong H3K27ac), *E*_W_: Enhancer (Weak H3K27ac), *P*_S_: Promoter (Strong H3K27ac), *P*_W_: Promoter (Weak H3K27ac), PC: Promoter/CTCF, C_1_: CTCF near Promoter/Enhancer, C_2_: CTCF. Source data are available in the Source Data file.

As far as we are aware, no method has yet been implemented to generate high-resolution 3C maps for thousands of loci in triplicate. By combining higher-quality Nu-3C libraries, low-cell optimizations^9,23^, increased efficiency targeting through Titrated Capture-C, and a reduction in PCR cycles, our method, Nuclear-Titrated (NuTi) Capture-C (Supp. Fig. 1), has the capacity to generate reproducible high-resolution data in both small and genome-scale experiments. To this end we used DNaseI-seq and ChIP-seq for H3K27ac, H3Kme1, H3Kme3 signals from ter119^+^ mature erythroid cells^19,24^ to annotate tissue-specific transcription start sites of protein coding genes, identifying 7874 active promoters for targeting (Fig. 3a, b). We also included in the design a further 181 inactive control promoters, in total covering 7195 *Dpn*II fragments. Using this design, NuTi Capture-C was performed in triplicate for ter119^+^ erythroid cells and sequenced to an average of 150–300k read-pairs per viewpoint. We identified 140.8 M unique ligation events with over 1000 unique *cis*-ligation events for 93.5% of targets (*n* = 6730; Supp. Fig. 9a). We first compared the profiles of the well-characterized *Hba*-1/2, *Hbb-b1/2*, *Slc25a37* loci between small- and genome-scale capture designs (Supp. Fig. 10), finding good correlation between experiments (Pearson *r*^2^: 0.75–0.87) as well as between replicates (Pearson *r*^2^: 0.86–0.92). Interestingly, viewpoints shorter than 300 bp tended to have higher levels of *trans* interactions despite nuclear isolation (Supp. Fig. 11a, b). Quantification of non-nuclear DNA from *Hind*III and *Dpn*II 3C digestion found higher amounts of DNA from the 4-bp cutter (Supp. Fig. 11c, d). This suggests short fragments may either evade crosslinking, or be freed as small, diffusible fragments by digestion – resulting in the observed differences in *cis*-to-*trans* frequencies. Therefore, a minimum fragment length could be of benefit during viewpoint selection.

To identify significant distal interactions for each viewpoint we employed Bayesian modeling with peaky^25^ (Fig. 3c). Peaky identified 473,270 interacting fragment pairs (Marginal Posterior Probability of Contact [MPPC] ≥ 0.01) covering 75.8% of targeted viewpoints (*n* = 5451) and distributed between 2500 bp and 1 Mb from the midpoint of the target (Supp. Fig. 9b), with the majority being directly adjacent to another significantly interacting fragment (*n* = 404,552 [85.5%], Supp Fig. 8c). Identified promoter-interacting fragments had strong enrichment for chromatin marks associated with active promoters and enhancers, with stronger enrichment seen for fragments with higher MPPC scores (Fig. 3d). Despite excluding co-targeted viewpoints from these analyses, we were still able to detect over 9000 significant interactions with viewpoint adjacent fragments (Supp. Fig. 8d). Therefore, it is still possible to detect promoter–promoter interactions at co-targeted genes with NuTi Capture-C while avoiding co-targeting bias. To determine the identity of interacting regions we annotated 68,723 erythroid open-chromatin sites into eight classes using the GenoSTAN Hidden Markov Model^26^ (Supp. Fig. 12a, b). By intersecting significantly interacting fragments with these annotations we found 22,767 pairwise element interactions, involving 56.7% (*n* = 4082) of targeted genes (Supp. Fig. 12c, d). When comparing the types of elements active promoters interact with, we found specific enrichment for both promoters and enhancers (Fig. 3e and Supp. Fig. 9c), with each active gene interacting with an average of 2.6 promoters (stdev: 4.1, max: 43) and 1.3 enhancers (stdev: 2.4, max: 26).

### Targeted high-resolution 3C provides greater specificity of interaction calling

**Fig. 4:**
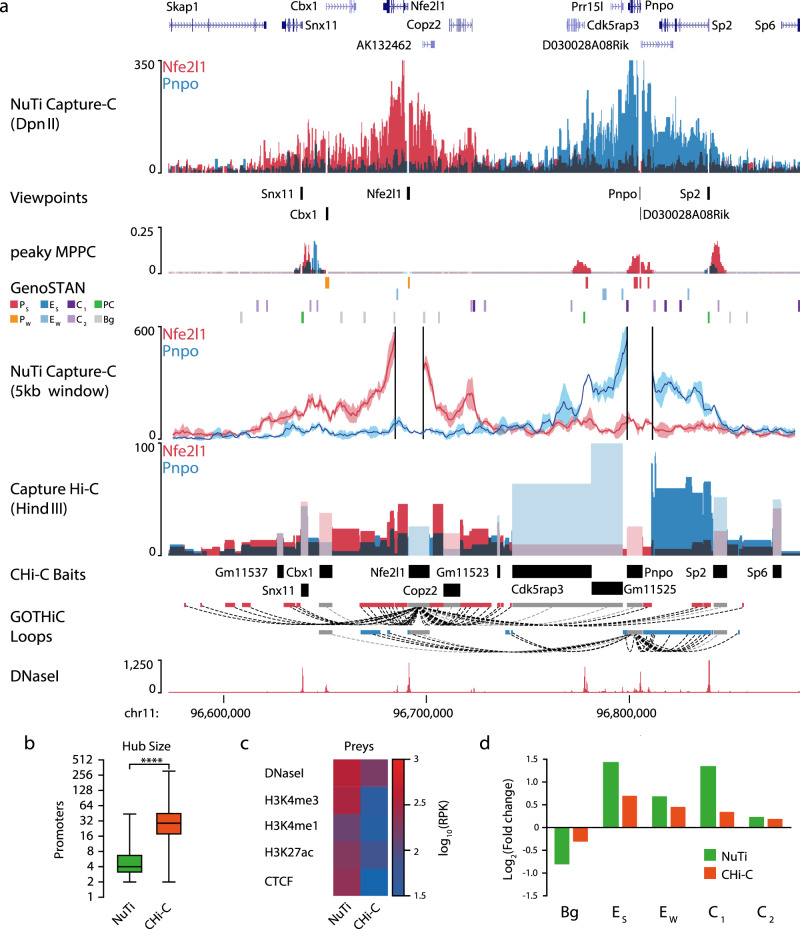
Comparison of capture resolution at the *Nfe2l1* and *Pnpo* promoters. **a** Sequence tracks showing the difference between high-resolution 3C (*Dpn*II, NuTi Capture-C) and low-resolution 3C (*Hind*III, Capture Hi-C) from nearby gene promoters (mm9, chr11:96,572,876-96,883,917) in erythroid cells. Tracks in order: UCSC gene annotation, *cis*-normalized mean interactions per *Dpn*II fragment using NuTi Capture-C (*n* = 3 independent 3C libraries), NuTi Capture-C viewpoints, peaky Marginal Posterior Probability of Contact (MPPC) scores with fragments with MPPC ≥ 0.01 darker, GenoSTAN open-chromatin classification, windowed mean interactions using NuTi Capture-C, total supporting reads per *Hind*III fragment with CHi-C (*n* = 2; co-targeted fragments are lighter in color), CHi-C bait fragments, loops between reported significantly interacting fragments (co-targeting loops are colored gray), erythroid tracks for open chromatin (DNaseI). Note overlapping blue and red signals appear darker in color (NuTi Capture-C, peaky MPPC, CHi-C). **b** Number of interacting promoters identified as present in promoter-hubs. ****p < 0.0001 a two-sided Mann–Whitney *U*-test. For NuTi *n* = 4339 promoter viewpoints, and for CHi-C *n* = 19,683 promoter viewpoints. Box and Whiskers show: minima 25th percentile, median, 75th percentile and maxima. **c** Average chromatin signature in mouse erythroid cells over fragments identified as being significantly interacting with promoters by NuTi Capture-C (Nu-3C) and Capture Hi-C (CHi-C). RPK: Reads per kilobase. **d** Enrichment of different classes of open-chromatin element in fragments identified as being significantly interacting with active promoters. *E*_S_: Enhancer (Strong H3K27ac), *E*_W_: Enhancer (Weak H3K27ac), C_1_: CTCF near promoter/enhancer, C_2_: CTCF, Bg: Background. Source data are available in the Source Data file.

The first descriptions of targeted genome-wide 3C landscapes were carried out using CHi-C with the low-resolution *Hind*III in duplicate in human CD34^+^ and GM12787 cells^4^, and in mouse ES cells and fetal liver cultured erythroid cells^22^. Currently, most publications using CHi-C employ the low-resolution *Hind*III enzyme on one or two replicates. To demonstrate the advantage of a high-resolution experiment in triplicate, we directly compared our NuTi Capture-C results with published CHi-C results in murine erythroid cells^22^. In general, the high-resolution method produced more fine-grained interaction profiles for promoters, including for genes in adjacent regulatory domains (Fig. 4a), and shared regulatory domains (Supp. Figs. 13–16), even when resolution is reduced with a 5 kb window (see Methods). The smaller fragment size also meant fewer fragments were affected by co-targeting bias, which provided more informative profiles in gene dense regions (Supp. Figs. 13, 16, 17). Notably, like CHi-C^22^ we could identify promoter-hubs, however we find significantly fewer constituent promoters (Fig. 4b), likely due to the removal of co-targeting bias from NuTi Capture-C analysis. Interaction calls generated using NuTi Capture-C also appeared more specific to functional elements than the broad regulatory domain calls of CHi-C (Supp. Figs. 13–29). NuTi Capture-C was more readily able to distinguish between regulatory elements, as *Dpn*II fragments are less likely to contain multiple functional elements than *Hind*III fragments (Supp. Fig. 30). Consistent with this, we found a higher level of active chromatin marks at interacting fragments identified with NuTi Capture-C (Fig. 4c). Finally we compared the types of annotated elements identified within interacting fragments. Given the high degree of co-capture bias observed with CHi-C, we focused on Promoter-Enhancer and Promoter-CTCF interactions. While both methods enriched for active enhancers, the extent of enrichment was greater in NuTi Capture-C (Fig. 4d). Therefore, NuTi Capture-C provides a technological advance for the generation of targeted genome-wide interaction maps and for interrogation of the organization of *cis-*regulatory elements.

### Enhancers predominantly co-locate upstream or downstream of cognate promoters

**Fig. 5:**
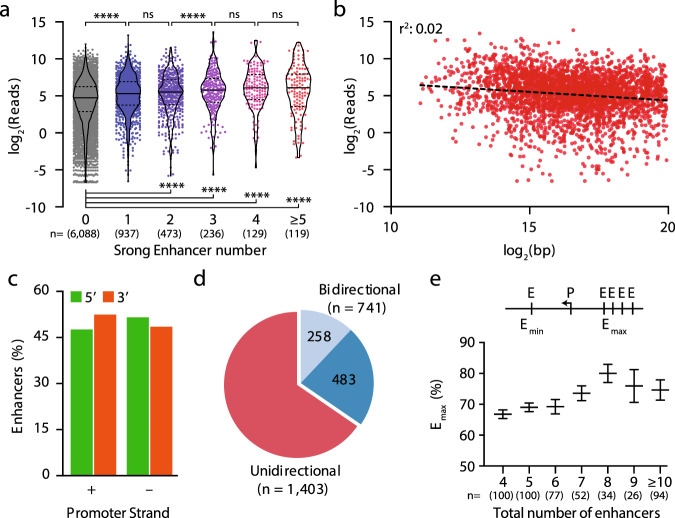
Enhancers show directional organization. **a** S3 nascent expression (4sU-seq) of genes interacting with elements identified as being strong enhancers by GenoSTAN. ****p < 0.0001, nonsignificant (ns), one-way ANOVA with Sidak’s multiple comparison tests. **b** S3 nascent expression of genes plotted against closest enhancer element. Line shows linear regression, *r*^2^ values is for Pearson correlation (*r* = −0.146, *p* < 0.0001). **c** Distribution of enhancer location (5’ or 3’) based on promoter strand (+ or –). **d** Number of promoters with multiple enhancers where the enhancers are all upstream or downstream (unidirectional: co-location) or a mixture of upstream and downstream (bidirectional). Bidirectional promoters with two or three enhancers (*n* = 258) are distinguished from those with four or more enhancers (*n* = 483) by light and dark blue, respectively. **e** Percent of enhancers on the side with the most enhancers (*E*_max_) at bidirectionally interacting promoters. Bars show standard error of the mean. *n* = number of promoters. Source data are available in the Source Data file.

To further explore how specific promoter interactions found in ter119^+^ erythroid cells could regulate transcription we measured nascent gene expression in sorted cell populations throughout erythropoiesis using 4sU-seq^27^. Sorted populations^28–30^ included haematopoietic stem and progenitor and burst-forming unit-erythroid cells (S0-Low), early and late colony-forming unit-erythroid (CFU-E) cells (S0-Medium and S1, respectively), and maturing terminal differentiating cells (S2, S3), which are ter119 positive (Fig. 3a); S2 and S3 cells correspond to the cells in which 3C data was generated. We first examined the effect of enhancer number and distance on expression. Unsurprisingly, genes with enhancer interactions had a significantly higher mean expression than those without (Fig. 5a). This effect was enhanced by the addition of second or a third interacting enhancer, but not four or more enhancers. Next we looked at the effect of enhancer distance on gene expression, finding only a weak positive correlation between enhancer proximity and gene expression (Fig. 5b).

It has been known for sometime that CTCF orientation is important for boundary function^31^, whereas promoters and enhancers have historically been considered orientation-independent. As such, we were interested to use high-resolution 3C to explore the location of interacting enhancers relative to their cognate promoter. We first determined that interacting elements were equally distributed upstream and downstream of promoters, consistent with orientation independence (Fig. 5c). We identified 2144 promoters that interacted with multiple enhancers. Interestingly for 65.4% (*n* = 1403) of these, all of the interacting enhancers clustered in a single direction, with all enhancers either upstream or downstream of the promoter. Where promoters lay between numerous interacting enhancers (*n* ≥ 4), we observed a strong bias for the majority of the enhancers to cluster in a single direction (Fig. 5d, e). Specifically, at 71.4% of promoters (*n* = 345/483) there were at least two more enhancers in one direction than the other. This pervasively directional organization may suggest an evolutionary selection for the grouping of enhancers.

**Fig. 6:**
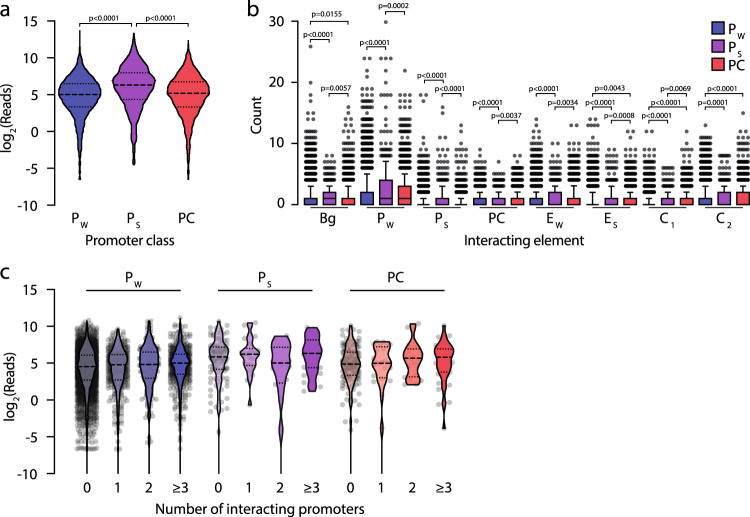
Promoter-hubs do not show synergy. **a** 4sU-seq measured expression of genes with promoters classified by GenoSTAN as having weak H3K27ac (*P*_W_), strong H3K27ac (*P*_S_), or a CTCF (PC). **b** Boxplots of interacting elements for each promoter class show 10th percentile (lower whister), 25th percentile, median, 75th percentile and 90th percentile (upper whisker). For **a** and **b**, *p*-values are for a two-sided Kruskal–Wallis test with Dunn’s multiple test correction. *n* = 5498 *P*_W_, 403 *P*_S_, and 1905 PC promoter viewpoints. **c** Expression of genes that do not interact with enhancers grouped according to the number of promoters that they interact with. An equivalent analysis for genes that interact with enhancers is in Supp. Fig. 31. Source data are available in the Source Data file.

Recent work has highlighted that some promoters may serve enhancer functions^32,33^, so we were interested to explore the relationship between promoter–promoter interactions and expression. Hidden Markov modeling (GenoSTAN^26^) of ChIP-seq signals in ter119^+^ cells (Supp. Fig. 12) identified three classes of promoter distinguished by H3K27ac level and CTCF binding (*P*_W_: Weak H3K27ac, *P*_S_: Strong H3K27ac, PC: CTCF Present). In S3 maturing terminal differentiating cells, genes with a *P*_S_ annotation had significantly higher expression than both *P*_W_ and PC-associated genes (Fig. 6a). When we compared the types and number of elements each class of promoter interacted with, *P*_S_ genes had more interactions with every other element class than *P*_W_ genes on average. This was particularly true for interaction with other promoters (Fig. 6b). Despite recent reports of enhancer-like promoters^32,33^, and in contrast to the additivity of enhancers, there was no difference in mean expression associated with increasing numbers of promoter–promoter contacts (Fig. 6c and Supp. Fig. 31). Therefore, the majority of promoter–promoter interactions may simply reflect presence of genes in transcription hubs or phase-separated bodies^34^ rather than functional co-regulation or synergy.

### Super-enhancer interaction alone does not drive tissue-specific expression

**Fig. 7:**
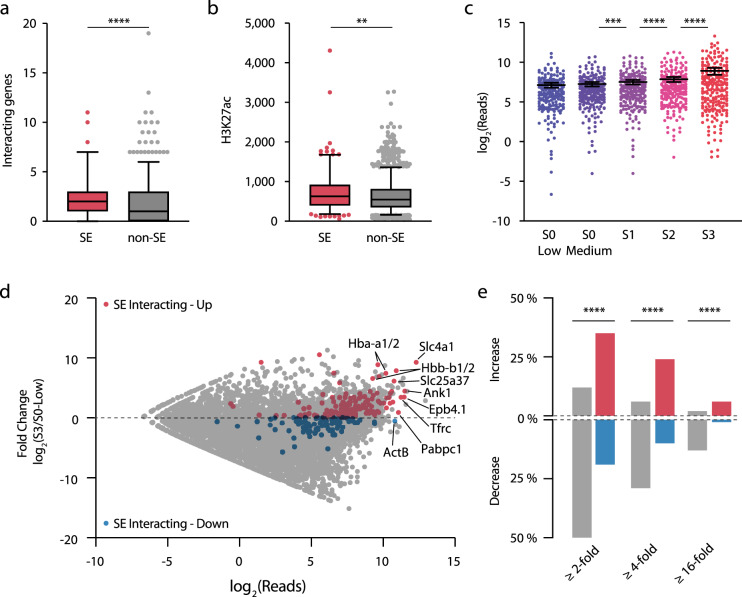
Super-enhancers interact with a diverse set of genes. **a** Intersection of mature erythroid super-enhancers (SE, *n* = 95) and non-super-enhancers (non-SE, *n* = 1172) with significant interacting fragments (MPPC ≥ 0.01) identified interacting genes. **b** Level of H3K27ac (fragment per kilobase per million mapped reads) over the promoters of significantly interacting genes interacting with SE (*n* = 226) and non-SE (*n* = 2042). For **a** and **b**, boxplots whiskers show 5–95 percentile and box shows 25th and 75th percentiles with the median, **p = 0.005, ****p < 0.0001 for a two-sided Mann–Whitney *U*-test. **c** Mean nascent expression for SE-interacting genes (*n* = 226 genes, each dot is the mean of three biological replicates) throughout erythroid differentiation was determined using 4sU-seq in haematopoietic stem and progenitor and burst-forming unit-erythroid cells (S0-Low), early and late colony-forming unit-erythroid cells (S0-medium and S1, respectively), and maturing terminal differentiating cells (S2, S3). Error bars show mean with 95% confidence interval. ***p = 0.0005, ****p < 0.0001 one-way ANOVA with Sidak’s multiple comparison test. **d** MA plot of expression in S0-Low and S3 cells with SE-interacting genes highlighted. **e** Percentage of total genes and SE-interacting genes with increased or decreased expression throughout differentiation. ****p < 0.0001 Chi-squared test (d.f. = 3). Source data are available in the Source Data file.

Super-enhancers have been identified as enhancers or clusters of enhancers (considered collectively), which have the highest occupancy of Med1 and H3K27ac^35,36^. It has been proposed that super-enhancers have a key role in controlling tissue-specific pathways and mammalian cell identity. Using our genome-wide high-resolution interaction calls, we identified 226 genes that interacted with 82/95 mature erythroid super-enhancers^19^. On average, mature erythroid super-enhancers interacted with more genes than other enhancers, and the promoters of these genes had higher levels of the active transcription mark, H3K27ac (Fig. 7a, b). However, the highest-ranking genes did not exclusively interact with super-enhancers, indicating that it is unlikely that super-enhancers are the only drivers of high-level transcription.

**Fig. 8:**
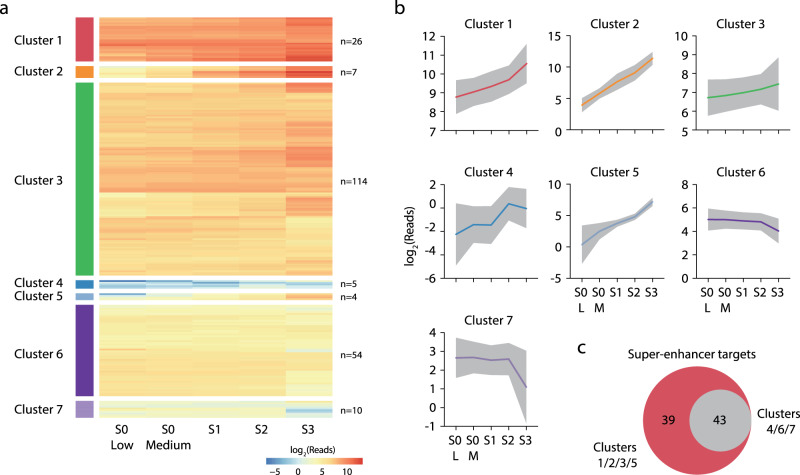
Temporal expression profiles of super-enhancer-interacting genes. **a** Heirachical clustering of nascent 4sU RNA-seq in haematopoietic stem and progenitor and burst-forming unit-erythroid cells (S0-Low), early and late colony-forming unit-erythroid cells (S0-medium and S1, respectively), and maturing terminal differentiating cells (S2, S3). **b** Mean expression of genes in each cluster, gray shading denotes one standard deviation. **c** Number of super-enhancers that interact with increasing expression clusters (1,2,3,5) or low expression and stable/decreasing clusters (4,6,7). Source data are available in the Source Data file.

Collectively, genes that interacted with erythroid super-enhancers increased in transcription throughout differentiation, with a significant increase in mean expression between each stage (Fig. 7c). To identify genes exhibiting high-levels of tissue-specific gene expression in mature erythroid cells we compared expression in S0-low and S3 cells. Erythroid super-enhancer-interacting genes were significantly enriched for genes showing increased expression, with almost one quarter showing at least a four-fold increase in transcription throughout differentiation (Fig. 7d, e) and 49.7% (*n* = 113) having significantly increased expression (DESeq2 *q* < 0.05). Despite this, several ubiquitously expressed house-keeping genes (e.g., *ActB* and *Pabpc1*) also interacted with mature erythroid super-enhancers. Notably, *ActB* expression did not increase throughout differentiation (Fig. 7 and Supp. Fig. 32) and 21 super-enhancer-interacting genes had significantly decreased expression. To characterize the expression of super-enhancer target genes we used hierarchical clustering (Fig. 8a, b). We identified seven gene expression profiles, four of which had increased expression at terminal erythropoiesis (68.6% of genes), and three clusters with stable, decreasing or low level expression (31.4% of genes). All 82 super-enhancers interacted with at least one gene in an increased expression cluster, while 43 also interacted with a gene with stable or decreasing expression (Fig. 8c). Therefore, super-enhancers interact with a diverse set of both tissue-specific and constitutively expressed genes, but interaction alone does not drive tissue-specific expression. This result is consistent with numerous studies in *Drosophila* showing that some level of functional compatibility is required between an enhancer and its cognate promoter^37–40^; a lack of compatibility may protect genes from the effects of super-enhancers.

## Discussion

Chromosome conformation capture is a powerful tool for the study of DNA folding within the nucleus. NG Capture-C has been applied to numerous biological questions, including enhancer characterization and super-enhancer dissection^19,41–44^, understanding the dynamics of Polycomb Bodies^45,46^ and X-chromosome inactivation^47,48^, characterizing CTCF boundaries^24,49,50^, and mapping the effector genes for polygenic human traits^51,52^. Despite their widespread applicability, the sequencing needs and cost of high-resolution methods have limited their use in large-scale experiments. To this end we have improved the scale upon which the Capture-C method can be employed. Our results show that efficiency gains can be made in both 3C library generation and in targeted enrichment. We have combined these technical improvements as NuTi Capture-C. Using NuTi Capture-C we generated high-resolution 3C interaction maps for over 8000 genes in triplicate from erythroid cells, the results of which are available online as a resource for red cell and genome biology researchers. Demonstrating that with thoughtful optimization of every stage of the process, the sensitive and versatile high-resolution 3C methods can be taken to a genome-wide scale.

In optimizing the production of 3C libraries, we found that the soluble and nuclear fractions of in situ 3C libraries have vastly different proximity signals and information content. Many statistical methods, including CHiCAGO^53^, peakC^54^, r3C-seq^55^, FourCSeq^56^ and peaky^25^, model this proximity decay curve to identify significant interactions. Our finding that the decay curve can be altered by technical fluctuation will be of particular concern when using these methods, especially when comparing different cell types, which may respond differently to fixation, lysis, digestion and ligation. Our solution to this was to isolate intact nuclei after ligation. This optimization also reduced the amount of noise from inter-nuclear ligation 3.3-fold, the majority of which would be reported as *trans* interactions. This generally applicable protocol adaptation would, therefore, likely improve any 3C method, leading to more reliable interaction calling, particularly as *trans* gene regulation through interaction has recently emerged as important for control of olfactory receptor genes^13^.

We have also robustly tested the effect of probe length, concentration, and pool composition for 3C enrichment. Shortening the length of probes delivered a predictable yield in higher informative sequencing content, with a concurrent risk of reduced specificity, whereas titrating the amount of probe increased the specificity of sequencing. The combination of nuclear isolation and probe titration has immediate and synergistic benefits, making possible very-large-scale 3C capture designs. One consideration when targeting multiple viewpoints is: would the same result be returned by targeting each viewpoint independently, or does co-enrichment skew the underlying interaction frequencies? Through modeling and experimental approaches, we show that co-enrichment in 3C methodologies does introduce significant amounts of bias. Disconcertingly, we find that the bias introduced by co-targeting is non-linear, and affected by both the relative efficiency of viewpoint enrichment and their true interaction frequency. Controlling for this bias is essential to avoid misleading results, such as a likely overinflated previous report of 250 significant promoter–promoter interactions per targeted promoter^22^. For biotinylated oligonucleotide capture, used in Capture-C and Capture Hi-C, co-targeting sites are specific and known. Therefore, bias can be avoided in these methods by using high-resolution enzymes, and masking interaction counts between co-targeted fragments. Bias introduced from methods where the target sites are not precisely defined, e.g., immunoprecipitation for ChIA-PET/HiChIP/ChIA-Drop^15–17^ and RNA purification enrichment for HiChIRP^18^, is considerably more complex and at present no such correction for co-enrichment skew is used in these methods. Our findings indicate that to accurately adjust for bias in these methods, researchers must determine the underlying interaction frequency, and the efficiency of targeting at each site. Realistically this could only be done by performing independent 3C (e.g., Hi-C) and enrichment (e.g., ChIP-seq) experiments prior to performing a now moot fusion experiment.

The technical advancements provided by NuTi Capture-C have allowed us to explore the organization of regulatory elements at a resolution and scale not reported before. Our results indicate that although the genome has 3D structure within the nucleus, linear arrangement may play an important role in gene expression. Our finding of a correlation between enhancer distance and expression is consistent with the loop-extrusion model of genome folding^57^ as proximal elements will be brought together more frequently by an extruding mechanism. A unidirectional extrusion model may also be responsible for the prevalent co-location of enhancers either upstream or downstream of promoters, an evolutionary force that may have driven the formation of super-enhancers. The finding that interacting with more than three enhancers provided little additional increase in expression may also provide insights into super-enhancers. Although it is clear at least some constituent parts of super-enhancers act in a simple additive manner^19^, this result suggests additivity is not the entire picture, and may be consistent with enhancers having distinct mechanistic roles or heirachy^58,59^. Therefore, high levels of transcription driven by multiple enhancers, and/or the effect of super-enhancers could be as a result of combining multiple enhancer functions. Interestingly, though not surprisingly, we also found that interaction with a tissue-specific super-enhancer is insufficient to drive a tissue-specific expression pattern. For example, the promoter of *Nprl3* lies adjacent to and interacts with the *α*-globin super-enhancer yet does not respond in the same tissue-specific manner^19^. It is likely that promoter-specific elements make them receptive to, and facilitate regulation by super-enhancers.

In this paper we have presented NuTi Capture-C, which provides an improved method for targeted high-resolution 3C experiments, used this method to develop a resource for the studying erythroid genetics, and explored at high-resolution the aspects of genome organization that control gene expression. The NuTi Capture-C protocol can be applied from a single locus up to the genome-wide scale, and as with the current Capture-C protocol is applicable to small cell number samples^9^. Additionally, individual developments that form part of the protocol address common steps in many 3C protocols and so could be implemented to improve the quality and reproducibility of other 3C techniques. Using this method, we expect researchers will be able to provide more reliable insights into biology while studying genome organization throughout growth, development, and in disease.

## Methods

### Cell culture and fixation

Protocols were approved through the Oxford University Local Ethical Review process. Experimental procedures were performed in accordance with European Union Directive 2010/63/EU and/or the UK Animals (Scientific Procedures) Act, 1986 under project licence 30/3339. All animals were singly housed, provided with food and water ad libitum, and maintained on a 12 h light: 12 h dark cycle (150–200 lux cool white LED light, measured at the cage floor), temperature: 21 °C +/−3 °C, humidity: 55 +/− 10%. Murine erythroid cells were obtained from spleens of C57BL/6 or C57BL/6-cross-CBA/J F1 hybrid mice treated with phenylhydrazine (40 mg/g body weight per dose, with three doses given 12 h apart; mice were killed on day 5). Spleens, consisting of >80% CD71^+^ ter119^+^ erythroid cells due to hemolytic anemia, were dissociated in Phosphate buffered solution (PBS) and strained through a 30 µM filter (Miltenyi Biotec) to remove clumps. For ter119^+^ selection, 3 × 10^8^ cells were resuspended in 3 mL of FACS buffer (PBS with 10% FBS) and stained with 0.9 µg anti-ter119-PE (130-102-338; Miltenyi Biotec). Stained cells were conjugated to anti-PE microbeads (130-048-801; Miltenyi Biotec) and passed through three LS Columns (Miltenyi Biotec). Mouse embryonic stem cells (ESC) from the feeder free line ES-E14TGA2a.IV (Strain 129/Ola) were grown on 0.1% gelatin (BHK-21 Glasgow Minimal Essential Medium (MEM) [21710025; Invitrogen], 10% Fetal bovine serum (FBS) [10270106; Invitrogen], 2 mM glutamine [25030024; Invitrogen], 100 U/mL Penicillin–Streptomycin [15140122; Invitrogen], 1 mM sodium pyruvate [11360039; Invitrogen], 1× MEM non-essential amino acids [11140035; Invitrogen], 0.1 mM 2-mercaptoethanol [31350010; Invitrogen], 1000 U/mL Leukemia Inhibition Factor) and resuspended with 0.05% trypsin for 5 min 37 °C before washing with PBS. Human erythroid cells were generated from CD34+ cells as described^51,60^ with ethics approval (MREC 03/08/097) and stored according to HTA guidelines (License 12433). Mouse erythroid and ESC were resuspended in RPMI (11875093; Invitrogen) with 15% FBS for fixation. Human erythroid cells were fixed in growth media. For all cell types, cells were resuspended at 1–2 × 10^6^ cells per mL and fixed at room temperature with 2% v/v formaldehyde for 10 min. Fixation was quenched with 120 mM glycine. Cells were washed with ice cold PBS before 3C library preparation.

### In situ 3C library preparation

In situ 3C libraries were prepared as previously described^8^; following fixation cells were lysed on ice in 5 mL lysis buffer (10 mM Tris-HCl, pH 8, 10 mM NaCl, 0.2% Igepal NP-40 (Sigma), 1× complete protease inhibitor (Roche) then pelleted by centrifugation (15 min, 4 °C, 1200 rcf). Lysis buffer was discarded and the pellet was resuspended in 1 mL lysis before snap freezing and storage at −20 °C for up to 12 months. For digestion, up to 2 × 10^7^ lysed cells were defrosted, pelleted (15 min, 4 °C, 1200 rcf) then resuspended in 650 µL 1× *Dpn*II buffer. Resuspended pellets were distributed into three digestion aliquots (200 µL each) and one digestion control (50 µL). Aliquots were then permeabilized with 0.28% sodium dodecyl sulfate (SDS); digestions (200 µL nuclei, 60 µL 10× *Dpn*II buffer, 434 mL PCR grade water, 10 µL 20% vol/vol SDS) undigested control (15 µL nuclei, 28.5 µL 10× *Dpn*II buffer, 227.5 mL PCR grade water, 4 µL 20% vol/vol SDS) for 1 h at 37 °C on a thermomixer (500 rpm). SDS was quenched into micelles for 1 h by addition of 20% Triton-X (1.67% final concentration, 66 µL for digest and 25 µL for the undigested control). *Dpn*II was added to digests in three aliquots of 10 µL (500 U) spaced several hours apart for a total digest time of 16–24 h at 37 °C. *Dpn*II was neutralized by incubation at 65 °C for 15 min and then immediate transfer to ice to reduce potential for de-crosslinking. One-hundred microliters was removed from each digestion reaction and combined as an un-ligated control. Controls were de-crosslinked, Proteinase-K treated, RNAse A treated, and phenol chloroform extracted as described for Nuclear 3C below. Crosslinked digested DNA was re-ligated by addition of 240 U T7 ligase to each reaction (500 mL PCR grade water, 134 mL 10× ligation buffer, 8 µL ligase) and incubated overnight at 16 °C on a thermomixer (500 rpm). De-crosslinked was performed overnight at 65 °C with 5 µL Proteinase-K (3 U). RNA was removed by treatment with 5 µL RNAse A (7.5 mU) for 30 min at 37 °C. DNA was extracted by addition of 4 mL phenol–chloroform–isoamylalcohol with thorough vortexing before centrifugation (10 min, 4200 rcf, room temp). The upper layer was transferred to a new tube and combined with 3.6 mL of chloroform, which was vortexed and centrifuged (10 min, 4200 rcf, room temp). DNA precipitated overnight at −20 °C by combinging the top layer with ethanol (7 mL water, 1 mL 3 M sodium acetate, 35 mL 100% ethanol). DNA was pelleted by centrifugation (30 min, 4200 rcf, 4 °C) and washed twice with 70% ice cold ethanol before resuspension in 300 µL water (30 µL for controls).

### Nuclear 3C library preparation

A full step-by-step method for NuTi Capture-C can be found on Protocol Exchange^61^. For Nu-3C, cells were lysed on ice in 5 mL lysis buffer then pelleted by centrifugation (15 min, 4 °C, 500 rcf). Lysis buffer was discarded and nuclei were resuspended in 1 mL PBS before snap freezing and storage at −20 °C for up to 12 months. For digestion, up to 5 × 10^6^ nuclei were defrosted, pelleted (15 min, 4 °C, 500 rcf) then resuspended in 215 µL 1× *Dpn*II buffer. Nuclei were then permeabilized with 0.28% SDS in a single reaction (200 µL nuclei, 60 µL 10× *Dpn*II buffer, 434 mL PCR grade water, 10 µL 20% vol/vol SDS) and one undigested control (15 µL nuclei, 28.5 µL 10× *Dpn*II buffer, 227.5 mL PCR grade water, 4 µL 20% vol/vol SDS) for 1 h at 37 °C on a thermomixer (500 rpm). SDS was quenched into micelles for 1 h by addition of 20% Triton-X (1.67% final concentration, 66 µL for digest and 25 µL for the undigested control). *Dpn*II was added to digests in three aliquots of 10 µL (500 U) spaced several hours apart for a total digest time of 16–24 h at 37 °C. *Dpn*II was neutralized by incubation at 65 °C for 15 min and then immediate transfer to ice to reduce potential for de-crosslinking. One-hundred microliters was removed from the digestion reaction and combined with 200 µL PCR grade water as an un-ligated control. Controls were de-crosslinked, Proteinase-K treated, RNAse A treated, and phenol chloroform extracted as described below. Crosslinked digested DNA was re-ligated by addition of 240 U T7 ligase (500 mL PCR grade water, 134 mL 10× ligation buffer, 8 µL ligase) and incubated overnight at 16 °C on a thermomixer (500 rpm). Following ligation, nuclei were isolated by centrifugation (15 min, 4 °C, 500 rcf), and the supernatant containing both freed DNA and the high levels of DTT from the ligation buffer, discarded. Nuclei were resuspended in 300 µL of TRIS-EDTA and de-crosslinked overnight at 65 °C with 5 µL Proteinase-K (3 U). RNA was removed by treatment with 5 µL RNAse A (7.5 mU) for 30 min at 37 °C. DNA was extracted by addition of 310 µL phenol–chloroform–isoamylalcohol with thorough vortexing before transfer to a phase-lock tube and centrifugation (10 min, 12,600 rcf, room temp). The upper layer was transferred to a new tube and DNA precipitated overnight at −20 °C (30 µL 3 M sodium acetate, 1 µL glycoblue, 900 µL 100% ethanol). DNA was pelleted by centrifugation (30 min, 21,000 rcf, 4 °C) and washed twice with 70% ice cold ethanol before resuspension in 150 µL water (30 µL for controls). To compare the 3C milieu with nuclear and soluble fractions, 2 × 10^6^ cells were processed following the standard in situ or Nuclear 3C method (Nu-3C, see below). Following ligation, half of the total volume (~2 mL) was removed for DNA extraction (3C milieu), the remaining volume was centrifuged (15 min, 15,000 rcf) and supernatant removed for DNA extraction (soluble fraction). The remaining pellet was resuspended in Tris-EDTA for DNA extraction. DNA extractions were then performed using phenol–chloroform–isoamylalcohol and ethanol precipitation.

### 3C library indexing

3C samples and controls were quantified using Qubit (Invitrogen), run on a 1% agarose gel and tested by qPCR with KAPA SYBR Fast (Sigma) to determine library quality. qPCR primers are in Supplementary Data 1. Only libraries with a digestion efficiency >70% were used for Capture-C. Libraries were either indexed with NEBNext DNA Library Prep Master Mix for Illumina (New England Biolabs) using 6 µg input 3C DNA as previously described following manusfacture’s instructions or using NEBNext Ultra II DNA Library Prep Kit for Illumina (New England Biolabs). When using the Ultra II kit 3 µg 3C material was sonicated to 200 bp using a Covaris S220 Focused Ultrasonicator, and purified using Ampure XP SPRI beads (Beckman Coulter). DNA was eluted into 53 µL with 1 µL used for D1000 TapeStation analysis (Agilent) and 2 µL used for Qubit quantification (Invitrogen). Fifty microliters of DNA ( ≤2 µg) was then indexed with the following modifications; for the End Prep reaction, the 20 °C incubation was lengthened to 45 min, 5 µL of NEBNext Adaptor was added and incubated for 30 min at 20 °C, the USER Enzyme incubation was extended to 30 min (37 °C), and indexing was performed in two reactions with Herculase II Fusion Polymerase (Agilent) using six cycles of amplification.

### Oligonucleotide synthesis and Titrated Capture-C

Pools of biotinylated oligonucleotides (Supp. Data 2) were sourced from IDT, Sigma or synthesized in house. We synthesized biotinylated oligonucleotides on a Combimatrix CustomArray B3 DNA synthesizer (B3Synth_v25.1 software) using CustomArray 12K Blank Slides (CustomArray Inc., PN: 2000100-Oligo pool Application). Probe sequences for 8055 genes were designed to be 70 bases in length and were placed at random positions on the microarray for synthesis using Layout Designer (v4.3.1). Synthesis of oligonucleotide probe sequences occurred on individual electrodes present on the semiconductor surface of the microarray by phosphoramidite chemistry in the 3′ to 5′ direction using standard software oligonucleotide pool synthesis settings and reagents prepared according to the manufacturer’s protocols. Each sequence was synthesized in triplicate. After the synthesis of the unmodified oligonucleotide, 5′-biotin was added using a double coupling cycle with an extended 15 min coupling time. The final detritylation step was performed manually using the software by incubating the slides with TCA deblock (4 × 30 s incubations) before washing the slide with acetonitrile four times and drying under argon. Oligonucleotides were then cleaved and deprotected on a stripping clamp system provided by the manufacturer using concentrated aqueous ammonia at 65 °C for 18 h. After cooling, the solution was recovered and the ammonia was removed by vacuum concentration. The oligonucleotide pool was purified using 2× illustra NAP-5 Columns (GE Life Sciences, PN: 17085302). The resulting solution was evaporated to dryness, resuspended in water and quantified by Nanodrop absorbance at 260 nm. Oligonucleotide pull-down for single and double capture of multiplexed 3C libraries was performed using the Nimblegen SeqCap EZ kit (Roche) following manufacturer’s instructions using a single reaction per library for primary capture and a single capture per pool for double capture, with appropriate masses of oligonucleotides and ten cycles of DNA amplification. For Titrated Capture-C, the stock concentration of oligonucleotides used in each capture reaction should be calculated by multiplying the number of unique oligonucleotides by 2.9 nM. For each capture reaction 4.5 µL of this stock is used, this equates to 13 fmol of each 120-mer oligonucleotide.

### Sequencing and data analysis

Fastq reads for small design captures were generated using paired-end sequencing (75/75, and 150/150 cycles) on either a MiSeq or NextSeq Illumina platform. The active gene design was sequenced by Novogene (Hong Kong) using 75/75 bp paired-end reads on the Illumina NovaSeq platform to generate at least 10^5^ read-pairs per viewpoint for each of the three libraries. Sequenced reads were processed using either CaptureCompendium, which incorporates CCseqBasic^62,63^ (v1.0), or a modified script (CCseqBasicM), which improves throughput for thousands of oligonucleotides by parallelizing analyses for groups of targets (available on Github: https://github.com/Hughes-Genome-Group/CCseqBasicM). To generate windowed plots, interaction counts for fragments were proportionally assigned to 250 bp bins and the average for each bin±2.5 kb (11 bins total) calculated using CaptureCompare^64^ (v1.0). Target enrichment was calculated as the percent of mapped read-pairs containing the target fragment divided by the total number of restriction endonuclease fragments in the genome. For sequencing depth analysis, deeply sequenced human data was used (GSE129378). Reporter counts were normalized to reporters per 100,000 *cis* reporter fragments and replicates combined using CaptureCompare^62,64^. Alignment of *Hbb-b1/2* oligonucleotides to off-target peaks was performed with Clustalω in MacVector (v15.0). Statistical comparisons were carried out using Prism. Genes were characterized as active or inactive using published H3K4me3, H3K27ac, DNaseI-seq, and RNA-seq data^19,24^. Peaky analysis was performed on the average reporter count per fragments as described^51^ with the following modification: to adjust for overcalling in bins with sparse data, residuals were normalized to have a mean of 0 and a standard deviation of 1 in each distance bin. We performed chromatin segmentation of ter119^+^ erythroid cells using GenoSTAN^26^ (v1.2.0). Segmentation used a peak centric approach, rather than signal across the whole genome, H3K4me1, H3K4me3, H3K27ac, and CTCF (GSE97871, GSE78835)^19,24^ was mapped with NGseqBasic^65^ and read coverage calculated (deepTools^66^, v2.4.2) for 1 kb windows over open-chromatin peaks (bedtools^67^ merge -d 10, v2.25.0) to capture histone modifications. The HMM model was trained using Poisson log-normal distributions with ten initial states. These were manually curated to eight final states based on similarity of chromatin signature.

### Nascent RNA-seq (4sU-seq)

Fetal livers were freshly isolated at e12.5-e13.5 from C57BL/6 mouse embryos. Primary erythroid progenitors were purified by FACS sorting lineage-negative cells based on CD71 and Ter119 levels as previously described^30,68^ and full step-by-step method can be found on Protocol Exchange^69^. Briefly, Fc receptors were blocked by immunostaining with rabbit IgG at 4 °C (200 μg/mL, Jackson Laboratories 015-000-003). Progenitor cells were enriched by stained with 5 μg/mL biotin-conjugated anti-Ter119 (BD 553672) for 30 min, before magnetic depletion using streptavidin nanobeads (BioLegend Mojosort 480016) following the manufacturer’s instructions. Cells were then incubated for 45 min with 0.5 μg/mL APC-conjugated streptavidin (BD 553672), 0.33 μg/mL PE-Cy7-conjugated anti-CD71 (BioLegend 113811) and a panel of five FITC-conjugated lineage antibodies (1 μg/mL each of: anti-CD41 [BD 553848], anti-CD45R [BD 553087], anti-CD3e [BD 553061], anti-CD11b [BD 557396] and anti-Ly-6G/6C [BD 553126]). Cells were then resuspended in FACS running buffer (PBS, 0.2% BSA, 5 mM Glucose, 2 mM EDTA) and 0.66 μg/mL Hoechst was added immediately prior to sorting in order to identify live cells. Cells were sorted on a BD FACSAria™ Fusion machine with a 100 μM nozzle size into microcentrifuge tubes containing PBS supplemented with 20% FBS and 2 mM Glucose. FACS-purified cells were rested for 6 h post-sort in erythroid media (IMDM supplemented with 20% FCS and 0.001% β-mercaptoethanol), then 500 µM 4‐thiouridine (4sU) was added to liquid cultures for 45 min. Cells were then pelleted by centrifugation at 200 rcf for 5 min and washed in PBS. The cell pellet was lysed in tri-reagent, and snap frozen on dry ice/ethanol. Total RNA was extracted from tri-reagent using a Direct-zol RNA kit (Zymo). 4sU labeled RNA was purified as previously described^27^ and full step-by-step method can be found on Protocol Exchange^70^. Briefly, 20–100 µg of labeled total RNA was added to 50 µL MTSEA-biotin-XX (0.1 mg/mL in dimethylformamide) and 25 µL of 10× Biotinylation buffer (100 mM Tris pH 7.4 10 mM EDTA) in a 250 µL reaction and rotated for 30 min at room temperature. Four-hundred microliters of chloroform–isoamylalcohol (24:1) was added to the reaction and incubated for 3 min. The aqueous phase was isolated using phase-lock gel tubes and purified by isopropanol precipitation. The RNA pellet was resuspended in 60 µL RNase free water, denatured at 65 °C for 10 min, and cooled on ice for 5 min. RNA was incubated with 60 µL streptavidin magnetic beads (Miltenyi) for 15 min at room temperature with rotation, applied to a µMACS column in a magnetic stand and washed with 1 mL MACS wash buffer (100 mM Tris pH 7.5, 10 mM EDTA, 1 M NaCl, 0.1% Tween 20) two times at 65 °C and two times at room temperature. RNA was eluted from the column by applying 100 µL of freshly prepared 100 mM dithiothreitol (DTT), followed by a second elution round 5 min later, then purified using a Qiagen RNeasy RNA clean-up kit with on-column DNase digestion, eluting in 20 µL of DEPC water. Libraries were prepared using the SMARTer® Stranded Total RNA-Seq Kit v2-Pico Input Mammalian (Takara Bio) following the manufacturer’s instructions with a fragmentation time of 3 min and 14 cycles of PCR amplification. Libraries were pooled and sequenced using 75-bp paired-end reads on the Illumina Next-Seq 2000 platform. Expression counts were generated using Kallisto^71^ (v0.43). Differential expression was determined using DESeq2 (Love 10.1186/s13059-014-0550-8.) and heirachical clustering was performed using pheatmaps (v1.0.12, Kolde 2012 https://CRAN.R-project.org/package=pheatmap).

### Reporting summary

Further information on research design is available in the Nature Research Reporting Summary linked to this article.

## Supplementary information

Supplementary Information

Supplementary Data 1

Supplementary Data 2

Description of Additional Supplementary Files

Reporting Summary

## Data Availability

Sequence reads and processed data for the active gene capture, sequencing depth capture, and expression data have been archived in the Gene Expression Omnibus (GSE160229, GSE129378, and GSE159229, respectively). Source Data for all figures are provided in the Source Data file. All other data supporting the findings of this study, including raw data files for optimization experiments are available from the corresponding author on request. Profiles for interactions of active genes in mouse erythroid cells are available at https://capturesee.molbiol.ox.ac.uk/projects/capture_compare/1086.

## Source data

Source Data

## Code availability

Scripts and code used in this work are available on Github: https://github.com/Hughes-Genome-Group/CCseqBasicM).
