# Supplementary material for: High-resolution targeted 3C interrogation of cis-regulatory element organization at genome-wide scale
Source: Nat Commun. 2021 Jan 22;12:531. doi: 10.1038/s41467-020-20809-6 (PMC7822813; doi:10.1038/s41467-020-20809-6)
Supplement: Supplementary file 1 — Supplementary Information [file 41467_2020_20809_MOESM1_ESM.pdf]

SUPPLEMENTARY INFORMATION

SUPPLEMENTARY NOTE

Mathematical modelling of 3C Enrichment Bias. pgs. 3-7

SUPPLEMENTARY FIGURES

Supp. Fig. 1 Capture-C workflows. pg. 8
Supp. Fig. 2 Soluble 3C material has a higher proximity signal. pg. 9
Supp. Fig. 3 Reporter sensitivity through sequencing depth pg. 10
Supp. Fig. 4 Capture of *Slc25a37* with short probes pg. 11
Supp. Fig. 5 Capture of α-globin locus with short probes pg. 12
Supp. Fig. 6 Capture of β-globin locus with short probes. pg. 13
Supp. Fig. 7 Co-targeting bias observed in Capture-C and Capture Hi-C. pg. 14
Supp. Fig. 8 Effect of excluding co-captured fragments from analysis. pg. 15
Supp. Fig. 9 Chromatin signature of captured promoters. pg. 16
Supp. Fig. 10 Genome scale capture closely matches designs with fewer probes. pg. 17
Supp. Fig. 11 Short fragments have higher levels of *trans* interactions. pg. 18
Supp. Fig. 12 GenoSTAN annotation of the mouse genome in erythroid cells. pg. 19
Supp. Fig. 13 NuTi Capture-C from the *Tp53, Wrap53* and *Mpdu1* promoters. pg. 20
Supp. Fig. 14 NuTi Capture-C from the *Hipk1, Dclre1b* and *Ap4b1* promoters. pg. 21
Supp. Fig. 15 NuTi Capture-C from the *Hba-1* and *Hba-2* promoters. pg. 22
Supp. Fig. 16 NuTi Capture-C from the *Hbb-b1* and *Hbb-b2* promoters. pg. 23
Supp. Fig. 17 NuTi Capture-C from the *Slc4a1* promoter. pg. 24
Supp. Fig. 18 NuTi Capture-C from alternative *Tmcc2* promoters. pg. 25
Supp. Fig. 19 NuTi Capture-C from the *Adrb2* promoter. pg. 26
Supp. Fig. 20 NuTi Capture-C from the *Gpcpd1* promoter. pg. 27
Supp. Fig. 21 NuTi Capture-C from the *Gypc* promoter. pg. 28
Supp. Fig. 22 NuTi Capture-C from the *Klf13* promoter. pg. 29
Supp. Fig. 23 NuTi Capture-C from the *Kras* promoter. pg. 30
Supp. Fig. 24 NuTi Capture-C from the *Rae1* promoter. pg. 31
Supp. Fig. 25 NuTi Capture-C from the *Slc25a37* promoter. pg. 32
Supp. Fig. 26 NuTi Capture-C from the *Tal1* promoter. pg. 33
Supp. Fig. 27 NuTi Capture-C from the *Tfrc* promoter. pg. 34
Supp. Fig. 28 NuTi Capture-C from the *Wnk1* promoter. pg. 35
Supp. Fig. 29 NuTi Capture-C from the *Ank1* promoter. pg. 36
Supp. Fig. 30 *Dpn*II provides higher resolution for distinguishing between functional elements than *Hind*III. pg. 37
Supp. Fig. 31 Promoter-hubs do not drive higher expression. pg. 38
Supp. Fig. 32 Expression of selected super-enhancer interacting genes. pg. 39

SUPPLEMENTARY NOTE: *Mathematical modelling of 3C enrichment bias.*

The number of interactions in 3C experiments are constrained by the fact each fragment can only ligate to two other fragments. Therefore, the total number of interactions is limited by the total number of cells, in effect, it is a closed system. To explore the effects of enrichment for multiple targets on 3C libraries we created a small closed system of fragments where each fragment has 5,000 interactions. Within this system, interactions involving three fragments, “A”, “B”, and “C”, can be sampled (i.e. enriched) with varying levels of efficiency. The remaining fragments can be collectively considered “X”, with “D” being one of these remaining fragments. The absolute number of interactions between each fragment within this system and example interaction profiles are demonstrated below (Supp. Note Table 1, Supp. Note Fig. 1). To demonstrate the effect on interaction calling, within this system “significant interactions” are simply those observed at a frequency of greater than 1 in 50 (>0.02). The significant interactions in this system are A-B, A-D, and B-D.

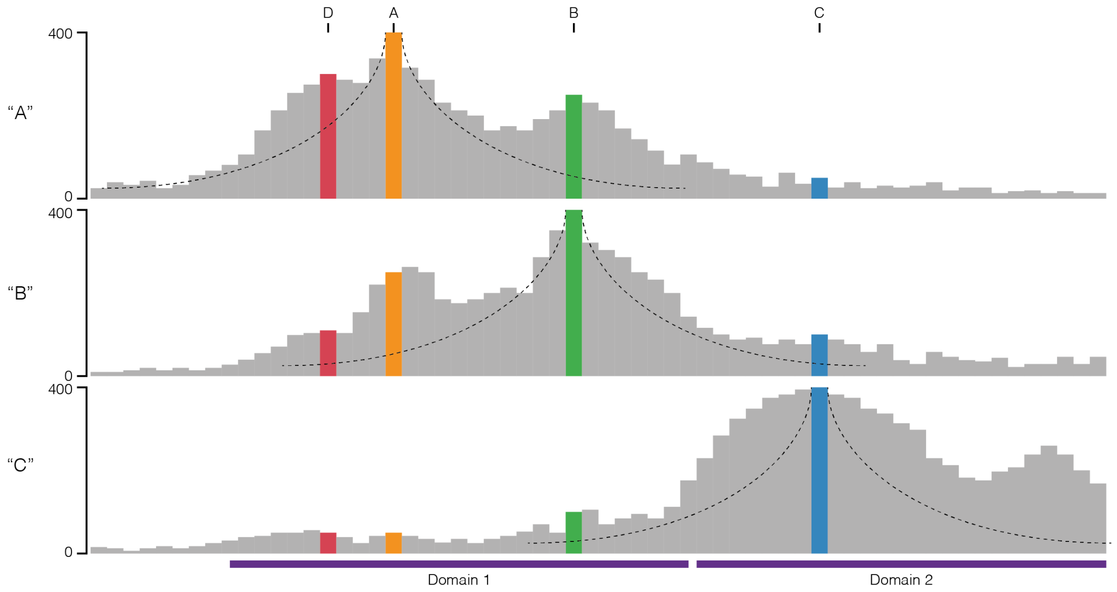

Supp. Note Fig. 1 I A closed system of interactions. Profile of absolute interaction counts for three sampleable fragments “A”, “B” and “C” within a closed system. The fragments separate into two interacting domains. Monotonic decay curves associated with the polymer models of interaction are shown.

Supp. Note Table 1. A closed system of interactions.

Viewpoint / Bait (Total count)		Interacting Fragment / Prey							
		Real Count				Real Frequency			
		“A”	“B”	“C”	“D”	“A”	“B”	“C”	“D”
	“A” (5,000)	–	250	50	300	–	**0.0500**	0.0100	**0.0600**
	“B” (5,000)	250	–	100	110	**0.0500**	–	0.0200	**0.0220**
	“C” (5,000)	50	100	–	50	0.0100	0.0200	–	0.0100

*Significant interactions (Freq. > 0.02) are shaded green.

The sampling within this system represents the targeted enrichment of 3C methods (e.g. probe hybridisation or immunoprecipitation). For 3C enrichment, efficiency can be affected by, among other things, the number of individual probes targeting each viewpoint and the melting points (e.g. Capture-C, CHi-C), and the level of target signal (e.g. Hi-ChIP, Hi-ChIRP, ChIA-PET). To represent this diversity in these processes, we assigned sampling of “A” and “C” to be highly efficient at 80% and 90% respectively. Whereas enrichment of “B” is relatively low at 10%. When each of these sampling efficiencies is applied to any one fragment at time, the total number of observed interactions decreases, however the frequency of interaction within the system remains constant (Supp. Note Fig. 2, Supp. Note Table 2). At this point, it is important to note that while the B-to-C count is lower than the C-to-B count, their proportional frequencies are still equal, and the same set of “significantly interacting” fragments are detected.

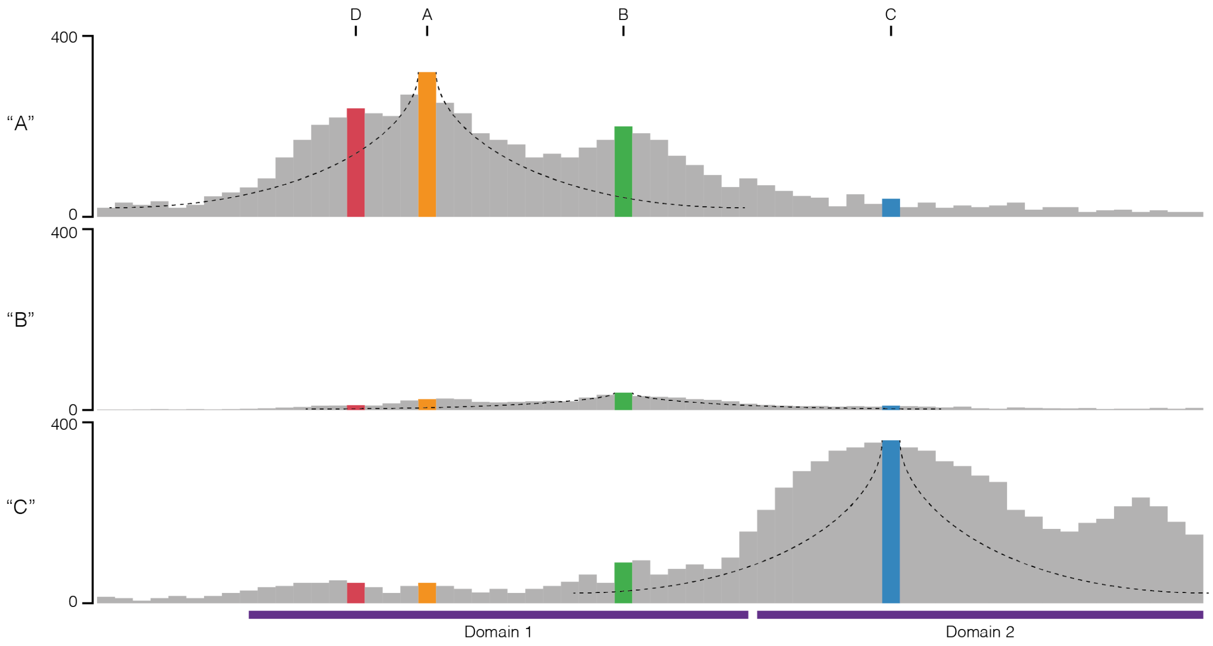

Supp. Note Fig. 2 I Independent sampling of interactions. Profile of observed interaction counts for three independently sampled fragments “A”, “B” and “C” within a closed system.

Supp. Note Table 2. Interaction counts following independent sampling.

		Interacting Fragment (Prey)											
		Observed Count				Observed Frequency				Observed Freq. / Real Freq.			
		“A”	“B”	“C”	“D”	“A”	“B”	“C”	“D”	“A”	“B”	“C”	“D”
Viewpoint / Bait (Total count)	“A” (4,000)	–	200	40	240	–	**0.0500**	0.0100	**0.0600**	–	1	1	1
	“B” (500)	25	–	10	11	**0.0500**	–	0.0200	**0.0220**	1	–	1	1
	“C” (4,500)	45	90	–	45	0.0100	0.0200	–	0.0100	1	1	–	1

*Significant interactions (Freq. > 0.02) are shaded green.

When we consider sampling fragment “A”, at 80% efficiency, we fail to see 50 “A-B” interactions, ten “A-C” interactions, and 60 “A-D” interactions. If we were to simultaneously sample (co-sample) the remaining viewpoints, from the missed interactions would can recover five “A-B” interactions (at 10% “B” sampling efficiency), nine “A-C” interactions (at 90% “B” sampling efficiency), and zero “A-D” interactions as it is unsampled. This recovery can be applied to each fragment as the first or second fragment sampled and leads to as much as a 9.1-fold increase in observed interaction counts. When the co-sampled fragments are presented as frequency values significant divergence from the true values within the system is observed (Supp. Note Fig. 3, Supp. Note Table 3). This divergence in frequency varies across each interacting pair and ranges from a 0.65-fold decrease (B-D) to a near 6-fold increase (B-C).

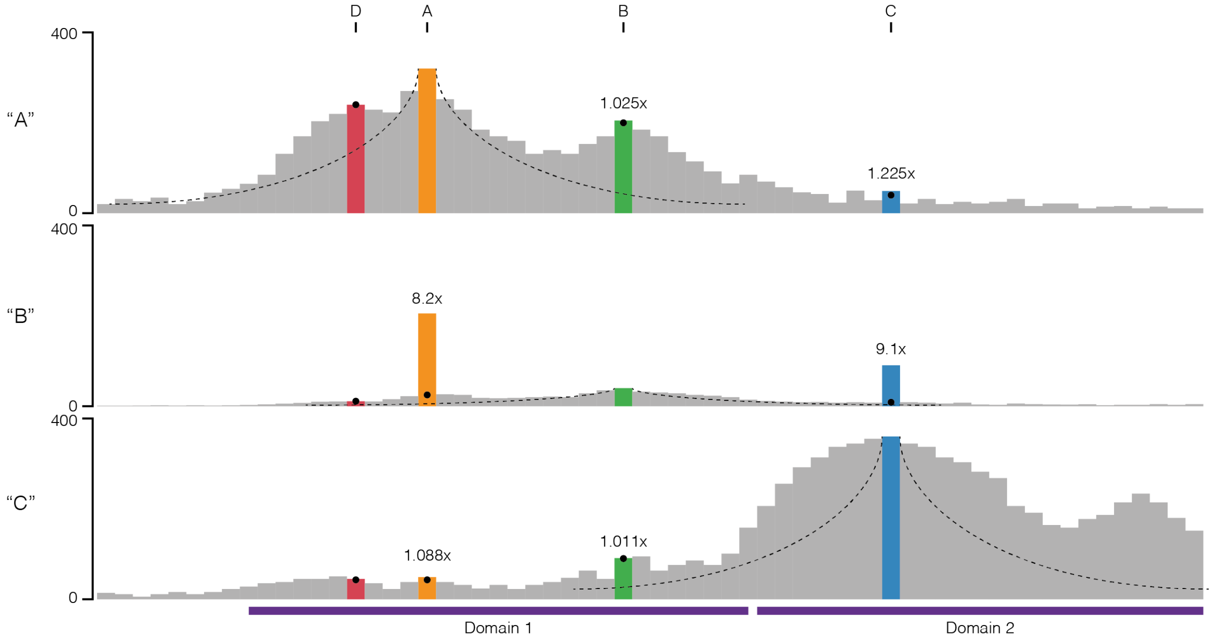

Supp. Note Fig. 3 I Co-sampling of interactions. Profile of observed interaction counts for three co-sampled fragments “A”, “B” and “C” within a closed system. Black circles represent counts observed with independent sampling, and the observed fold difference in raw counts is shown.

Note Table 3. Interaction counts following co-sampling.

		Interacting Fragment (Prey)											
		Observed Count				Observed Frequency				Observed Freq. / Real Freq.			
		“A”	“B”	“C”	“D”	“A”	“B”	“C”	“D”	“A”	“B”	“C”	“D”
Viewpoint / Bait (Total count)	“A” (4,014)	–	205	49	240	–	**0.0511**	0.0122	**0.0598**	–	1.021	1.221	0.997
	“B” (761)	205	–	91	11	**0.2694**	–	**0.1196**	0.0145	5.387	–	**5.978**	**0.657**
	“C” (4,505)	49	91	–	45	0.0109	**0.0202**	–	0.0100	1.087	1.009	–	0.998

*Significant interactions (Freq. > 0.02) are shaded green.

Interestingly, where previously the frequency of B-to-C matched C-to-B, it is now the count of interactions which is equal, and the observed frequency is unequal. This change in frequency also results in different significant interactions, B-D is no longer called (false negative), while B-C is now called (false positive). The effect on relative frequency of co-sampling is bi-directional: the observed frequency of interaction with co-sampled fragments increases, and the observed frequency of interaction with un-sampled fragments decreases. Therefore, significant and variable bias is introduced by co-targeting.

To determine the effect of this divergence we formalized this bias into a polynomial equation (Equation 1, depicted in Supp. Note Fig. 4) describing the observed interaction frequency of two co-targeted fragments (O_{AB}) within all interactions containing "A".

$$O_{AB} = \frac{E_A \times f_{AB} + (1 - E_A) \times E_B \times f_{AB}}{E_A + (1 - E_A) \times E_B \times f_{AB}} \qquad \text{(Equation 1)}$$

For the numerator, the efficiency of targeting "A" (E_A) and the real frequency of "A-B" (f_{AB}) determines the number of interactions sampled by the A probe and the number of "lost interactions" available for the B probe (1- E_A). These lost interactions are then recovered at the efficiency of the B probe (E_B). The denominator, which describes the total observations involving A, can be simply denoted as E_A plus the number of recovered events (E_B x f_{AB} x [1- E_A]). The level of bias can then be calculated as O_{AB} divided by f_{AB} (Equation 2).

$$Bias = \frac{O_{AB}}{f_{AB}} \qquad \text{(Equation 2)}$$

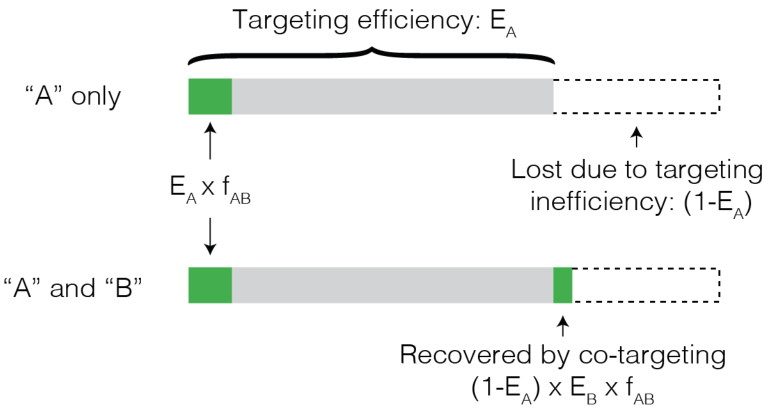

Supp. Note Fig. 4 I Model for the effect on observed frequency caused by co-targeting. a, Diagram of the total number of interactions containing A (entire bar), which includes A-B (green) and the effect of incompletely efficient targeting. Un-enriched (or lost) interactions are in dotted lines, but can be recovered by capture with additional probes.

We used this equation to model the effects of variable efficiency of enrichment (0.05-1.0), and variable underlying interaction frequencies (1/200,000, 1/25,000 and 1/40; based on the min, median, and max interactions frequencies associated with *Hba-1* capture). Under these tested parameters the highest level of bias was a ~20-fold increase in frequency (Supp. Note Fig. 5), seen when the primary target had a low enrichment efficiency, and the secondary target had a high enrichment efficiency. Notably, for any given level of enrichment efficiency the level of bias was variable across the interaction frequencies, with infrequent interactions more affected then frequent interactions.

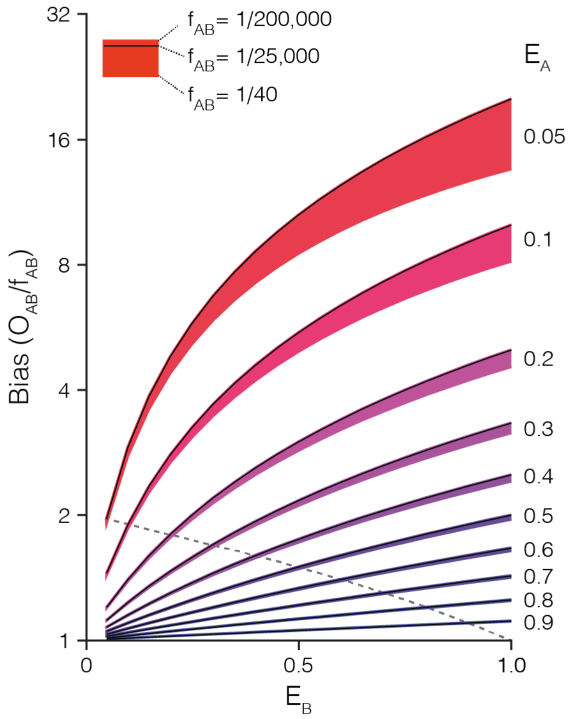

Supp. Note Fig. 5 I Levels of observed frequency bias caused by co-targeting. Variable levels of bias are observed in the observed frequency of A-B interaction (O_{AB}) when altering the efficiency of targeting fragment A (E_A), targeting fragment B (E_B), and the real frequency of A-B interaction (f_{AB}). The Dashed line shows when efficiency of targeting A and B is equal (E_A = E_B). Note that bias is avoided only when E_A is equal to either zero or one.

The implication of these results are quite striking and two-fold. Firstly, when investigating a viewpoint with very low enrichment (say ChIA-PET for a poorly bound PolII site, or Hi-ChIP at a weak H3K27ac peak) then significant enrichment bias is likely to be seen at strong PolII or H3K27ac sites, regardless of whether or not they are actually interacting. In fact, the rarer an interaction is, the stronger the bias effect. Secondly, because all three parameters (enrichment at targets, and the underlying interaction frequency) contribute significantly to the observed bias, proper data correction depends upon having accurate values for all three parameters.

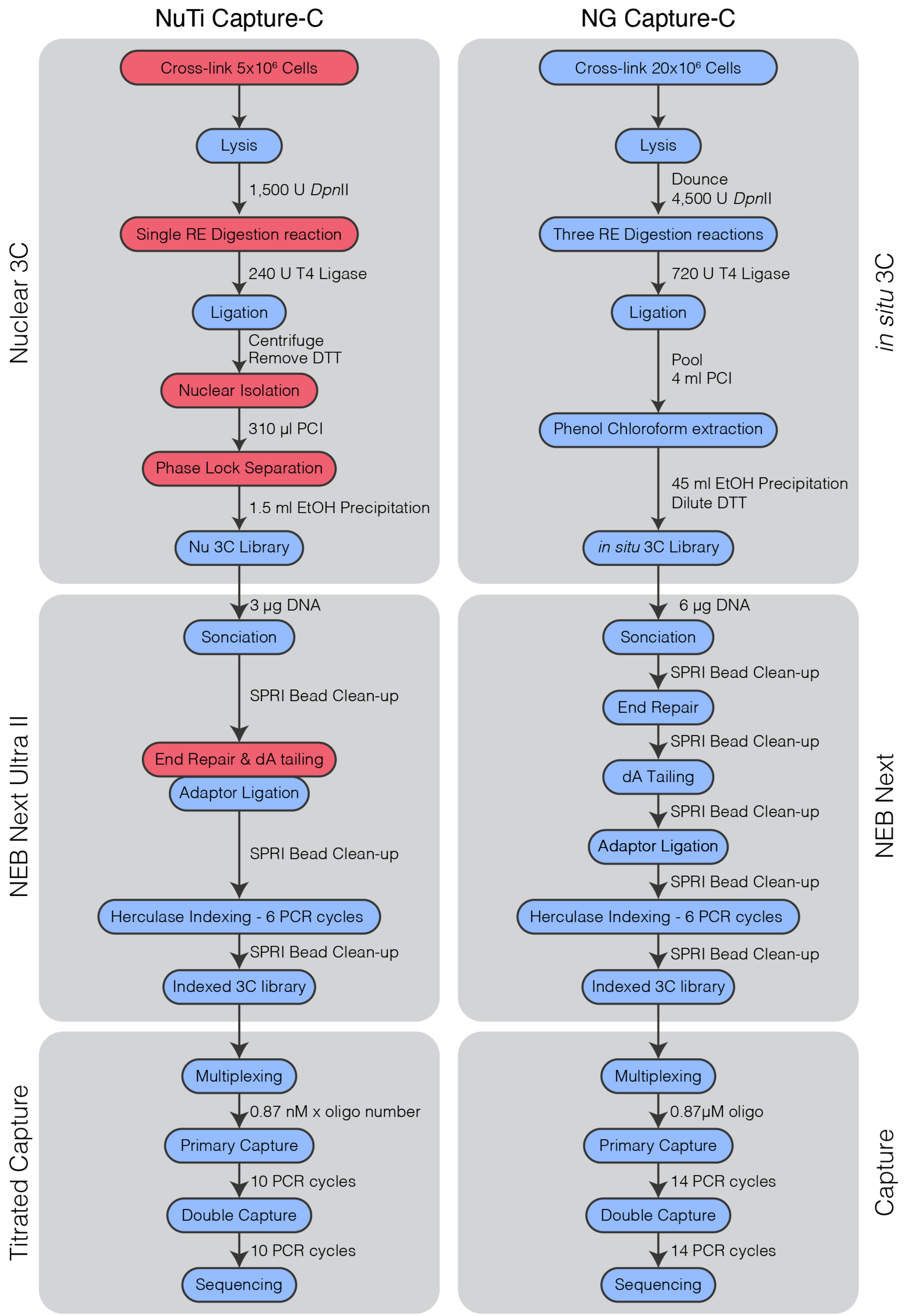

Supp. Fig. 1 I Capture-C workflows. Comparison of experimental workflows for Nuclear-Titratred (NuTi) and Next Generation (NG) Capture-C. Main steps are in blue bubbles, with key innovations for NuTi Capture-C highlighted by red bubbles. Differences in reagents and PCR cycles are shown at individual steps. DTT: Dithiothreitol, PCI: Phenol-Chloroform Isoamyl-alcohol, EtOH: ethanol, SPRI: solid phase reversible immobilisation.

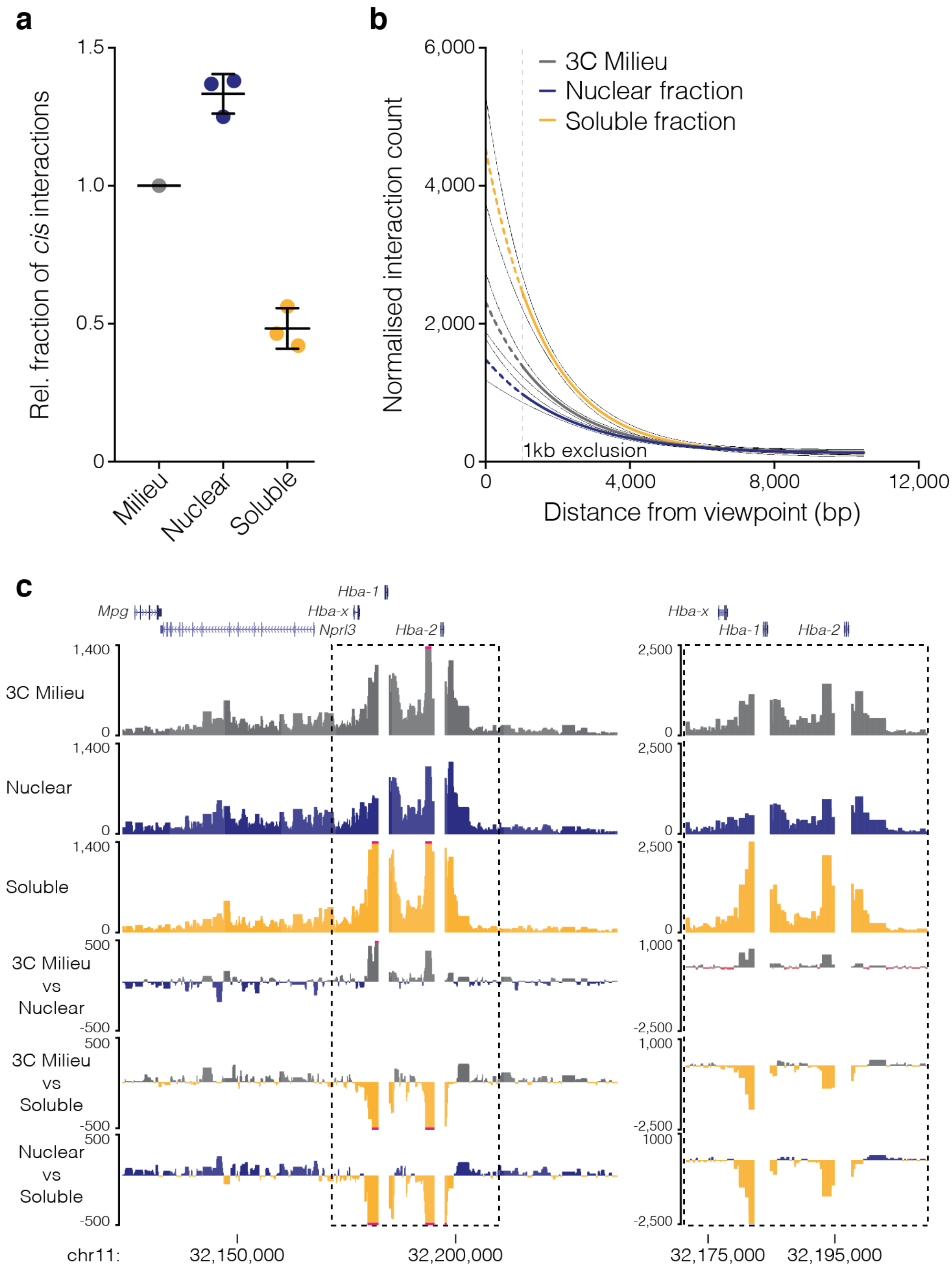

Supp. Fig. 2 | Soluble 3C material has a higher proximity signal. a, Relative fraction of *cis* interactions for libraries generated simultaneously using *in situ*-3C with and without fractionation. Bars show mean and one standard deviation. n=3 independent experiments. **b,** Average number of interactions within 10.5 kb of the *Hba-1/2*, *Hbb-b1/2* and *Slc25a37* capture viewpoints from *in situ*-3C fractions. **c,** Capture profiles and comparison tracks for *Hba-1/2* capture in mouse erythroid cells from total 3C library Milieu or its fractionated nuclear and soluble fractions shows soluble material has a higher proximity signal, likely from small diffusing chunks of digested crosslinked chromatin.

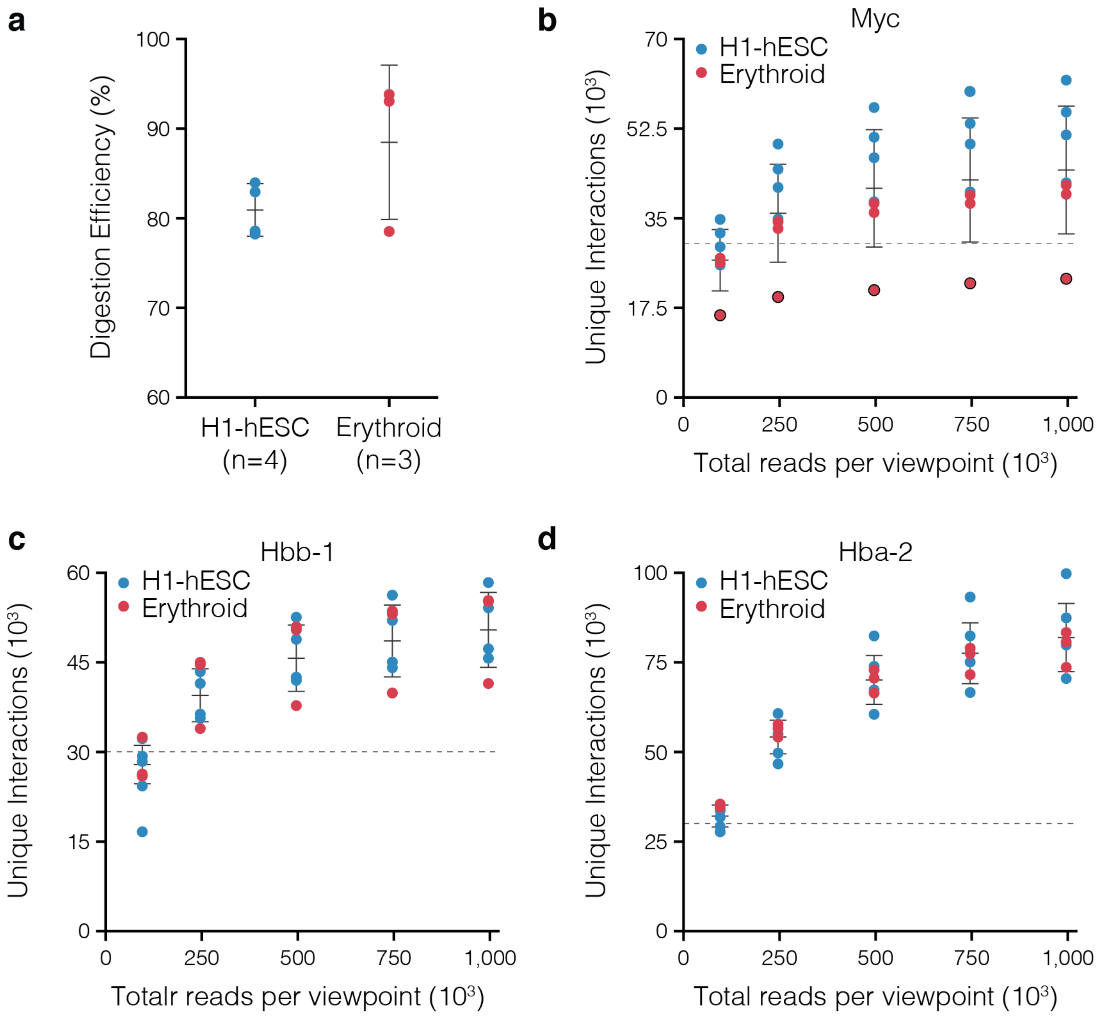

Supp. Fig. 3 | Reporter sensitivity through sequencing depth. a, Digestion efficiency for Nu-3C libraries from human embryonic stem cells (H1-hESC, n=4 independent experiments) and erythroid cells (n=3 n=4 independent experiments) Bars show mean and one standard deviation. NuTi Capture-C was performed for the seven multiplexed libraries targeting *Myc* **(b)**, *Hbb-b1/2* **(c)**, and *Hba-1/2* **(d)** and sequenced to over 10^6 reads per viewpoint per library. Sequence files were subsampled and analyzed to determine number of unique reporters. Dashed lines represent 30,000 unique reporters, or high-sensitivity capture (n=7 independent experiments across the two cell types). For *Myc*, one donor has a polymorphism that removes one of the two *Dpn*II sites on the targeted fragment (black outline) – illustrating the effect of using a single probe per viewpoint. For all panels bars show mean and one standard deviation.

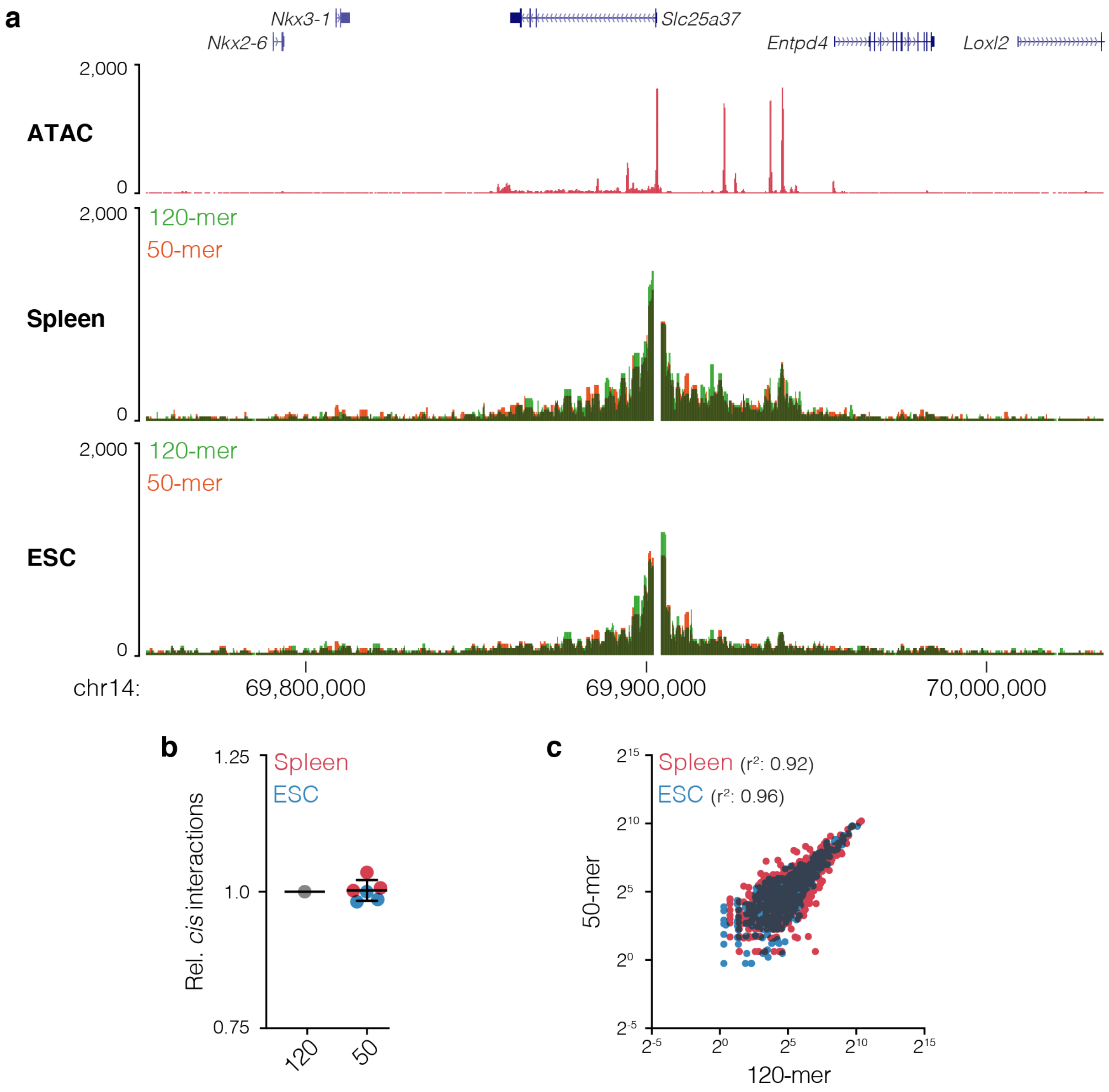

Supp. Fig. 4 I Capture of *Slc25a37* with short probes. a, Overlaid 3C interaction profile for *Slc25a37*, which encodes mitoferrin, from mouse erythroid (n=3 independent experiments) and embryonic stem cells (ESC, n=3 independent experiments) captured with either 120-mer or 50-mer probes. Darkened areas show overlapping signals. **b**, Number of cis reporters relative to 120-mer capture. Bars show mean and one standard deviation. n=6 independent experiments **c**, Comparison of interactions counts from using long or short probes for fragments displayed in panel **a** with Pearson's correlation.

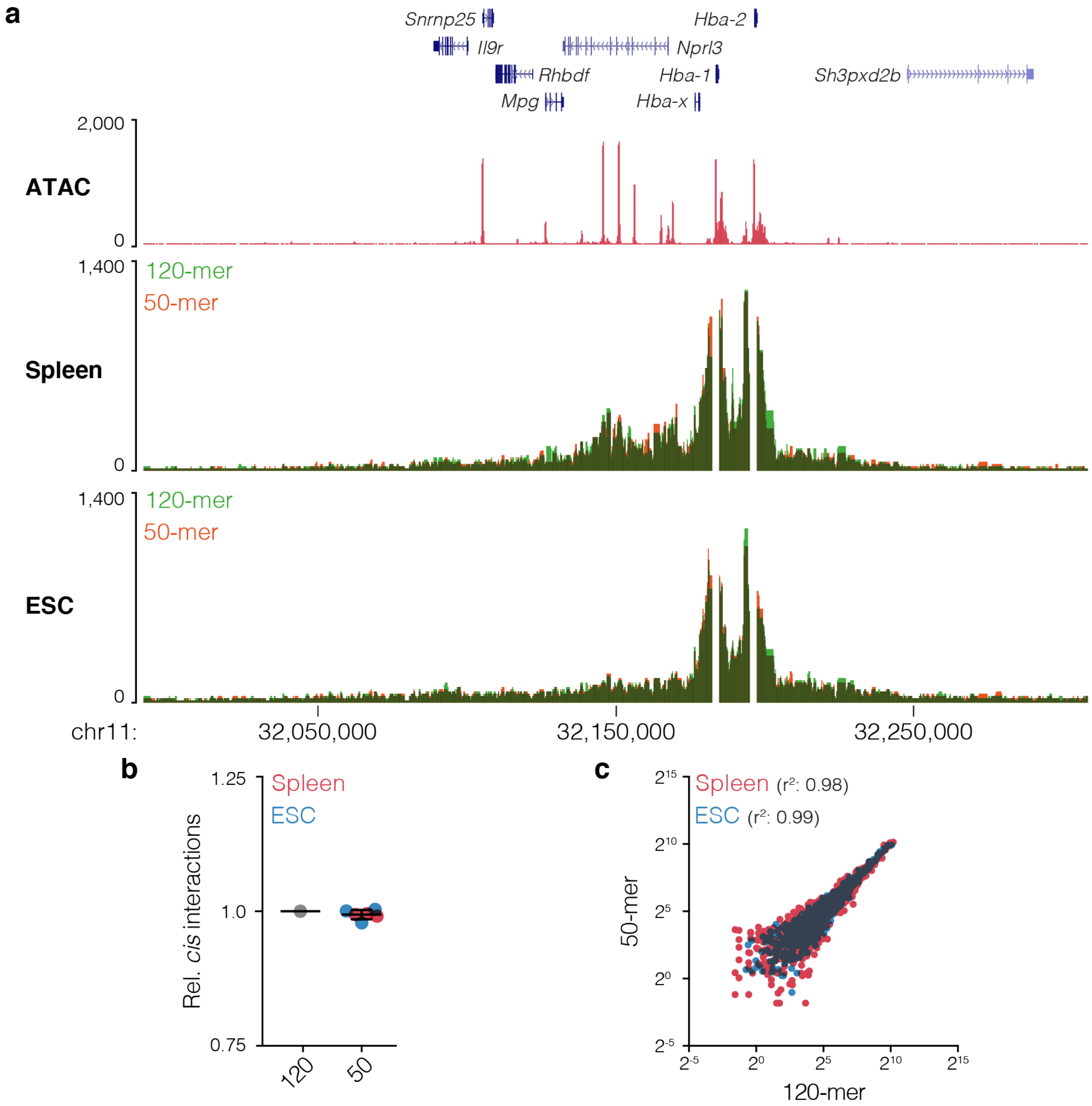

Supp. Fig. 5 | Capture of α-globin locus with short probes. a, Overlaid 3C interaction profile for *Hba-1 and Hba-2*, which encode α-globin, from mouse erythroid (n=3 independent experiments) and embryonic stem cells (ESC, n=3 independent experiments) captured with either 120-mer or 50-mer probes. Darkened areas show overlapping signals. **b**, Number of cis reporters relative to 120-mer capture. Bars show mean and one standard deviation. n=6 independent experiments **c**, Comparison of interactions counts from using long or short probes for fragments displayed in panel **a** with Pearson's correlation.

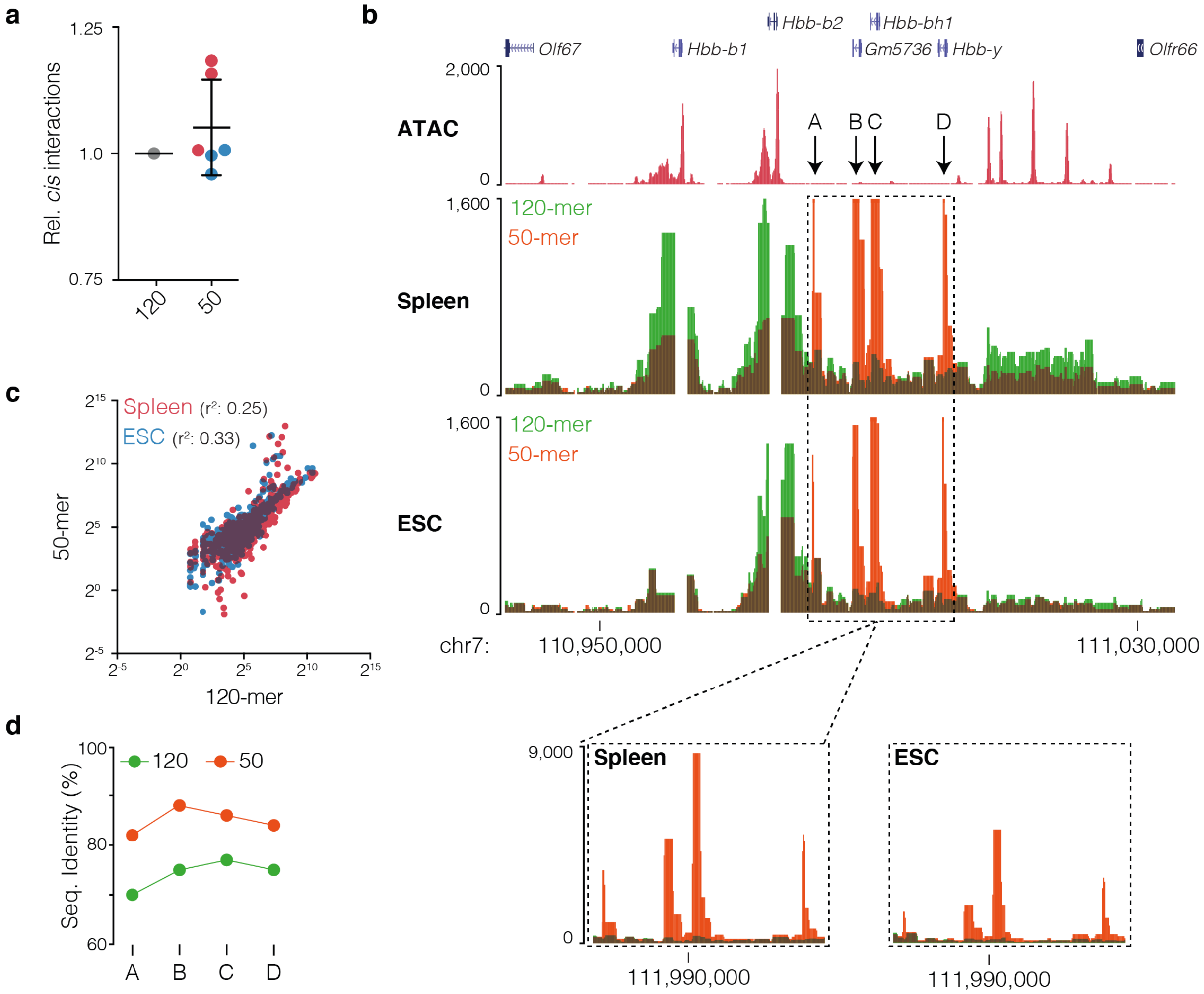

Supp. Fig. 6 l Capture of β-globin locus with short probes. a, Number of *cis* reporters relative to 120-mer capture, bars show mean and standard deviation. n=6 independent experiments. **b**. Overlaid *cis*-normalized 3C interaction profile for *Hbb-b1 and Hbb-b2*, which encode β-globin, from mouse erythroid (n=3) and embryonic stem cells (ESC, n=3) captured with either 120-mer or 50-mer probes. Darkened areas show overlapping signals. ATAC-seq is for erythroid cells. **c**, Comparison of interactions counts using long or short probes with Pearson's correlation. **d,** Clustalω determined sequence identity for 120-mer and 50-mer *Hbb-b1* probe with the four novel peaks of interactions (A-D) seen with the 50-mer probes.

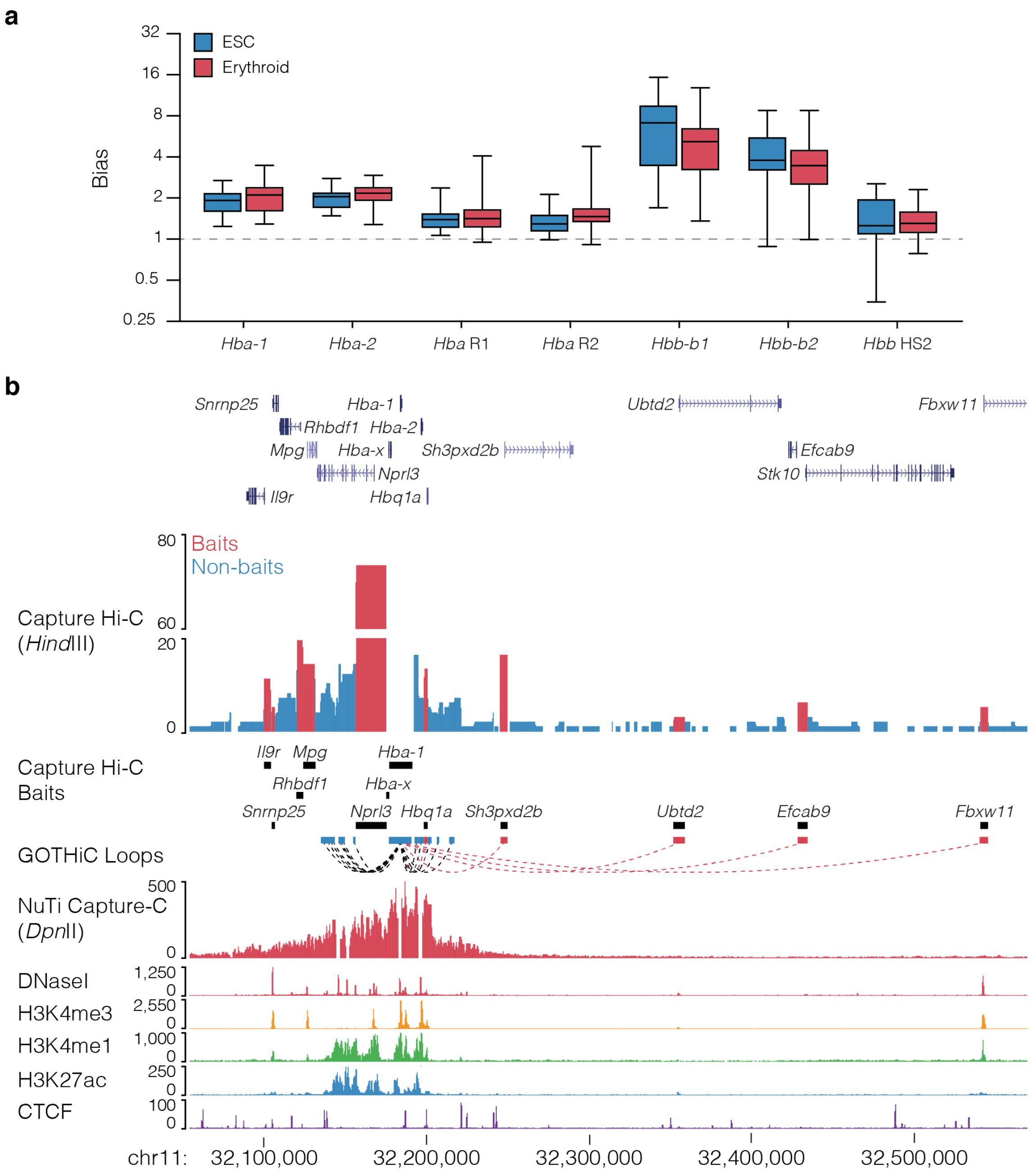

Supp. Fig. 7 I Co-targeting bias observed in Capture-C and Capture Hi-C. a, Per viewpoint levels of bias at co-targeted viewpoints around the α-globin locus (*Hba-1* and *Hba-2* promoters, R1 and R2 enhancers) and the β-globin locus (*Hbb-b1* and *Hbb-b2* promoters, HS2 enhancer). Levels of bias varies across viewpoints and co-targeted fragments but not between erythroid and embryonic stem cells (ESC) indicating bias is primarily caused through the identity of the targeted fragment rather than by cell type signal. Box plots show minima, 25th percentile, median, 75th percentile and maxima. n=3 independent experiments. **b,** Comparison of the 3C interaction profiles for *Hba-1* generated with published Capture Hi-C (targeting all promoters) and NuTi Capture-C (targeting specifically *Hba-1/2* and their two main enhancers – excluded from analysis and seen as gaps in the signal). Total interaction counts for CHi-C in erythroid cells are shown from two independent experiments, fragments and reported significant interactions involving co-targeting coloured red. Note that the peaks over reported long-range significant interactions are not present in NuTi Capture-C and occur specifically at co-targeted fragments (and not adjacent fragments). Erythroid tracks show open chromatin (DNaseI), promoters (H3K4me3), active transcription (H3K27ac), enhancers (H3K4me1), and boundaries (CTCF).

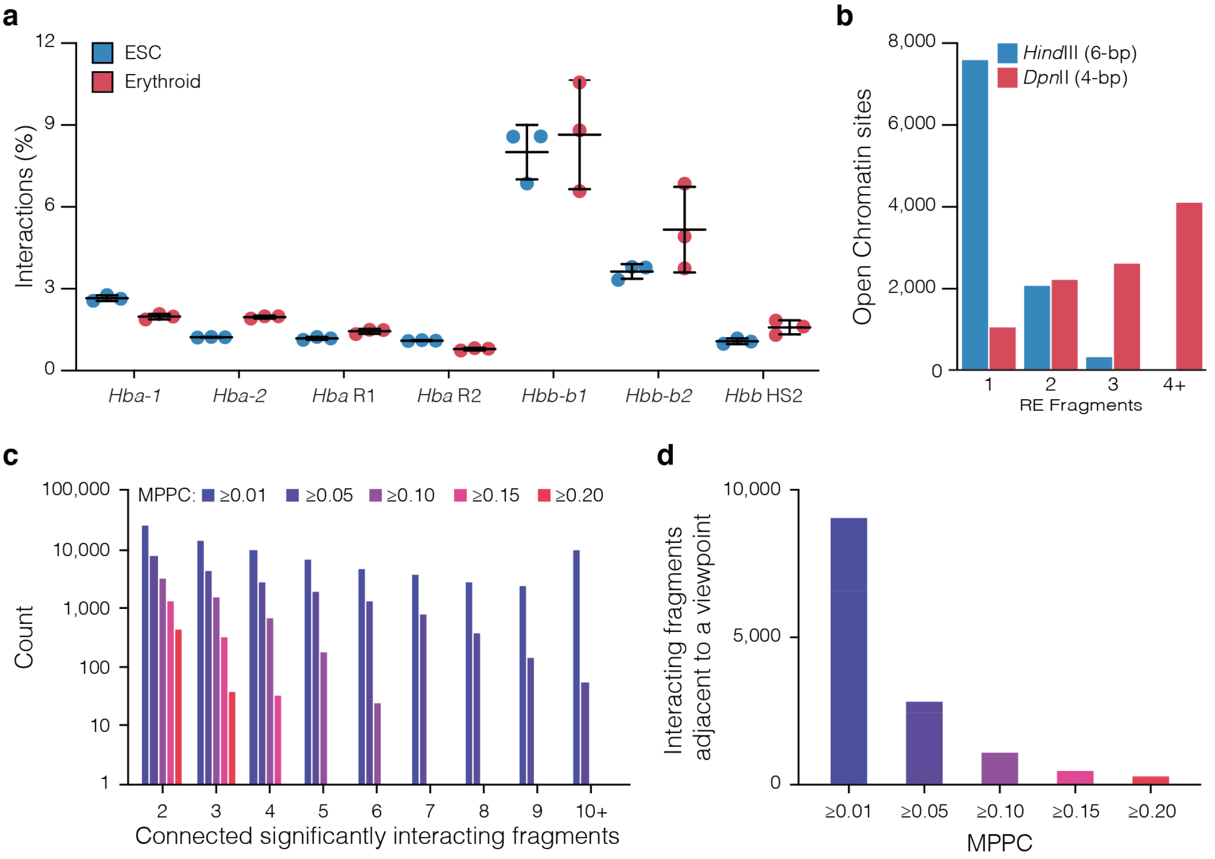

Supp. Fig. 8 I Effect of excluding co-captured fragments from analysis. a, Percentage of total reporter interaction counts found in co-targeted regions. Bars show mean with one standard deviation. n=6 independent experiments. **b,** Number of restriction endonuclease fragments intersecting with annotated erythroid open chromatin sites. **c,** Counts of regions with adjacent significantly interacting fragments. **d,** Count of identified significantly interacting fragments adjacent to a targeted fragment. MPPC: marginal posterior probability of contact.

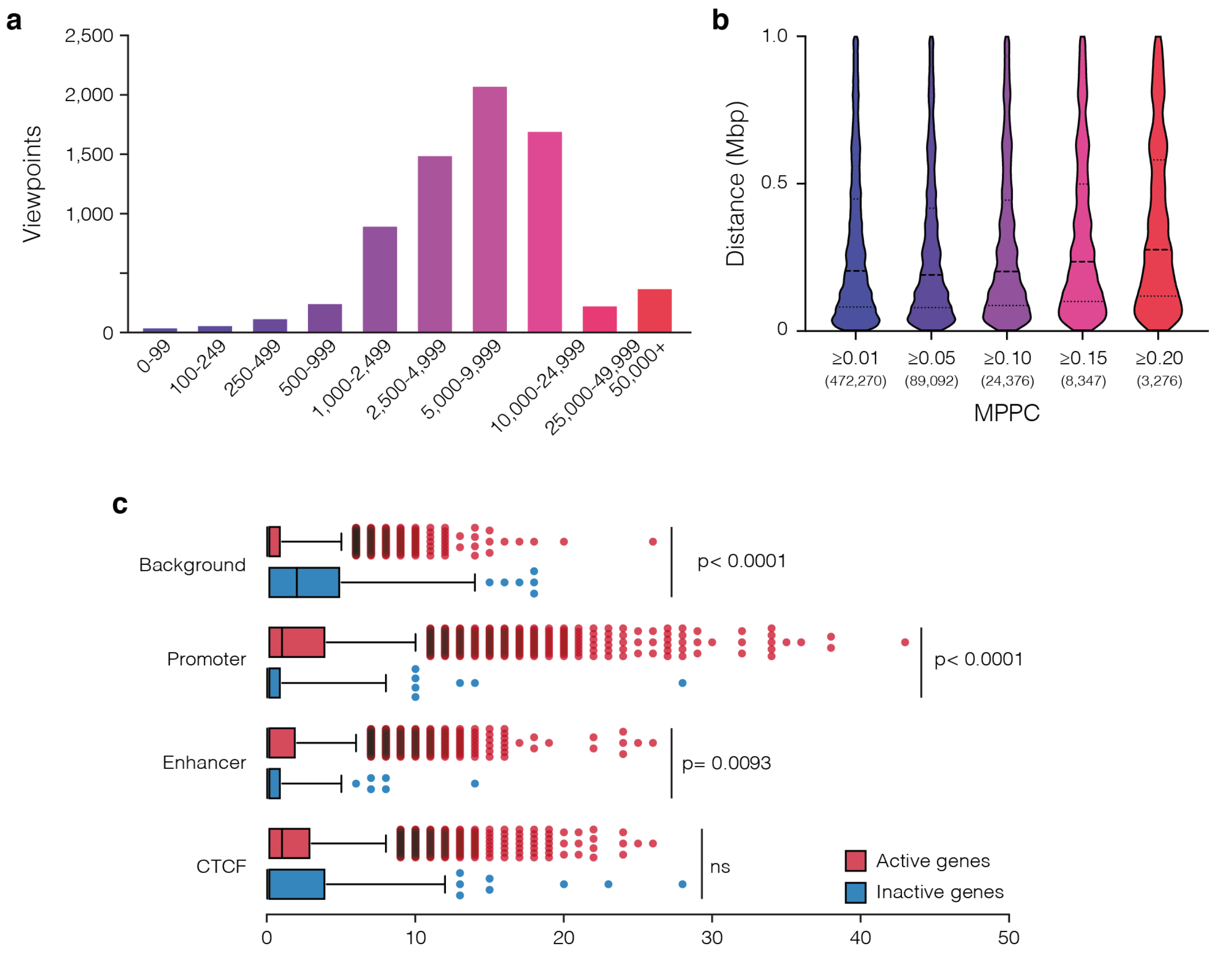

Supp. Fig. 9 l Chromatin signature of captured promoters. a, Histogram of total number of unique reporters identified per viewpoint from triplicate 3C libraries. **b,** Violin plots of the distance between the midpoints of captured promoters and peaky identified interacting fragments with increasing MPPC thresholds. **c,**. Interaction counts for different GenoSTAN classified elements (See Supp. Fig. 12.). Promoter (P_W, P_S and PC), Enhancer (E_W, E_S), CTCF (PC, C_1, C_2). Significant values are for a two-sided Mann-Whitney U tests. Box and Whiskers show: 5th percentile, 25th percentile, median, 75th percentile and 95th percentile.

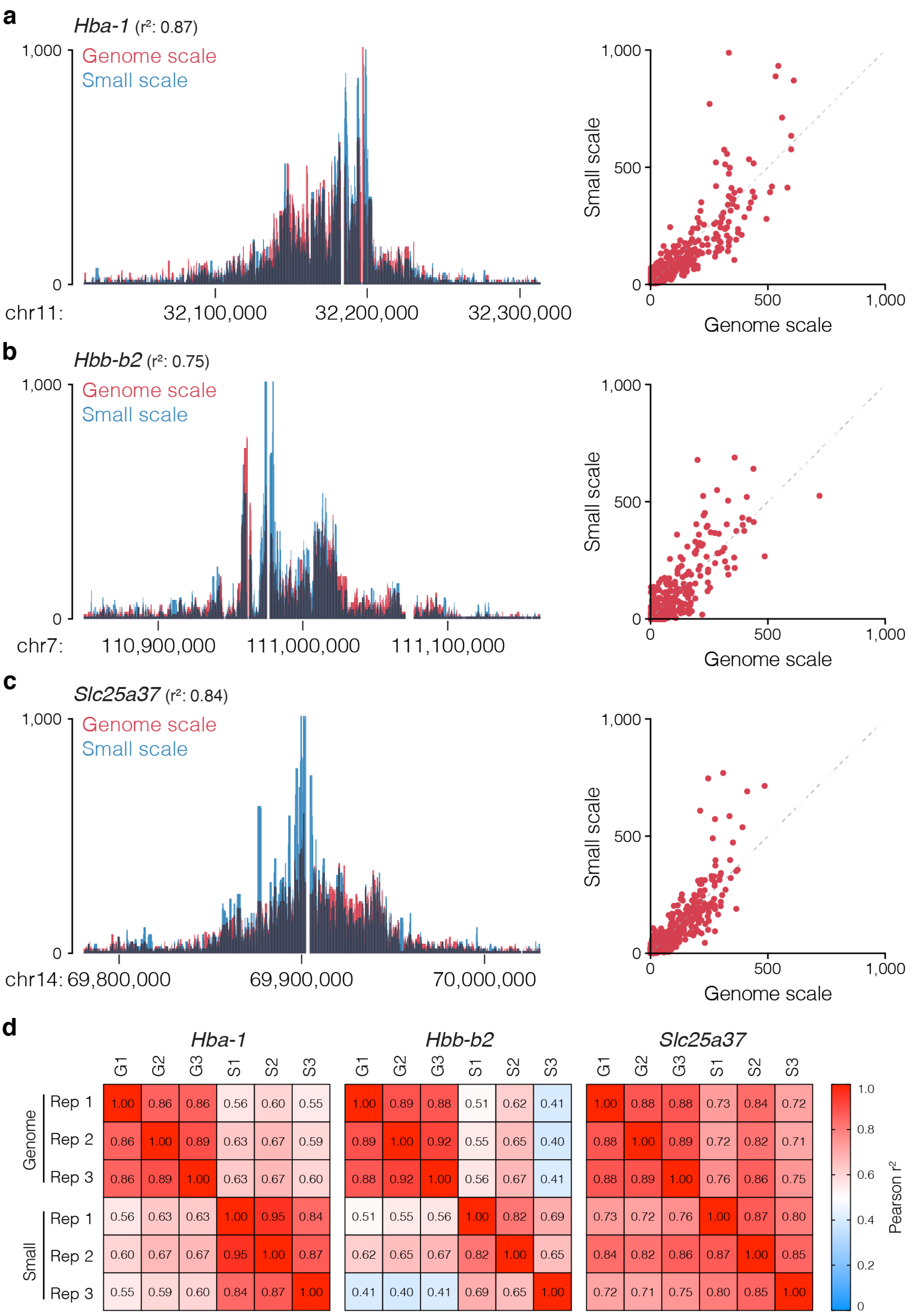

Supp. Fig. 10 I Genome scale capture closely matches designs with fewer probes. Overlaid 3C profiles, Pearson correlation values, and per fragment count correlation plots for the *cis*-normalised mean reporter counts *Hba-1* (**a**), *Hbb-b2* (**b**) and *Slc25a37* (**c**) promoters and replicates (**d**) in mouse erythroid cells when targeting. <10 (small scale) or >7000 (genome scale) viewpoints with NuTi Capture-C. Note overlaid track go dark where signals overlap, seven values >1,000 are not plotted, but were included for correlation analysis.

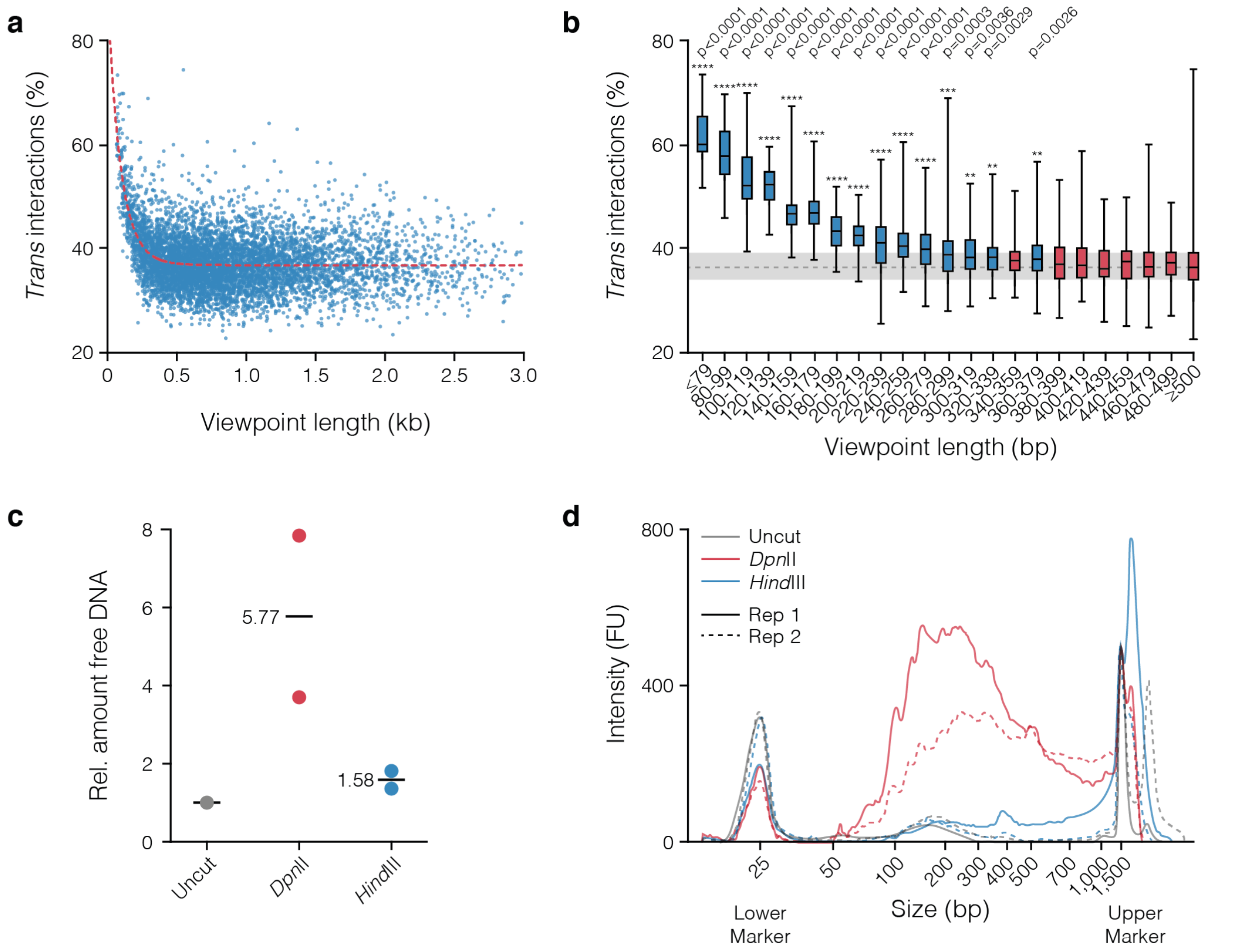

Supp. Fig. 11 | Short fragments have higher levels of *trans* interactions. a, Plot of mean percent of *trans* interactions (n=3) for all viewpoints shorter than 3 kb (n=6,659). Red line shows a non-linear fit to the data (r^2=0.2150, d.f. 19,972). **b,** Box and whisker plot (minima, 25th percentile, median, 75th percentile and maxima) of viewpoints shorter than 500 bp in 20bp bins (n≥12). A two-sided one-way ANOVA was carried out with multiple comparisons for each bin against all viewpoints over 500 bp (n=5,017). Significantly different bins identified by a Dunnett's multiple comparisons test are coloured blue. Relative amount **(c)** and D1000 tapestation profile **(d)** of DNA recovered from the soluble (non-nuclear) fractions of two 3C samples divided across three tubes each and digested overnight with no enzyme (Uncut), a 4-bp cutter (*Dpn*II), and a 6-bp cutter (*Hind*III). FU: Fluorescent units.

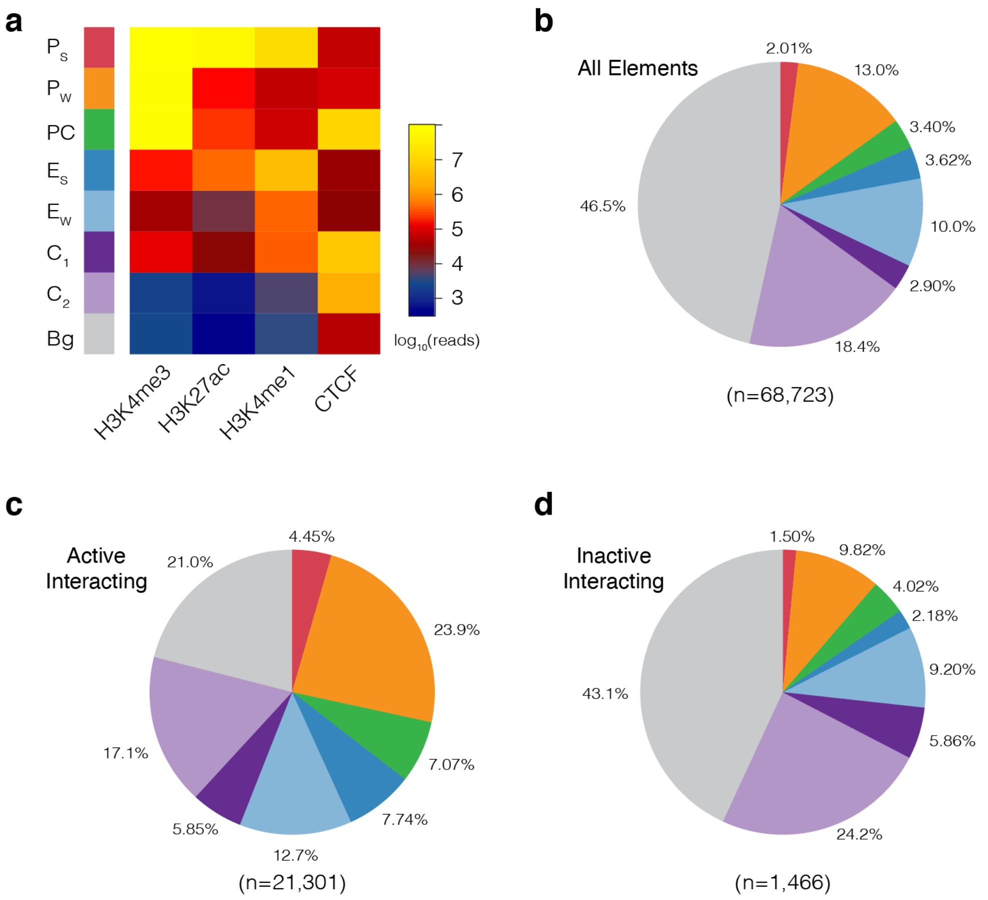

Supp. Fig. 12 | GenoSTAN annotation of the mouse genome in erythroid cells. a, Following curation for similar signal profiles, the GenoSTAN Hidden Markov Model identified eight states for 1 kb erythroid open chromatin regions using ChIP-seq for marks associated with promoters (H3K4me3), active transcription (H3K27ac), enhancers (H3K4me1), and boundaries (CTCF). Identified states were named based on average chromatin profile (shown) as: P_S: Promoter (Strong H3K27ac), P_W: Promoter (Weak H3K27ac), PC: Promoter/CTCF, E_S: Enhancer (Strong H3K27ac), E_W: Enhancer (Weak H3K27ac), C_1: CTCF near promoter/enhancer, C_2: CTCF, Bg: Background. Pie charts showing the proportion of unique annotations for all open chromatin regions **(b)**, fragments significantly interacting with active promoters **(c)**, and fragments significantly interacting with inactive promoters **(d).** Pie chart colours and order match the key in panel a.

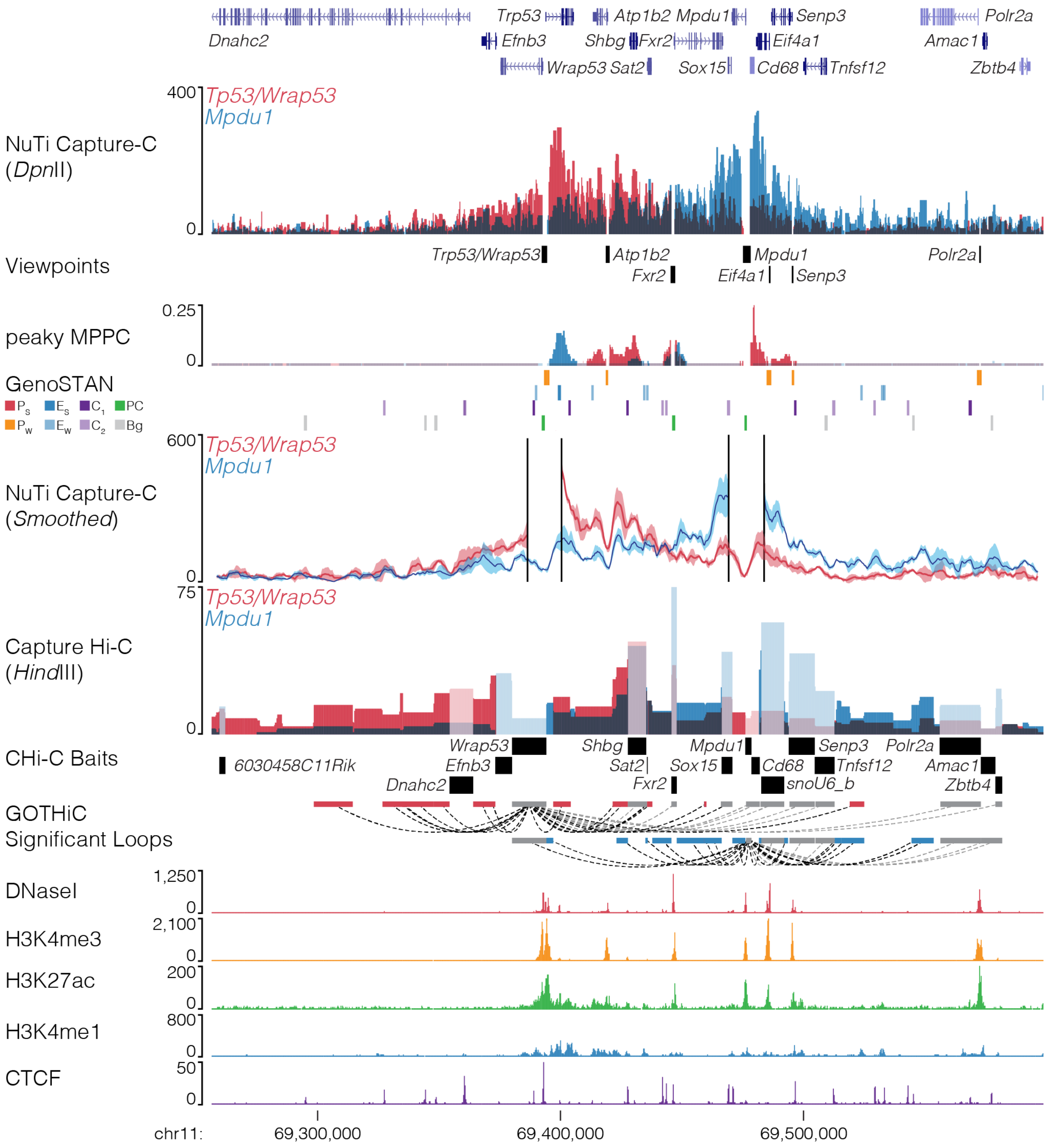

Supp. Fig. 13 I NuTi Capture-C from the *Tp53, Wrap53* and *Mpdu1* promoters. Sequence tracks showing the difference between high-resolution 3C (*Dpn*II, NuTi Capture-C) and low-resolution 3C (*Hind*III, Capture Hi-C) at gene promoters in the same regulatory domain in erythroid cells (mm9, chr11:69,256,536-69,598,480). Tracks in order: UCSC gene annotation, *cis*-normalized mean interactions per *Dpn*II fragment using NuTi Capture-C (n=3 independent 3C libraries), NuTi Capture-C viewpoints, peaky Marginal Posterior Probability of Contact (MPPC) scores with fragments with MPPC ≥0.01 darker, GenoSTAN open chromatin classification, 5 kb windowed mean interactions using NuTi Capture-C (smoothed), total supporting reads per *Hind*III fragment with CHi-C (n=2; co-targeted fragments are lighter in colour), CHi-C bait fragments, loops between reported significantly interacting fragments (co-targeting loops are coloured grey), erythroid tracks for open chromatin (DNaseI), promoters (H3K4me3), active transcription (H3K27ac), enhancers (H3K4me1), and boundaries (CTCF). Note overlapping blue and red signals appear darker in colour (NuTi Capture-C, peaky MPPC, CHi-C).

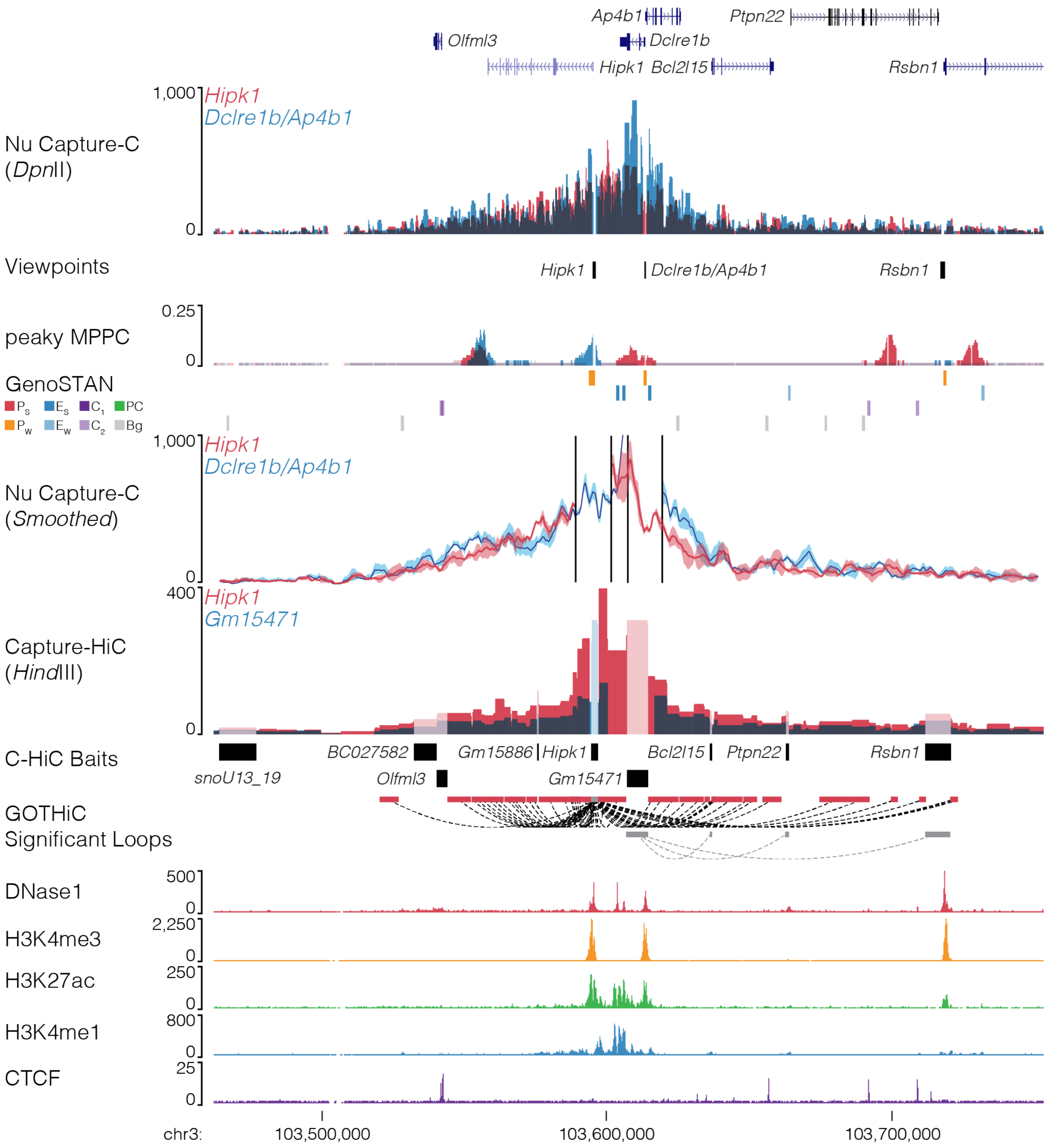

Supp. Fig. 14 l NuTi Capture-C from the *Hipk1, Dclre1b* and *Ap4b1* promoters. Sequence tracks showing the difference between high-resolution 3C (*Dpn*II, NuTi Capture-C) and low-resolution 3C (*Hind*III, Capture Hi-C) at gene promoters in the same regulatory domain in erythroid cells (mm9, chr3:103,462,115-103,753,122). Tracks in order: UCSC gene annotation, *cis*-normalized mean interactions per *Dpn*II fragment using NuTi Capture-C (n=3 independent 3C libraries), NuTi Capture-C viewpoints, peaky Marginal Posterior Probability of Contact (MPPC) scores with fragments with MPPC ≥0.01 darker, GenoSTAN open chromatin classification, 5 kb windowed mean interactions using NuTi Capture-C (smoothed), total supporting reads per *Hind*III fragment with CHi-C (n=2; co-targeted fragments are lighter in colour), CHi-C bait fragments, loops between reported significantly interacting fragments (co-targeting loops are coloured grey), erythroid tracks for open chromatin (DNaseI), promoters (H3K4me3), active transcription (H3K27ac), enhancers (H3K4me1), and boundaries (CTCF). Note overlapping blue and red signals appear darker in colour (NuTi Capture-C, peaky MPPC, CHi-C).

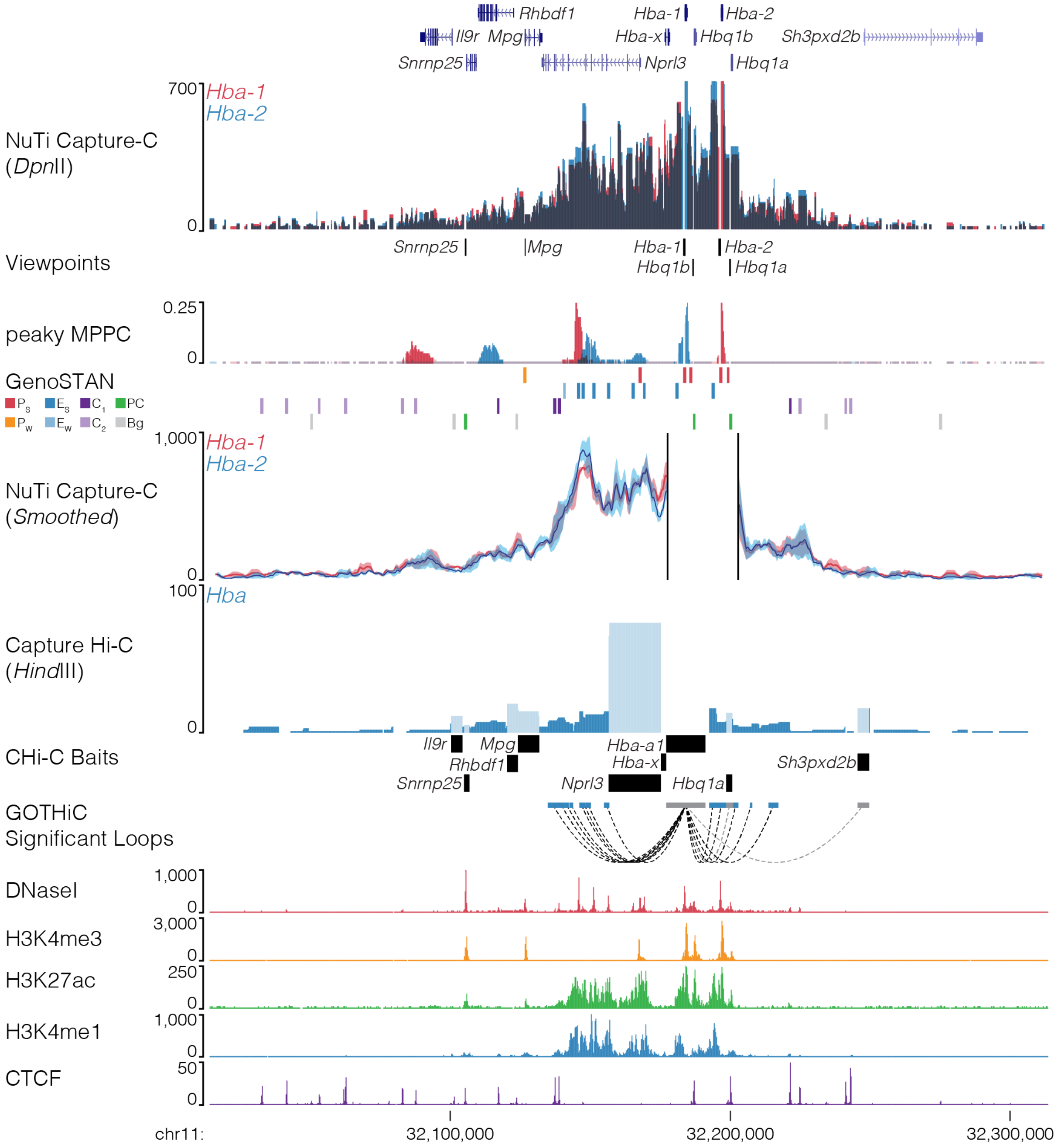

Supp. Fig. 15 I NuTi Capture-C from the *Hba-1* and *Hba-2* promoters. Sequence tracks showing the difference between high-resolution 3C (*Dpn*II, NuTi Capture-C) and low-resolution 3C (*Hind*III, Capture Hi-C) at gene promoters in the same regulatory domain in erythroid cells (mm9, chr3:103,462,115-103,753,122). Tracks in order: UCSC gene annotation, *cis*-normalized mean interactions per *Dpn*II fragment using NuTi Capture-C (n=3 independent 3C libraries), NuTi Capture-C viewpoints, peaky Marginal Posterior Probability of Contact (MPPC) scores with fragments with MPPC ≥0.01 darker, GenoSTAN open chromatin classification, 5 kb windowed mean interactions using NuTi Capture-C (smoothed), total supporting reads per *Hind*III fragment with CHi-C (n=2; co-targeted fragments are lighter in colour), CHi-C bait fragments, loops between reported significantly interacting fragments (co-targeting loops are coloured grey), erythroid tracks for open chromatin (DNaseI), promoters (H3K4me3), active transcription (H3K27ac), enhancers (H3K4me1), and boundaries (CTCF). Note overlapping blue and red signals appear darker in colour (NuTi Capture-C, peaky MPPC, CHi-C).

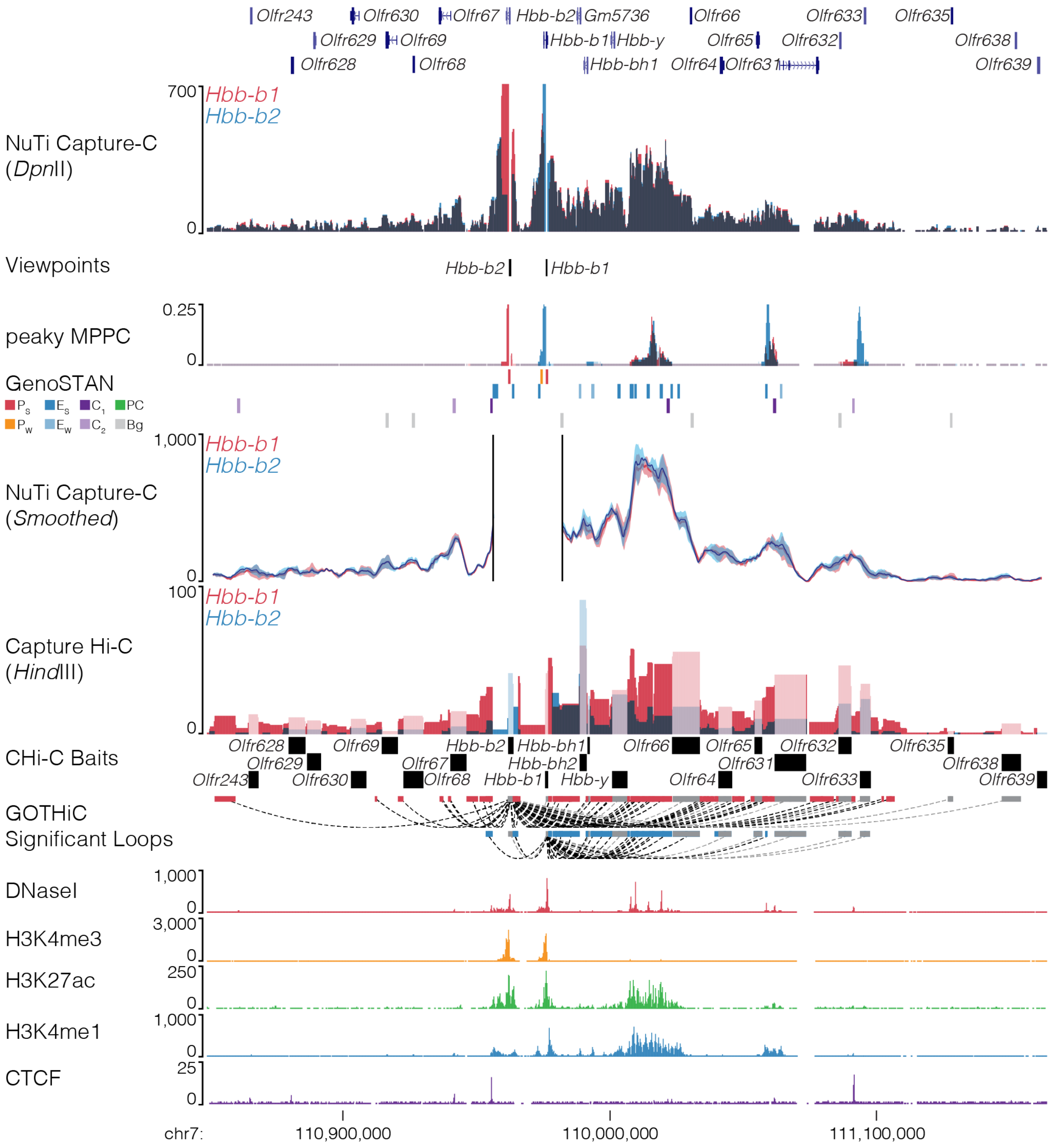

Supp. Fig. 16 I NuTi Capture-C from the *Hbb-b1* and *Hbb-b2* promoters. Sequence tracks showing the difference between high-resolution 3C (*Dpn*II, NuTi Capture-C) and low-resolution 3C (*Hind*III, Capture Hi-C) at gene promoters in the same regulatory domain in erythroid cells (mm9, chr7:110,848,909-111,163,908). Tracks in order: UCSC gene annotation, *cis*-normalized mean interactions per *Dpn*II fragment using NuTi Capture-C (n=3 independent 3C libraries), NuTi Capture-C viewpoints, peaky Marginal Posterior Probability of Contact (MPPC) scores with fragments with MPPC ≥0.01 darker, GenoSTAN open chromatin classification, 5 kb windowed mean interactions using NuTi Capture-C (smoothed), total supporting reads per *Hind*III fragment with CHi-C (n=2; co-targeted fragments are lighter in colour), CHi-C bait fragments, loops between reported significantly interacting fragments (co-targeting loops are coloured grey), erythroid tracks for open chromatin (DNaseI), promoters (H3K4me3), active transcription (H3K27ac), enhancers (H3K4me1), and boundaries (CTCF). Note overlapping blue and red signals appear darker in colour (NuTi Capture-C, peaky MPPC, CHi-C).

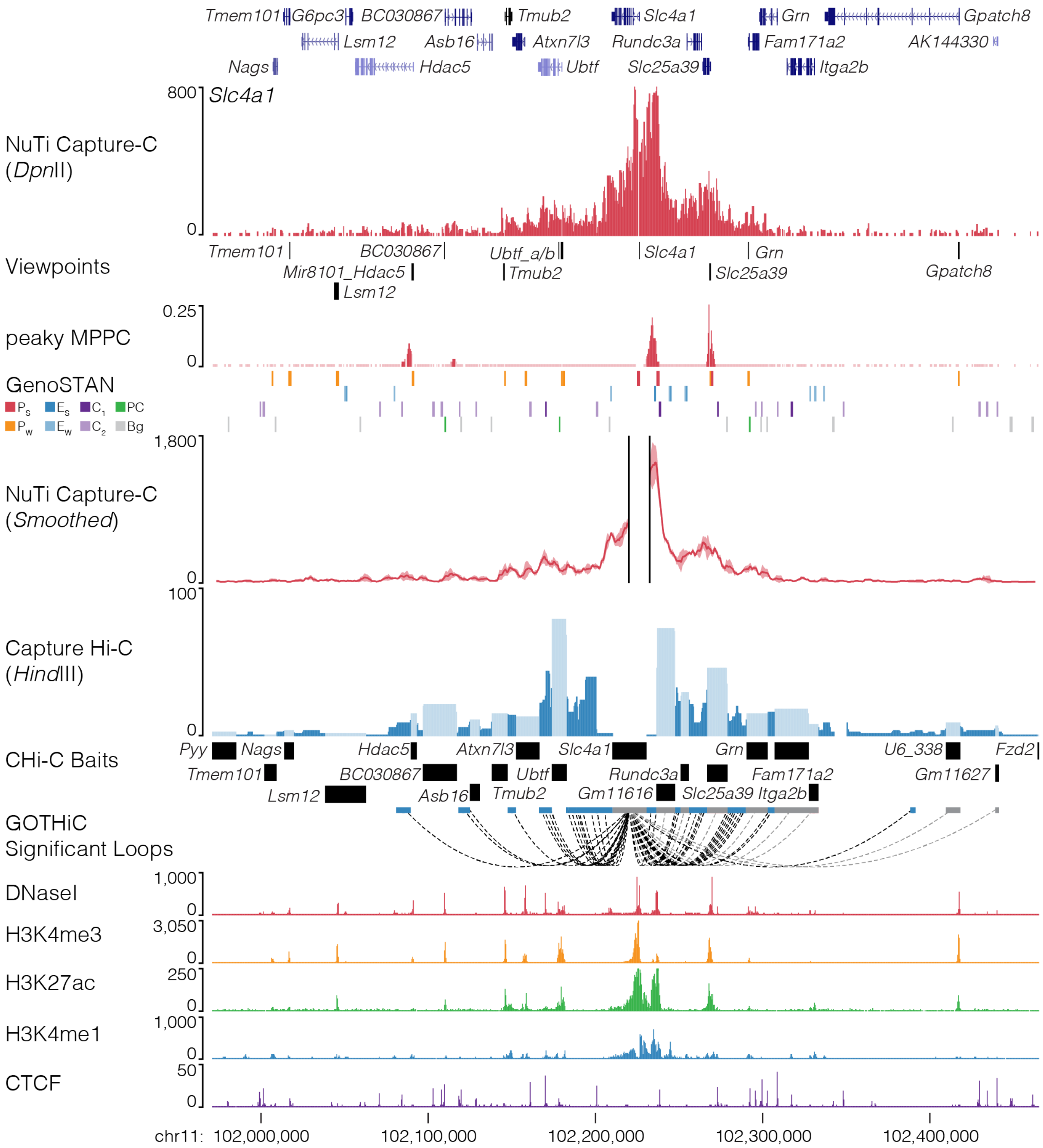

Supp. Fig. 17 I NuTi Capture-C from the *Slc4a1* promoter. Sequence tracks showing the difference between high-resolution 3C (*Dpn*II, NuTi Capture-C) and low-resolution 3C (*Hind*III, Capture Hi-C) at calling interacting fragments (mm9, chr11:101,971,435-102,465,294) in erythroid cells. Tracks in order: UCSC gene annotation, *cis*-normalized mean interactions per *Dpn*II fragment using NuTi Capture-C (n=3 independent 3C libraries), NuTi Capture-C viewpoints, peaky Marginal Posterior Probability of Contact (MPPC) scores with fragments with MPPC ≥0.01 darker, GenoSTAN open chromatin classification, 5 kb windowed mean interactions using NuTi Capture-C (smoothed), total supporting reads per *Hind*III fragment with CHi-C (n=2; co-targeted fragments are lighter in colour), CHi-C bait fragments, loops between reported significantly interacting fragments (co-targeting loops are coloured grey), erythroid tracks for open chromatin (DNaseI), promoters (H3K4me3), active transcription (H3K27ac), enhancers (H3K4me1), and boundaries (CTCF). Note overlapping MPPC signals appear darker in colour.

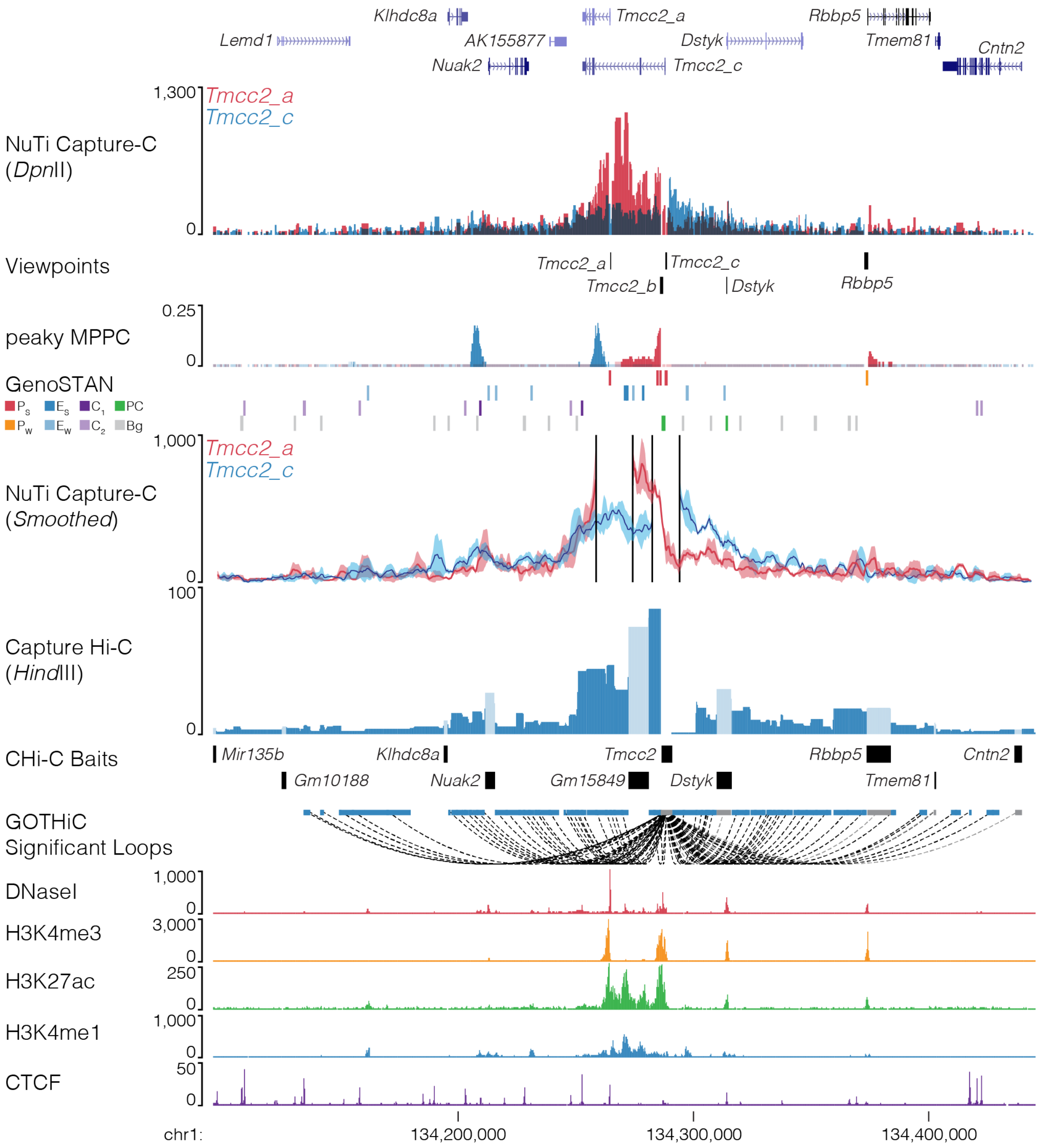

Supp. Fig. 18 I NuTi Capture-C from alternative *Tmcc2* promoters. Sequence tracks showing the difference between high-resolution 3C (*Dpn*II, NuTi Capture-C) and low-resolution 3C (*Hind*III, Capture Hi-C) from alternative *Tmmc2* promoters (mm9, chr1:134,095,540-134,445,539) in erythroid cells. Tracks in order: UCSC gene annotation, *cis*-normalized mean interactions per *Dpn*II fragment using NuTi Capture-C (n=3 independent 3C libraries), NuTi Capture-C viewpoints, peaky Marginal Posterior Probability of Contact (MPPC) scores with fragments with MPPC ≥0.01 darker, GenoSTAN open chromatin classification, 5 kb windowed mean interactions using NuTi Capture-C (smoothed), total supporting reads per *Hind*III fragment with CHi-C (n=2; co-targeted fragments are lighter in colour), CHi-C bait fragments, loops between reported significantly interacting fragments (co-targeting loops are coloured grey), erythroid tracks for open chromatin (DNaseI), promoters (H3K4me3), active transcription (H3K27ac), enhancers (H3K4me1), and boundaries (CTCF). Note overlapping blue and red signals appear darker in colour (NuTi Capture-C, peaky MPPC, CHi-C).

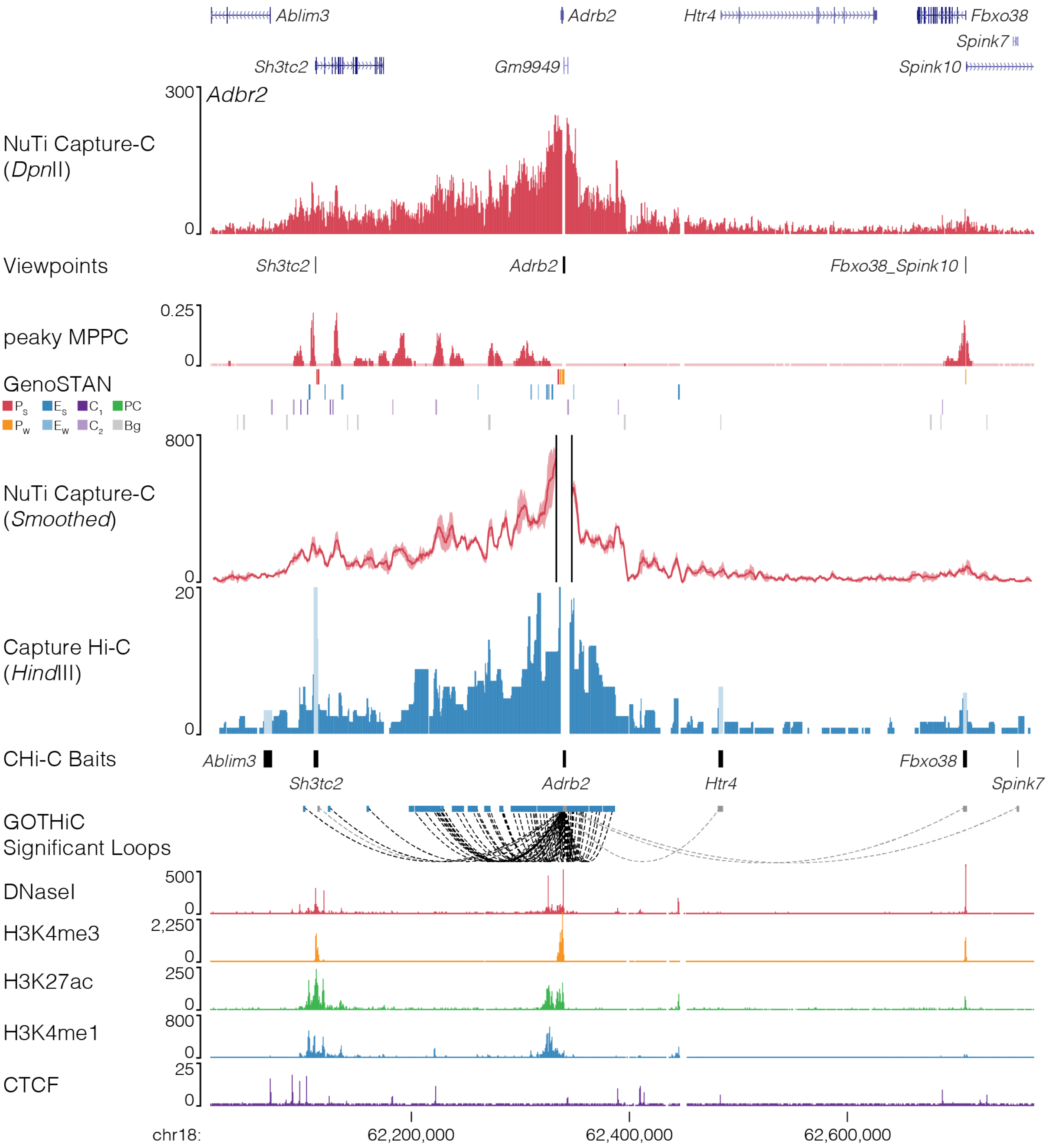

Supp. Fig. 19 I NuTi Capture-C from the *Adrb2* promoter. Sequence tracks showing the difference between high-resolution 3C (*Dpn*II, NuTi Capture-C) and low-resolution 3C (*Hind*III, Capture Hi-C) at calling interacting fragments (mm9, chr18:62,016,212-62,771,180) in erythroid cells. Tracks in order: UCSC gene annotation, *cis*-normalized mean interactions per *Dpn*II fragment using NuTi Capture-C (n=3 independent 3C libraries), NuTi Capture-C viewpoints, peaky Marginal Posterior Probability of Contact (MPPC) scores with fragments with MPPC ≥0.01 darker, GenoSTAN open chromatin classification, 5 kb windowed mean interactions using NuTi Capture-C (smoothed), total supporting reads per *Hind*III fragment with CHi-C (n=2; co-targeted fragments are lighter in colour), CHi-C bait fragments, loops between reported significantly interacting fragments (co-targeting loops are coloured grey), erythroid tracks for open chromatin (DNaseI), promoters (H3K4me3), active transcription (H3K27ac), enhancers (H3K4me1), and boundaries (CTCF). Note overlapping MPPC signals appear darker in colour.

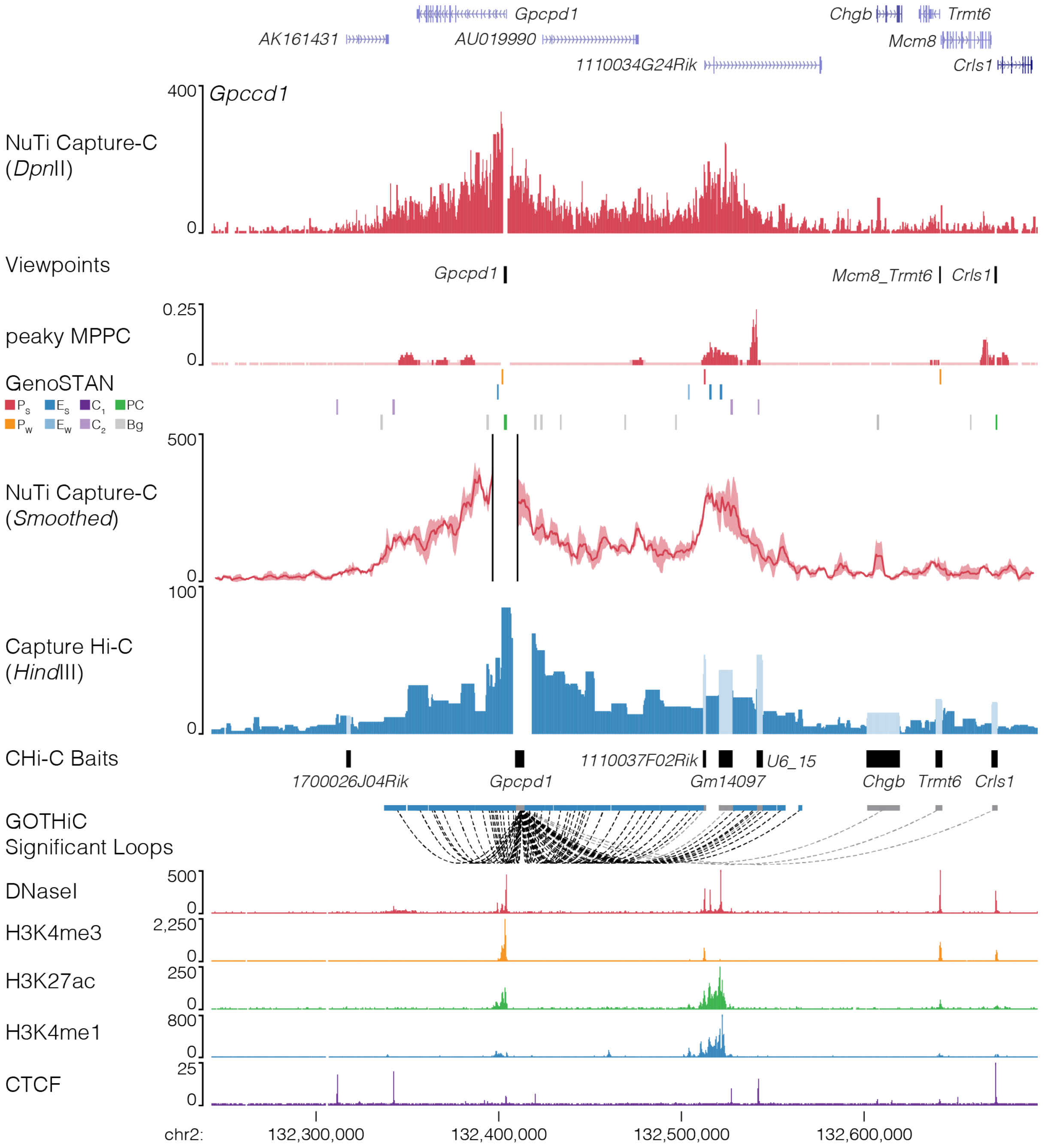

Supp. Fig. 20 | NuTi Capture-C from the *Gpcpd1* promoter. Sequence tracks showing the difference between high-resolution 3C (*Dpn*II, NuTi Capture-C) and low-resolution 3C (*Hind*III, Capture Hi-C) at calling interacting fragments (mm9, chr2:132,242,416-132,695,297) in erythroid cells. Tracks in order: UCSC gene annotation, *cis*-normalized mean interactions per *Dpn*II fragment using NuTi Capture-C (n=3 independent 3C libraries), NuTi Capture-C viewpoints, peaky Marginal Posterior Probability of Contact (MPPC) scores with fragments with MPPC ≥0.01 darker, GenoSTAN open chromatin classification, 5 kb windowed mean interactions using NuTi Capture-C (smoothed), total supporting reads per *Hind*III fragment with CHi-C (n=2; co-targeted fragments are lighter in colour), CHi-C bait fragments, loops between reported significantly interacting fragments (co-targeting loops are coloured grey), erythroid tracks for open chromatin (DNaseI), promoters (H3K4me3), active transcription (H3K27ac), enhancers (H3K4me1), and boundaries (CTCF). Note overlapping MPPC signals appear darker in colour.

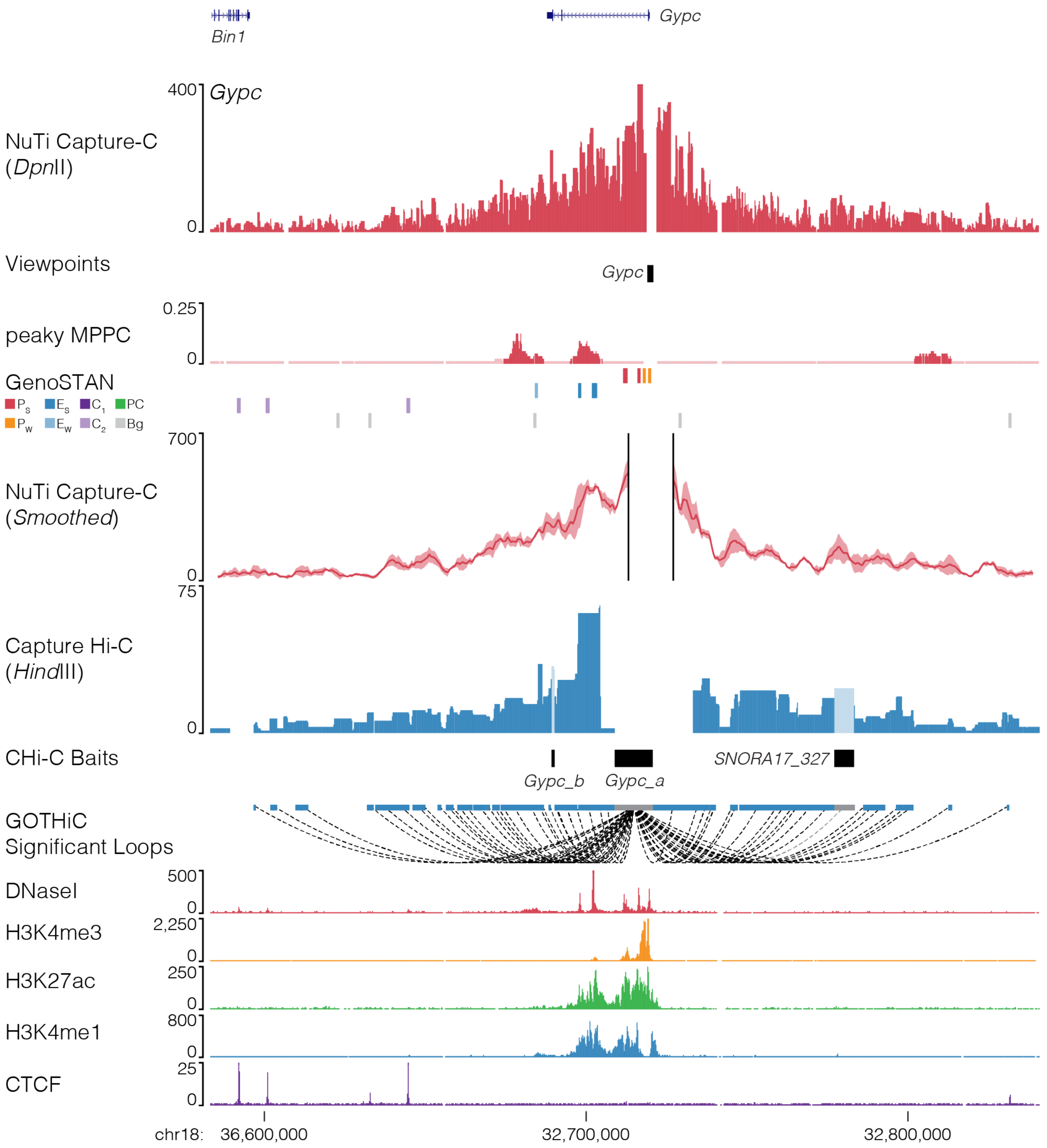

Supp. Fig. 21 | NuTi Capture-C from the *Gypc* promoter. Sequence tracks showing the difference between high-resolution 3C (*Dpn*II, NuTi Capture-C) and low-resolution 3C (*Hind*III, Capture Hi-C) at calling interacting fragments (mm9, chr18:32,583,205-32,841,048) in erythroid cells. Tracks in order: UCSC gene annotation, *cis*-normalized mean interactions per *Dpn*II fragment using NuTi Capture-C (n=3 independent 3C libraries), NuTi Capture-C viewpoints, peaky Marginal Posterior Probability of Contact (MPPC) scores with fragments with MPPC ≥0.01 darker, GenoSTAN open chromatin classification, 5 kb windowed mean interactions using NuTi Capture-C (smoothed), total supporting reads per *Hind*III fragment with CHi-C (n=2; co-targeted fragments are lighter in colour), CHi-C bait fragments, loops between reported significantly interacting fragments (co-targeting loops are coloured grey), erythroid tracks for open chromatin (DNaseI), promoters (H3K4me3), active transcription (H3K27ac), enhancers (H3K4me1), and boundaries (CTCF). Note overlapping MPPC signals appear darker in colour.

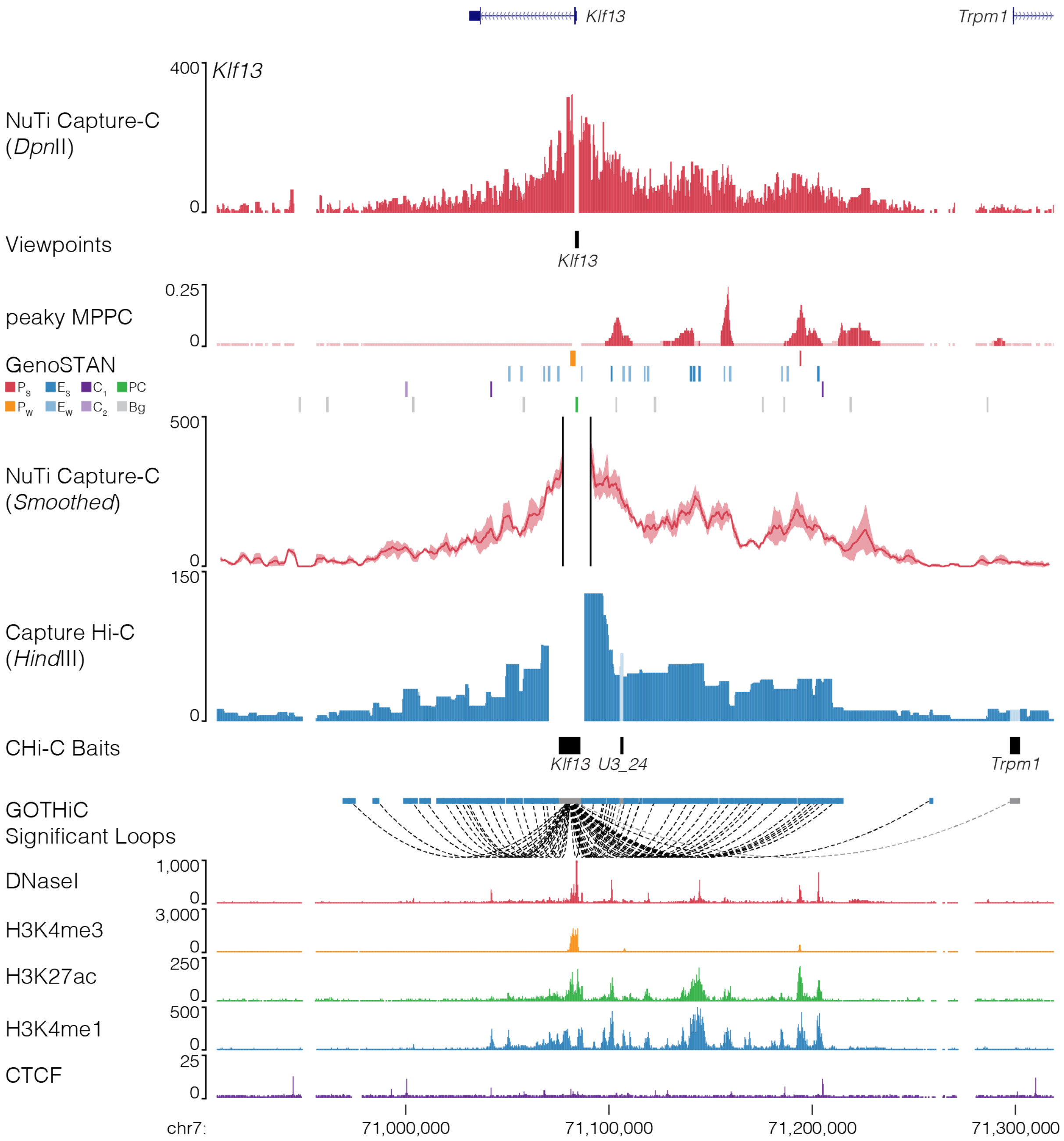

Supp. Fig. 22 | NuTi Capture-C from the *Klf13* promoter. Sequence tracks showing the difference between high-resolution 3C (*Dpn*II, NuTi Capture-C) and low-resolution 3C (*Hind*III, Capture Hi-C) at calling interacting fragments (mm9, chr7:70,906,844-71,318,843) in erythroid cells. Tracks in order: UCSC gene annotation, *cis*-normalized mean interactions per *Dpn*II fragment using NuTi Capture-C (n=3 independent 3C libraries), NuTi Capture-C viewpoints, peaky Marginal Posterior Probability of Contact (MPPC) scores with fragments with MPPC ≥0.01 darker, GenoSTAN open chromatin classification, 5 kb windowed mean interactions using NuTi Capture-C (smoothed), total supporting reads per *Hind*III fragment with CHi-C (n=2; co-targeted fragments are lighter in colour), CHi-C bait fragments, loops between reported significantly interacting fragments (co-targeting loops are coloured grey), erythroid tracks for open chromatin (DNaseI), promoters (H3K4me3), active transcription (H3K27ac), enhancers (H3K4me1), and boundaries (CTCF). Note overlapping MPPC signals appear darker in colour.

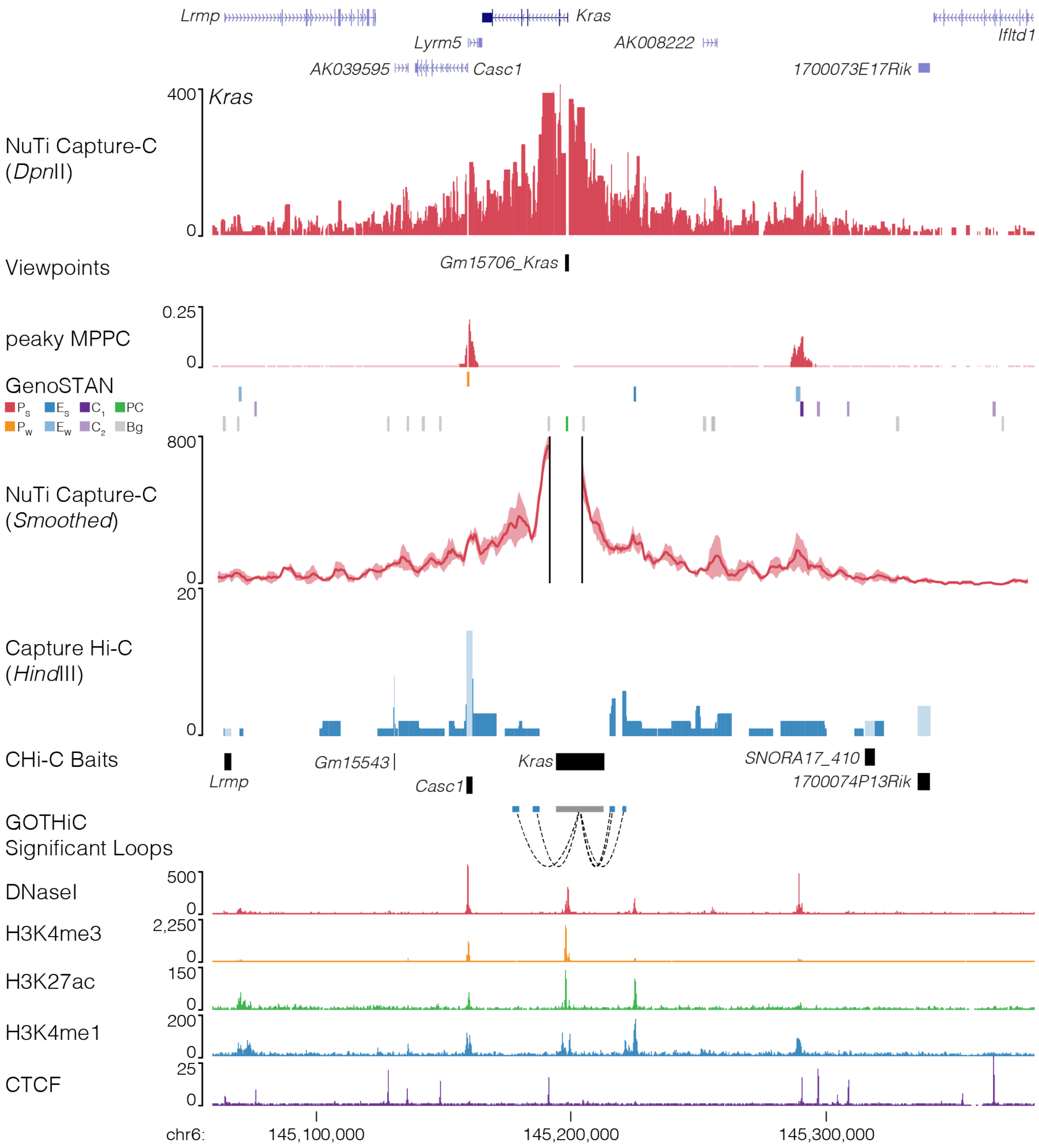

Supp. Fig. 23 | NuTi Capture-C from the *Kras* promoter. Sequence tracks showing the difference between high-resolution 3C (*Dpn*II, NuTi Capture-C) and low-resolution 3C (*Hind*III, Capture Hi-C) at calling interacting fragments (mm9, chr6:145,059,451-145,381,678) in erythroid cells. Tracks in order: UCSC gene annotation, *cis*-normalized mean interactions per *Dpn*II fragment using NuTi Capture-C (n=3 independent 3C libraries), NuTi Capture-C viewpoints, peaky Marginal Posterior Probability of Contact (MPPC) scores with fragments with MPPC ≥0.01 darker, GenoSTAN open chromatin classification, 5 kb windowed mean interactions using NuTi Capture-C (smoothed), total supporting reads per *Hind*III fragment with CHi-C (n=2; co-targeted fragments are lighter in colour), CHi-C bait fragments, loops between reported significantly interacting fragments (co-targeting loops are coloured grey), erythroid tracks for open chromatin (DNaseI), promoters (H3K4me3), active transcription (H3K27ac), enhancers (H3K4me1), and boundaries (CTCF). Note overlapping MPPC signals appear darker in colour.

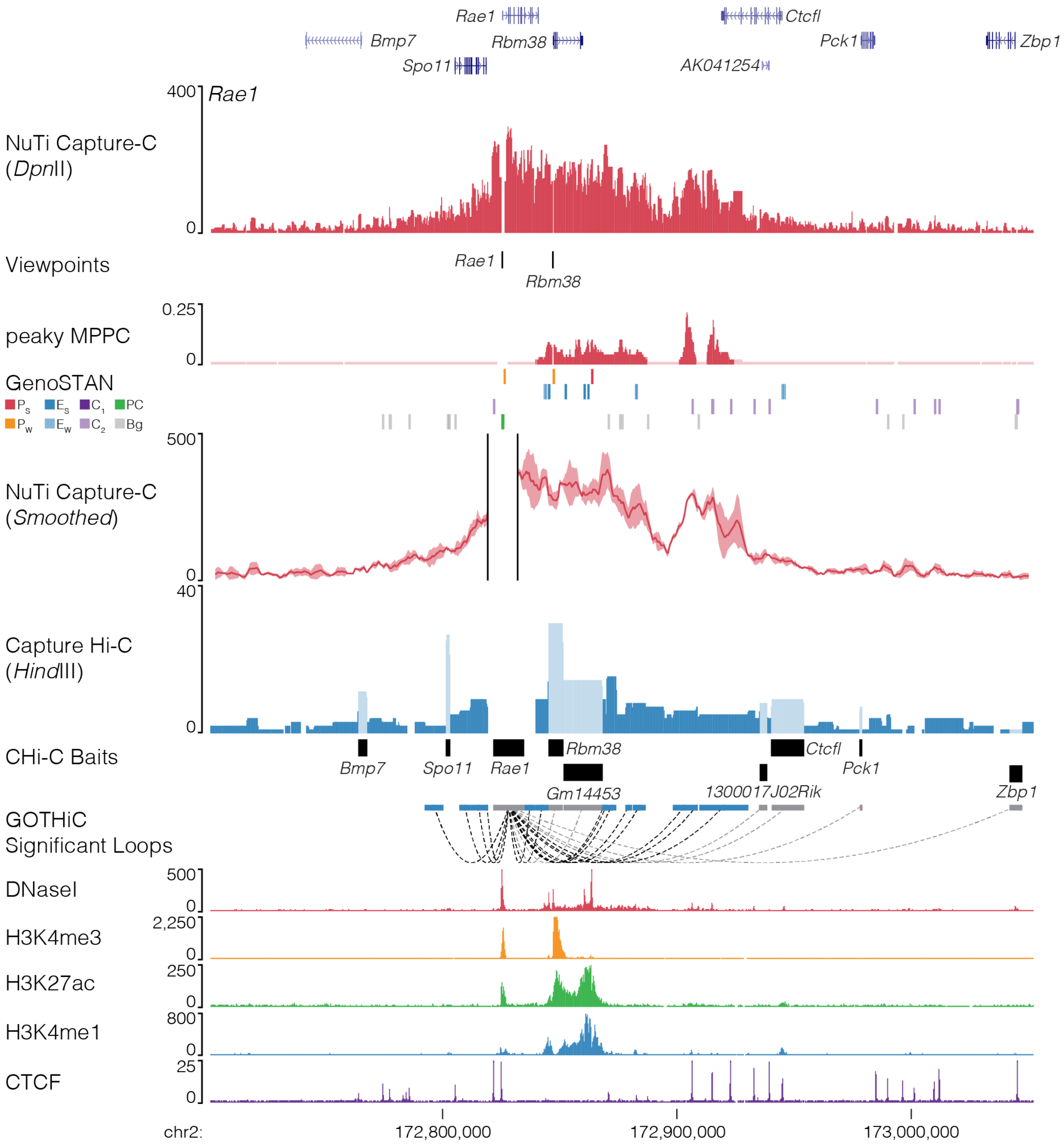

Supp. Fig. 24 I NuTi Capture-C from the *Rae1* promoter. Sequence tracks showing the difference between high-resolution 3C (*Dpn*II, NuTi Capture-C) and low-resolution 3C (*Hind*III, Capture Hi-C) at calling interacting fragments (mm9, chr2:172,701,139-173,052,278) in erythroid cells. Tracks in order: UCSC gene annotation, *cis*-normalized mean interactions per *Dpn*II fragment using NuTi Capture-C (n=3 independent 3C libraries), NuTi Capture-C viewpoints, peaky Marginal Posterior Probability of Contact (MPPC) scores with fragments with MPPC ≥0.01 darker, GenoSTAN open chromatin classification, 5 kb windowed mean interactions using NuTi Capture-C (smoothed), total supporting reads per *Hind*III fragment with CHi-C (n=2; co-targeted fragments are lighter in colour), CHi-C bait fragments, loops between reported significantly interacting fragments (co-targeting loops are coloured grey), erythroid tracks for open chromatin (DNaseI), promoters (H3K4me3), active transcription (H3K27ac), enhancers (H3K4me1), and boundaries (CTCF). Note overlapping MPPC signals appear darker in colour.

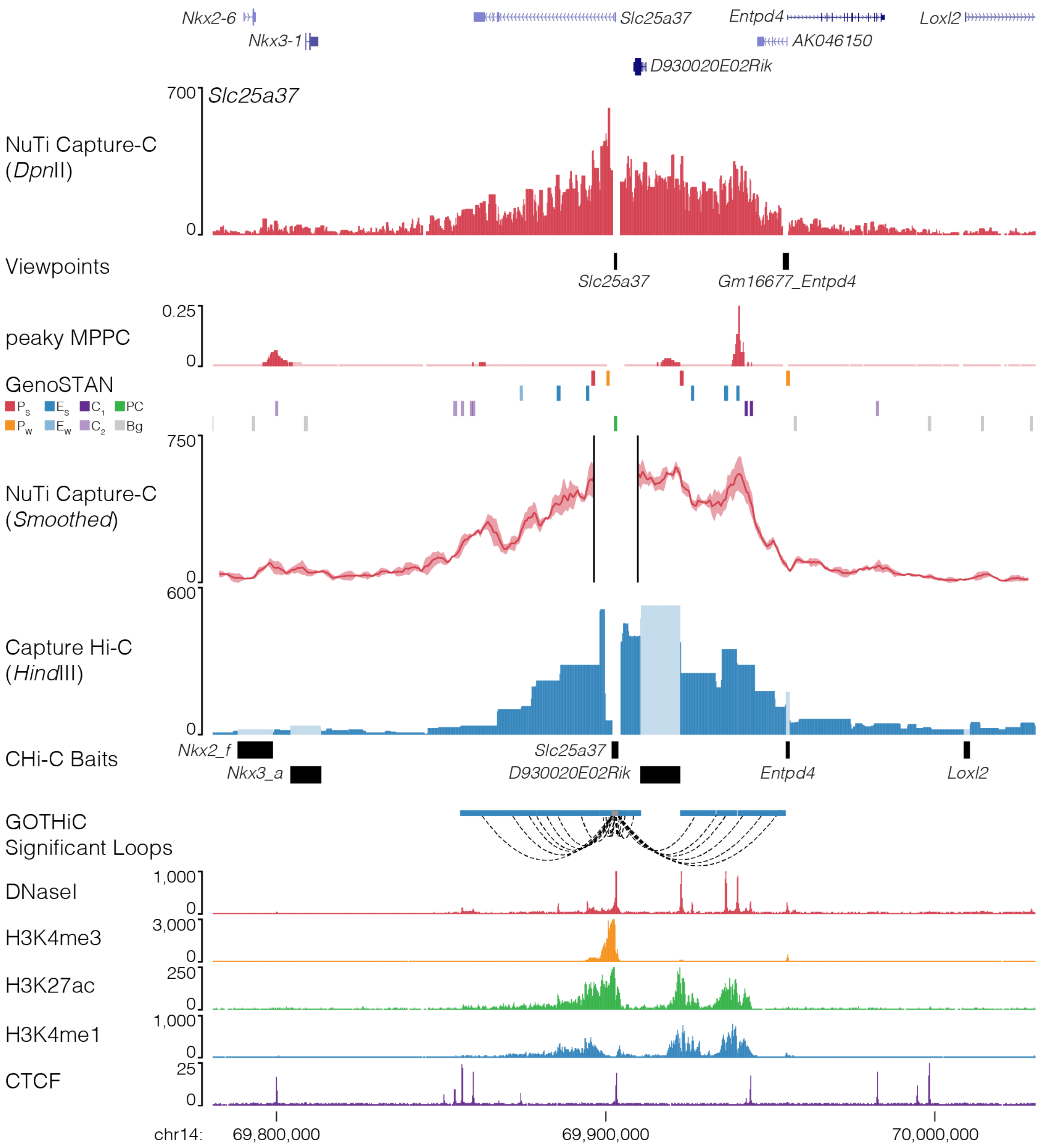

Supp. Fig. 25 I NuTi Capture-C from the *Slc25a37* promoter. Sequence tracks showing the difference between high-resolution 3C (*Dpn*II, NuTi Capture-C) and low-resolution 3C (*Hind*III, Capture Hi-C) at calling interacting fragments (mm9, chr14:69,780,624-70,030,623) in erythroid cells. Tracks in order: UCSC gene annotation, *cis*-normalized mean interactions per *Dpn*II fragment using NuTi Capture-C (n=3 independent 3C libraries), NuTi Capture-C viewpoints, peaky Marginal Posterior Probability of Contact (MPPC) scores with fragments with MPPC ≥0.01 darker, GenoSTAN open chromatin classification, 5 kb windowed mean interactions using NuTi Capture-C (smoothed), total supporting reads per *Hind*III fragment with CHi-C (n=2; co-targeted fragments are lighter in colour), CHi-C bait fragments, loops between reported significantly interacting fragments (co-targeting loops are coloured grey), erythroid tracks for open chromatin (DNaseI), promoters (H3K4me3), active transcription (H3K27ac), enhancers (H3K4me1), and boundaries (CTCF). Note overlapping MPPC signals appear darker in colour.

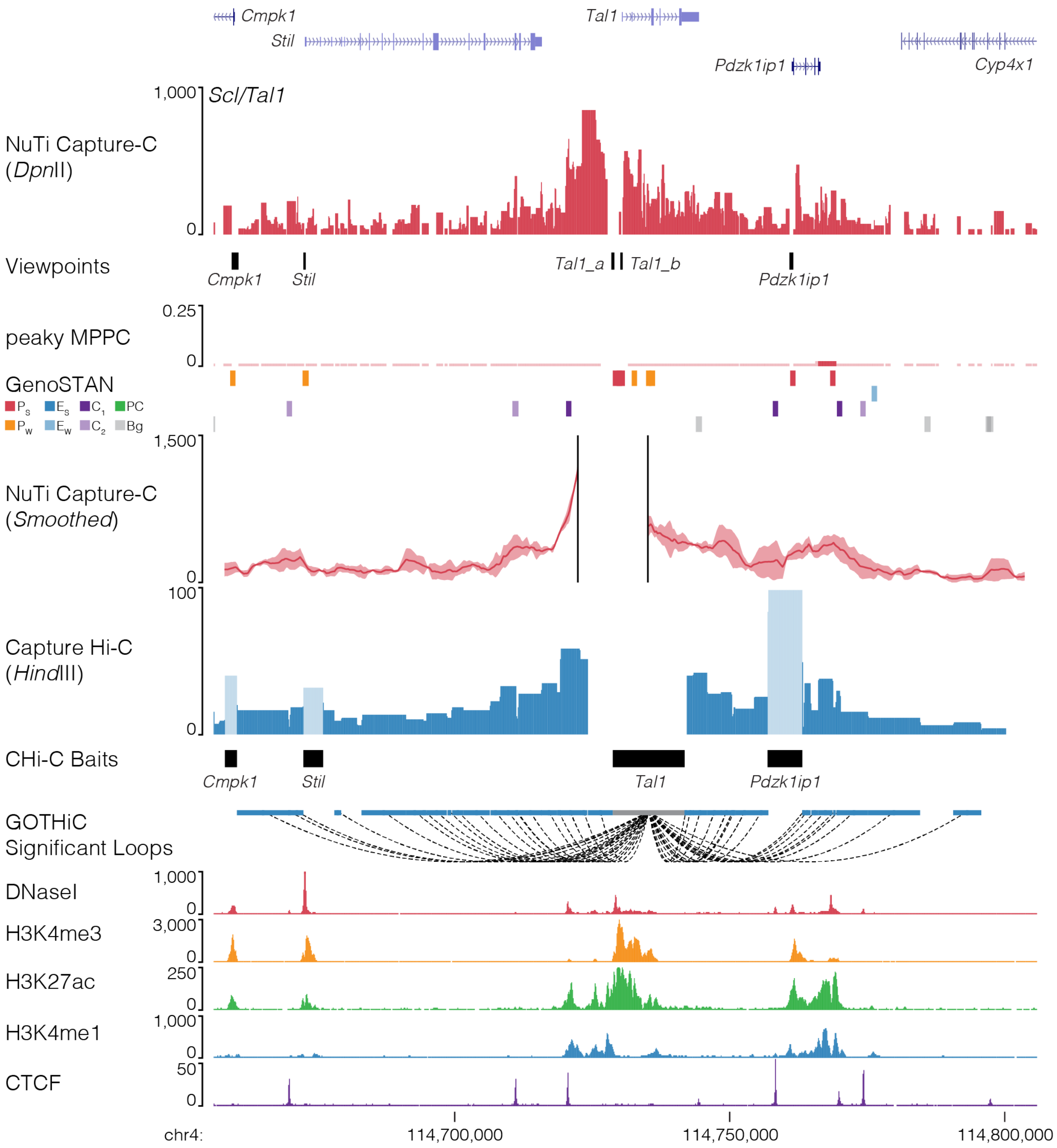

Supp. Fig. 26 | NuTi Capture-C from the *Tal1* promoter. Sequence tracks showing the difference between high-resolution 3C (*Dpn*II, NuTi Capture-C) and low-resolution 3C (*Hind*III, Capture Hi-C) at calling interacting fragments (mm9, chr4:114,656,021-114,806,020) in erythroid cells. Tracks in order: UCSC gene annotation, *cis*-normalized mean interactions per *Dpn*II fragment using NuTi Capture-C (n=3 independent 3C libraries), NuTi Capture-C viewpoints, peaky Marginal Posterior Probability of Contact (MPPC) scores with fragments with MPPC ≥0.01 darker, GenoSTAN open chromatin classification, 5 kb windowed mean interactions using NuTi Capture-C (smoothed), total supporting reads per *Hind*III fragment with CHi-C (n=2; co-targeted fragments are lighter in colour), CHi-C bait fragments, loops between reported significantly interacting fragments (co-targeting loops are coloured grey), erythroid tracks for open chromatin (DNaseI), promoters (H3K4me3), active transcription (H3K27ac), enhancers (H3K4me1), and boundaries (CTCF). Note overlapping MPPC signals appear darker in colour.

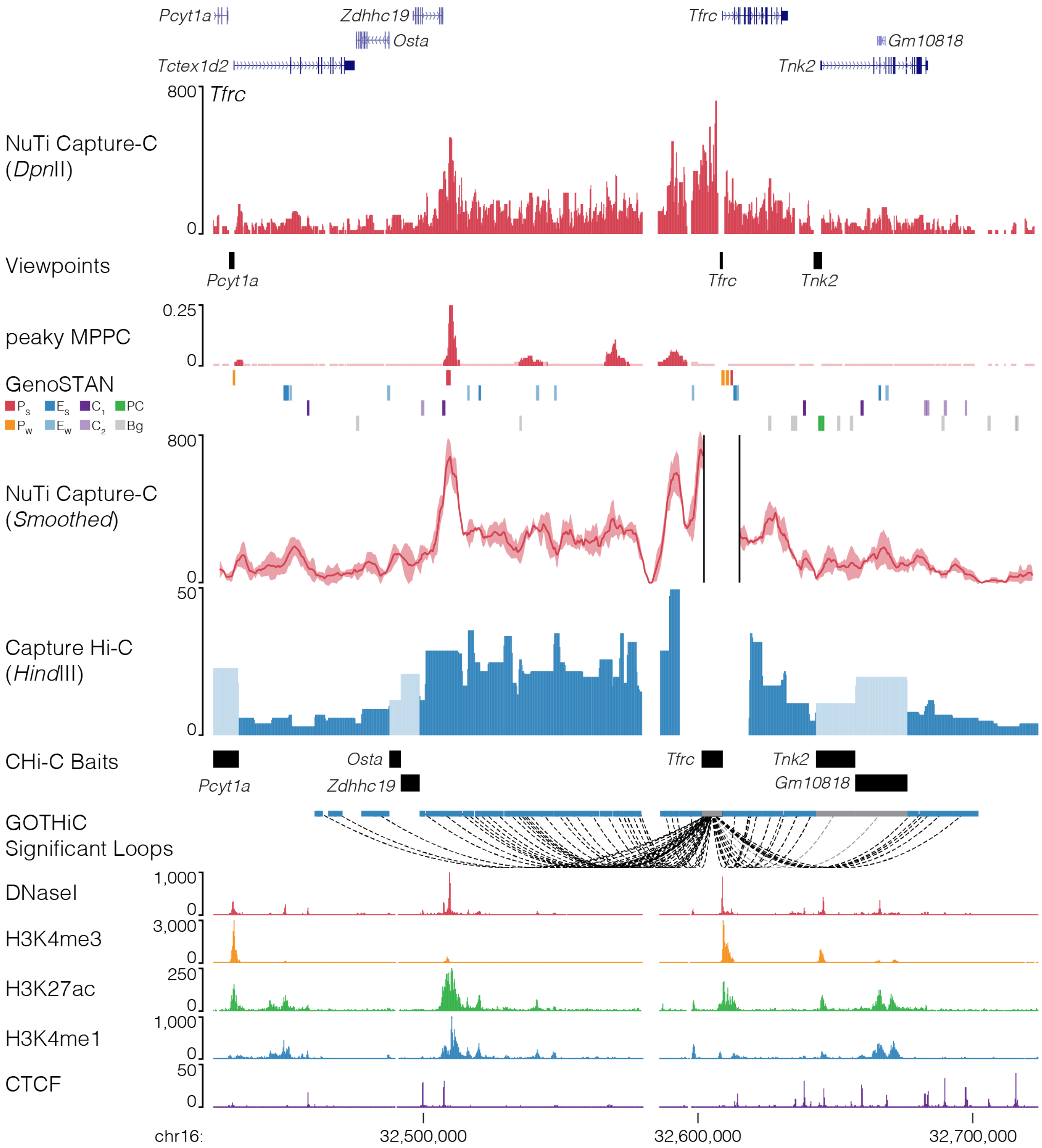

Supp. Fig. 27 I NuTi Capture-C from the *Tfrc* promoter. Sequence tracks showing the difference between high-resolution 3C (*Dpn*II, NuTi Capture-C) and low-resolution 3C (*Hind*III, Capture Hi-C) at calling interacting fragments (mm9, chr16:32,423,792-32,723,792) in erythroid cells. Tracks in order: UCSC gene annotation, *cis*-normalized mean interactions per *Dpn*II fragment using NuTi Capture-C (n=3 independent 3C libraries), NuTi Capture-C viewpoints, peaky Marginal Posterior Probability of Contact (MPPC) scores with fragments with MPPC ≥0.01 darker, GenoSTAN open chromatin classification, 5 kb windowed mean interactions using NuTi Capture-C (smoothed), total supporting reads per *Hind*III fragment with CHi-C (n=2; co-targeted fragments are lighter in colour), CHi-C bait fragments, loops between reported significantly interacting fragments (co-targeting loops are coloured grey), erythroid tracks for open chromatin (DNaseI), promoters (H3K4me3), active transcription (H3K27ac), enhancers (H3K4me1), and boundaries (CTCF). Note overlapping MPPC signals appear darker in colour.

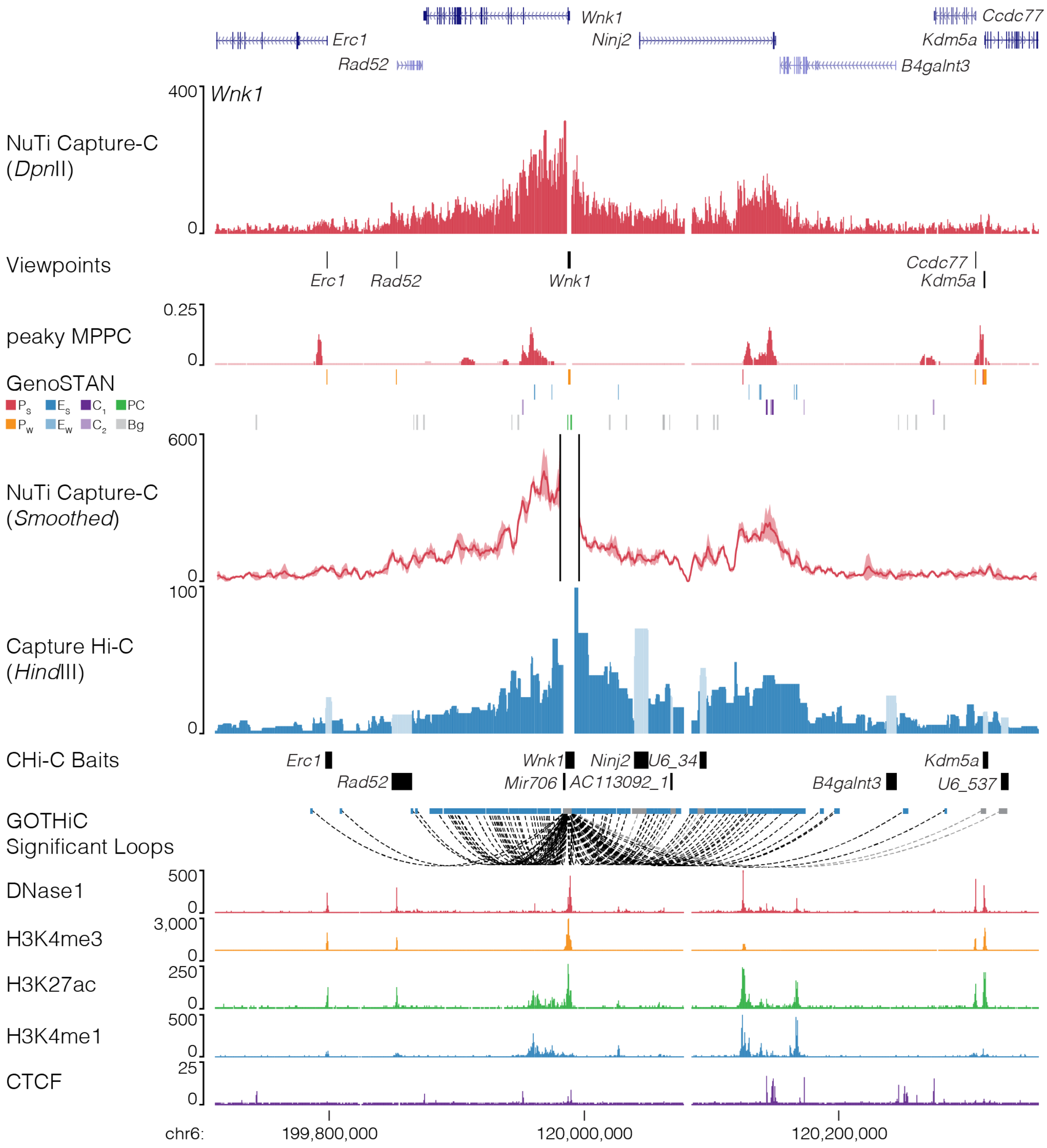

Supp. Fig. 28 l NuTi Capture-C from the *Wnk1* promoter. Sequence tracks showing the difference between high-resolution 3C (*Dpn*II, NuTi Capture-C) and low-resolution 3C (*Hind*III, Capture Hi-C) at calling interacting fragments (mm9, chr6:119,710,118-120,356,868) in erythroid cells. Tracks in order: UCSC gene annotation, *cis*-normalized mean interactions per *Dpn*II fragment using NuTi Capture-C (n=3 independent 3C libraries), NuTi Capture-C viewpoints, peaky Marginal Posterior Probability of Contact (MPPC) scores with fragments with MPPC ≥0.01 darker, GenoSTAN open chromatin classification, 5 kb windowed mean interactions using NuTi Capture-C (smoothed), total supporting reads per *Hind*III fragment with CHi-C (n=2; co-targeted fragments are lighter in colour), CHi-C bait fragments, loops between reported significantly interacting fragments (co-targeting loops are coloured grey), erythroid tracks for open chromatin (DNaseI), promoters (H3K4me3), active transcription (H3K27ac), enhancers (H3K4me1), and boundaries (CTCF). Note overlapping MPPC signals appear darker in colour.

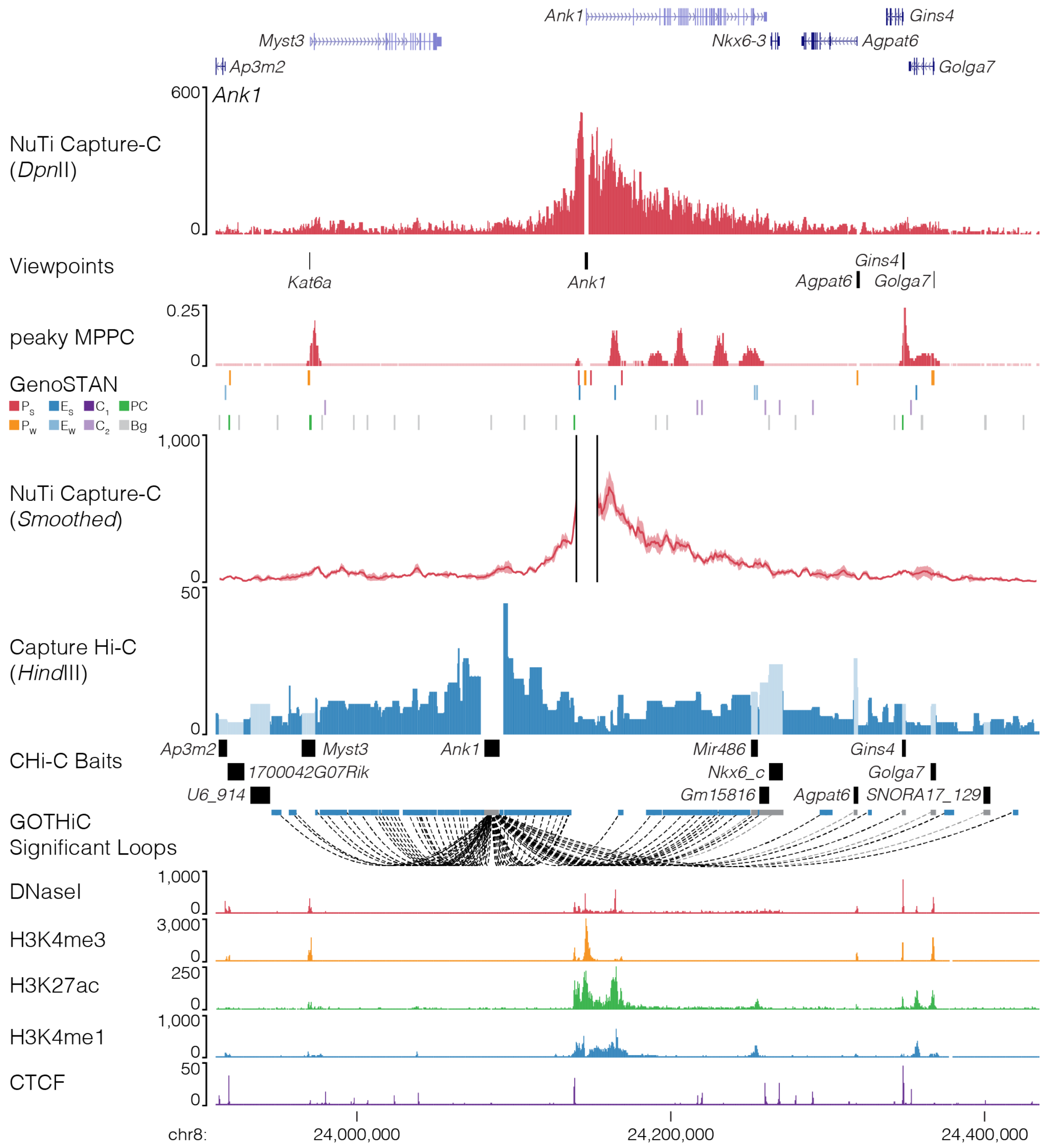

Supp. Fig. 29 | NuTi Capture-C from the *Ank1* promoter. Sequence tracks showing the importance of tissue specific probe design when performing promoter capture with either high-resolution 3C (*Dpn*II, NuTi Capture-C) or low-resolution 3C (*Hind*III, Capture Hi-C), particularly for genes with multiple promoters (mm9, chr8:23,910,000-24,435,000) in erythroid cells. Tracks in order: UCSC gene annotation, *cis*-normalized mean interactions per *Dpn*II fragment using NuTi Capture-C (n=3 independent 3C libraries), NuTi Capture-C viewpoints, peaky Marginal Posterior Probability of Contact (MPPC) scores with fragments with MPPC ≥0.01 darker, GenoSTAN open chromatin classification, 5 kb windowed mean interactions using NuTi Capture-C (smoothed), total supporting reads per *Hind*III fragment with CHi-C (n=2; co-targeted fragments are lighter in colour), CHi-C bait fragments, loops between reported significantly interacting fragments (co-targeting loops are coloured grey), erythroid tracks for open chromatin (DNaseI), promoters (H3K4me3), active transcription (H3K27ac), enhancers (H3K4me1), and boundaries (CTCF). Note overlapping MPPC signals appear darker in colour.

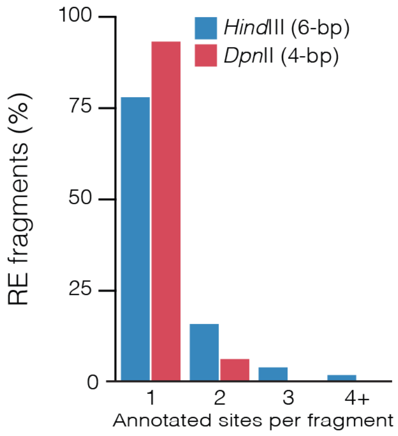

Supp. Fig. 30 | *Dpn*II provides higher resolution for distinguishing between functional elements than *Hind*III. Percent of restriction endonuclease fragments (RE) that overlap annotated erythroid open chromatin elements classified by the number of elements intersected per fragment.

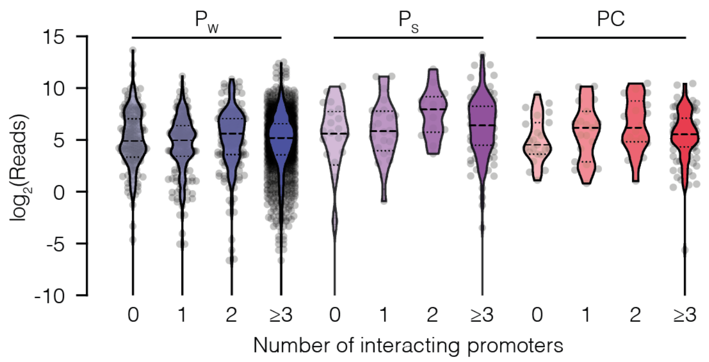

Supp. Fig. 31 | Promoter-hubs do not drive higher expression. Expression of enhancer interacting genes with promoters classified by GenoSTAN as having weak H3K27ac (P_W), strong H3K27ac (P_s) or a CTCF (PC). Genes are grouped on the number of promoters that they interact with. An equivalent analysis for genes without enhancer interactions is in Fig. 6.

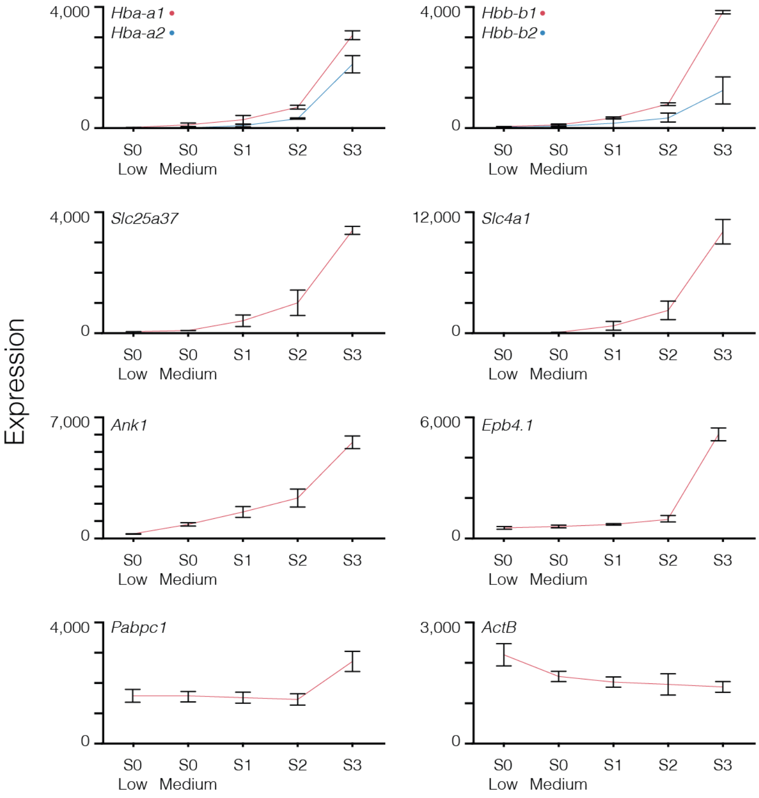

Supp. Fig. 32 I Expression of selected super-enhancer interacting genes. Nascent transcription of SE interacting genes was determined using 4sU-seq throughout erythroid differentiation from Haematopoietic Stem and Progenitor and Burst-Forming Unit-Erythroid cells (S0-Low), early and late Colony-Forming Unit-Erythroid cells (S0-Medium and S1 respectively), and maturing terminal differentiating cells (S2, S3). Error bars show standard error of the mean (n=3 independent experiments).
