# Supplementary material for: High-resolution targeted 3C interrogation of cis-regulatory element organization at genome-wide scale
Source: Nat Commun. 2021 Jan 22;12:531. doi: 10.1038/s41467-020-20809-6 (PMC7822813; doi:10.1038/s41467-020-20809-6)
Supplement: Supplementary file 2 — Supplementary Data 1 [file 41467_2020_20809_MOESM2_ESM.pdf]

Supplementary Data 1. 3C digestion efficiency primers

Assay Set	Sequence	Length	Tm	GC %	Amplicon
Dpn_cut_fwd	GGAGAAAGAAGGCTGGTGTTAT	22	62.23	45.45	105
Dpn_cut_rev	TATCTGAGTTGGACAGCATTGG	22	62.23	45.45	
Uncut_fwd	TTATCTTGCATTTGCCAACTCG	22	61.95	40.90	100
Uncut_rev	TGGGTTTCCCTGATTCTGAAA	21	61.84	42.85	
