# Supplementary material for: High-resolution targeted 3C interrogation of cis-regulatory element organization at genome-wide scale
Source: Nat Commun. 2021 Jan 22;12:531. doi: 10.1038/s41467-020-20809-6 (PMC7822813; doi:10.1038/s41467-020-20809-6)
Supplement: Supplementary file 4 — Description of Additional Supplementary Files [file 41467_2020_20809_MOESM4_ESM.pdf]

File Name: Supplementary Data 1.
Description: 3C Digestion efficiency qPCR primers for mouse.

File Name: Supplementary Data 2.
Description: Capture-C oligonucleotide sequences and locations.
