# Supplementary material for: High-resolution targeted 3C interrogation of cis-regulatory element organization at genome-wide scale
Source: Nat Commun. 2021 Jan 22;12:531. doi: 10.1038/s41467-020-20809-6 (PMC7822813; doi:10.1038/s41467-020-20809-6)
Supplement: Supplementary file 5 — Reporting Summary [file 41467_2020_20809_MOESM5_ESM.pdf]

nature research

Corresponding author(s): Jim Hughes

Last updated by author(s): Nov 30, 2020

Reporting Summary

Nature Research wishes to improve the reproducibility of the work that we publish. This form provides structure for consistency and transparency in reporting. For further information on Nature Research policies, see our Editorial Policies and the Editorial Policy Checklist.

Statistics

For all statistical analyses, confirm that the following items are present in the figure legend, table legend, main text, or Methods section.

n/a	Confirmed	
☐	☒	The exact sample size (*n*) for each experimental group/condition, given as a discrete number and unit of measurement
☐	☒	A statement on whether measurements were taken from distinct samples or whether the same sample was measured repeatedly
☐	☒	The statistical test(s) used AND whether they are one- or two-sided<br>*Only common tests should be described solely by name; describe more complex techniques in the Methods section.*
☒	☐	A description of all covariates tested
☐	☒	A description of any assumptions or corrections, such as tests of normality and adjustment for multiple comparisons
☐	☒	A full description of the statistical parameters including central tendency (e.g. means) or other basic estimates (e.g. regression coefficient) AND variation (e.g. standard deviation) or associated estimates of uncertainty (e.g. confidence intervals)
☐	☒	For null hypothesis testing, the test statistic (e.g. *F*, *t*, *r*) with confidence intervals, effect sizes, degrees of freedom and *P* value noted<br>*Give P values as exact values whenever suitable.*
☐	☒	For Bayesian analysis, information on the choice of priors and Markov chain Monte Carlo settings
☐	☒	For hierarchical and complex designs, identification of the appropriate level for tests and full reporting of outcomes
☐	☒	Estimates of effect sizes (e.g. Cohen's *d*, Pearson's *r*), indicating how they were calculated

Our web collection on statistics for biologists contains articles on many of the points above.

Software and code

Policy information about availability of computer code

Data collection	Data was collected with commercially available software<br>RT-qPCR: StepOnePlus™ Real-Time PCR System<br>Sequencing: NextSeq System Suite (v2)
Data analysis	Several previously published software packages were used for the analysis, custom codes have links to github repositories and preprint manuscripts where appropriate.<br><br>FASTQC (v0.11.4), Bowtie (v1.1.2), Samtools (v0.1.19), Bedtools (v2.25.0), deepTools (v2.2.2), macs2 (v2.0.10), DEseq2 (v1.8.2), GenoSTAN (v1.2.0), Clustalω (v1.2) in MacVector (v15.0), B3Synth (v25.1), pheatmap (v1.0.8), R (v3.2.1),, Kallisto (v0.43), CCseqBasic (v5.0, github.com/Hughes-Genome-Group/CCseqBasic5), captureCompare (v1.0, github.com/Hughes-Genome-Group/CaptureCompare), NGseqBasic (v20; https://www.biorxiv.org/content/early/2018/08/16/393413), peaky (https://github.com/cqgd/pky; doi: 10.1186/s12864-018-5314-5), Prism (v8.0e; https://www.graphpad.com/scientific-software/prism/)

For manuscripts utilizing custom algorithms or software that are central to the research but not yet described in published literature, software must be made available to editors and reviewers. We strongly encourage code deposition in a community repository (e.g. GitHub). See the Nature Research guidelines for submitting code & software for further information.

Data

Policy information about availability of data

All manuscripts must include a data availability statement. This statement should provide the following information, where applicable:

- Accession codes, unique identifiers, or web links for publicly available datasets
- A list of figures that have associated raw data
- A description of any restrictions on data availability

Sequence reads and processed data for the active gene capture, sequencing depth capture, and expression data have been archived in the Gene Expression Omnibus (GSE160229 [https://www.ncbi.nlm.nih.gov/geo/query/acc.cgi?acc=GSE160229], GSE129378 [https://www.ncbi.nlm.nih.gov/geo/query/acc.cgi?acc=129378] and GSE159229 [https://www.ncbi.nlm.nih.gov/geo/query/acc.cgi?acc=GSE159229] respectively). Source Data for all figures are provided in Supplemental Table 3. All other data supporting the findings of this study, including raw data files for optimization experiments are available from the corresponding author on request. Profiles for interactions of active genes in mouse erythroid cells are available at https://capturesee.molbiol.ox.ac.uk/projects/capture_compare/1086.

Field-specific reporting

Please select the one below that is the best fit for your research. If you are not sure, read the appropriate sections before making your selection.

☒ Life sciences ☐ Behavioural & social sciences ☐ Ecological, evolutionary & environmental sciences

For a reference copy of the document with all sections, see nature.com/documents/nr-reporting-summary-flat.pdf

Life sciences study design

All studies must disclose on these points even when the disclosure is negative.

Sample size	Sample size was chosen without statistical methods in line with similar analyses in past works to determine similar size differences in library quality. To allow statistical comparison a minimum of 2 viewpoints was used for all samples to unsure library quality and 3 or more replicates were used.
Data exclusions	No data were excluded.
Replication	Experiments were performed in duplicate or triplicate and correlation analysis performed to ensure high degree of reproducibility which was achieved for all experiments
Randomization	Randomization was not applied for assignment to groupings as they were either natural, categorical or inherent. For comparison of in situ and nuclear 3C, a single fixed aliquot was divided across all test conditions. When testing capture conditions (probe length, concentration, co-capture bias) experiments were performed on identical 3C libraries bearing different indices.
Blinding	No bliding was performed as analyses were performed using standardised bioinformatics pipelines in which all samples are treated equally without experimenter bias.

Reporting for specific materials, systems and methods

We require information from authors about some types of materials, experimental systems and methods used in many studies. Here, indicate whether each material, system or method listed is relevant to your study. If you are not sure if a list item applies to your research, read the appropriate section before selecting a response.

Materials & experimental systems

n/a	Involved in the study	
☒	☐	Antibodies
☐	☒	Eukaryotic cell lines
☒	☐	Palaeontology and archaeology
☐	☒	Animals and other organisms
☒	☐	Human research participants
☒	☐	Clinical data
☒	☐	Dual use research of concern

Methods

n/a	Involved in the study	
☒	☐	ChIP-seq
☒	☐	Flow cytometry
☒	☐	MRI-based neuroimaging

Eukaryotic cell lines

Policy information about cell lines

Cell line source(s)	Mouse embryonic stem cells (ESC) from the feeder free line ES-E14TGA2a.IV (Strain 129/Ola)
Authentication	No authentication was performed.
Mycoplasma contamination	Cell lines were routinely tested for mycoplasma infection and only negatively testing samples used for experiments.
Commonly misidentified lines (See ICLAC register)	No commonly misidentified lines were used.

Animals and other organisms

Policy information about studies involving animals; ARRIVE guidelines recommended for reporting animal research

Laboratory animals	Murine erythroid cells were obtained from spleens of fewer than five C57BL/6 and three C57BL/6-cross-CBA/J F1 hybrid mice of mixed genders (<1 year old). Fetal livers were isolated from a total of 47 embryos from 7 mice (C57BL/6). Experimental procedures were conducted under project licence 30/3339. All animals were singly housed, provided with food and water ad libitum, and maintained on a 12 h light: 12 h dark cycle (150-200 lux cool white LED light, measured at the cage floor). Temperature: 21 degrees +/- 3 degrees. Humidity: 55 +/10%
Wild animals	No wild animals were used.
Field-collected samples	No field-collected samples were used.
Ethics oversight	Protocols were approved through the Oxford University Local Ethical Review process. Experimental procedures were performed in accordance with European Union Directive 2010/63/EU and/or the UK Animals (Scientific Procedures) Act, 1986.

Note that full information on the approval of the study protocol must also be provided in the manuscript.
